# *Lactobacillus salivarius* GZPH2 reshapes hepatopancreatic microbiome structure and enhances immunometabolism in *Litopenaeus vannamei* under farm conditions

**DOI:** 10.3389/fmicb.2026.1762396

**Published:** 2026-04-16

**Authors:** Farhana Najnine, Xinbo Guo, Junpeng Cai

**Affiliations:** School of Food Science and Engineering, South China University of Technology, Guangzhou, China

**Keywords:** aquaculture, hepatopancreas, *Lactobacillus salivarius*, *Litopenaeus vannamei*, microbiome modulation, multi-omics, probiotic, proteomic profiling

## Abstract

**Introduction:**

The hepatopancreas of *Litopenaeus vannamei* plays a central role in digestion, metabolism, mineral homeostasis, and immune defense; however, its strain-specific responsiveness to probiotics remains insufficiently characterized. This study aimed to elucidate the comparative effects of a single-strain probiotic (Lactobacillus salivarius GZPH2; HH) and a mixed-strain consortium (EM; TH) on hepatopancreatic function under tropical semi-intensive culture conditions.

**Methods:**

An integrated multi-omics approach, combining histology, mineral profiling, 16S/18S rRNA sequencing, and 4D data-independent acquisition (4D-DIA) proteomics, was applied to evaluate probiotic-induced changes after 90 days of feeding, with a non-supplemented group (WH) as control.

**Results:**

Both probiotics significantly improved growth, survival, and feed efficiency, increasing biomass by 26–27% relative to the control; however, distinct mechanistic responses were observed. HH enhanced hepatopancreatic regeneration by increasing embryonic (E) and fibrillar (F) cells while reducing blister-like (B) and resorptive (R) cells, alongside greater accumulation of Mg, Fe, Ca, and Se. It also promoted microbial evenness and enriched beneficial *Alphaproteobacteria* (e.g., *Labrenzia, Tropicibacter*) and fungal taxa (*Candida–Lodderomyces clade*). Proteomic analysis revealed upregulation of carbohydrate metabolism, calcium regulation, immune-related proteins, and antioxidant enzymes, including hemocyanin, crustin-like proteins, chitinase, and catalase. In contrast, TH maintained a storage-oriented morphology, exhibited lower mineral deposition and microbial diversity, was dominated by *Bacillus*, and preferentially enriched proteolytic enzymes and redox-related pathways.

**Discussion:**

These findings demonstrate that the single-strain probiotic GZPH2 induced a more regenerative, metabolically efficient, and immunologically robust hepatopancreatic state than the mixed consortium. These findings provide multi-omics evidence supporting strain-specific probiotic selection as a precision strategy to enhance shrimp health and sustainability in aquaculture.

## 1 Introduction

Shrimp aquaculture is a major pillar of global seafood production, yet its long-term sustainability is increasingly challenged by climate instability, pathogen emergence, and intensification-driven stress. China, the world’s largest producer of *Litopenaeus vannamei* (*L. vannamei*), farming systems span temperate, subtropical, and tropical climatic zones, exposing cultured stocks to pronounced environmental gradients (Yao et al., 2008; Zhong et al., 2023; Chen et al., 2025). For example, in 2018 Guangdong achieved 6,110,250 tons of *L. vannamei*, a 9.91% annual increase, while Hainan’s production declined by 8.15%, yielding only 106,679 tons (Bureau of Fisheries of the Ministry of Agriculture and Rural Affairs et al., 2018). Regional climate sensitivity is further highlighted by an 80% mortality event in Hainan in June 2019, attributed to *Photobacterium damselae* subsp. *damselae*, a pathogen whose virulence intensifies under high temperatures (Wang et al., 2020). These climate-sensitive mortality events underscore a central challenge in contemporary shrimp production: enhancing host physiological resilience under high-temperature, production-scale pond conditions.

At the core of shrimp health lies the hepatopancreas, a multifunctional organ integrating digestion, nutrient assimilation, xenobiotic detoxification, metabolic regulation, mineral homeostasis, and immune defense (Sonakowska et al., 2016; Hernández-Cabanyero et al., 2023). Hepatopancreatic dysfunction is a defining feature of major diseases such as acute hepatopancreatic necrosis disease and *Enterocytozoon hepatopenaei* infection, both characterized by epithelial degeneration, metabolic collapse, and high mortality (Cao et al., 2023; Cheng et al., 2025; Haridevamuthu et al., 2025). Importantly, these pathologies are frequently accompanied by microbial dysbiosis and metabolic imbalance, highlighting tight coupling between microbiome stability and hepatopancreatic function (Velázquez-Lizárraga et al., 2019; Hossain et al., 2021). Despite its integrative physiological role, the hepatopancreas remains mechanistically underexplored in probiotic research conducted under realistic production conditions.

Probiotics are widely promoted as sustainable alternatives to antibiotics in aquaculture. Numerous studies report improvements in growth performance, immune competence, antioxidant capacity, and water quality, often linked to microbiome stabilization and modulation of host immune- and stress-related protein expression (Kumar et al., 2023; Gadhiya et al., 2025; Yang et al., 2024). Emerging evidence further suggests that probiotic supplementation can influence mineral transporters, detoxification systems, and redox pathways key determinants of hepatopancreatic resilience under environmental stress (Duan et al., 2024; Arumugam et al., 2025; Duan L. et al., 2025; Duan D. et al., 2025; Duan Y. et al., 2025; Oros et al., 2025). However, most aquaculture applications rely on mixed-strain formulations, typically comprising undefined combinations of *Lactobacillus*, *Bacillus*, and other effective microorganisms (EM) (Wang et al., 2019; Ghosh, 2023; Cheng et al., 2025). While such mixtures may provide broad-spectrum benefits (Rahayu et al., 2024; Cheng et al., 2025), their compositional complexity obscures mechanistic interpretation and introduces risks of interstrain antagonism, batch variability, and inconsistent field performance. Mixed-strain probiotics have often been reported to enhance shrimp growth and health (Wang et al., 2019; Amjad et al., 2022; Yu et al., 2025), yet single-strain formulations offer distinct advantages, including controlled dosing, reproducibility, and reduced antagonistic interactions, thereby enabling clearer mechanistic dissection of host–microbe interactions (Hai, 2015; Newaj-Fyzul and Austin, 2015). Meta-analytical evidence further indicates that multi-strain mixtures are not consistently superior to single strains (McFarland, 2021), underscoring the need for rigorous, strain-specific evaluation under field-relevant conditions.

Among promising candidates, *Lactobacillus salivarius* (*L. salivarius*) has demonstrated immunomodulatory and growth-promoting effects in poultry and swine (Sun et al., 2020; Chen et al., 2022; Dong P. et al., 2025; Dong X. et al., 2025). Laboratory studies in aquatic organisms report enhanced survival, pathogen resistance, and antioxidant capacity following supplementation (Talpur et al., 2011; Ljubobratovic et al., 2017; Mayor et al., 2019). However, field-level validation of *L. salivarius* in shrimp aquaculture remains absent, and its capacity to remodel hepatopancreatic functional networks under production level conditions has not been systematically investigated. This absence of mechanistic, field-based validation represents a major barrier to rational probiotic deployment in climate-sensitive aquaculture systems.

Recent advances in multi-omics technologies now enable integrated profiling of microbial community dynamics and host molecular responses (Zhang et al., 2022; Duan L. et al., 2025; Duan D. et al., 2025; Duan Y. et al., 2025; Ni et al., 2025). Although 16S rRNA sequencing and quantitative proteomics have been independently applied in shrimp research (Imaizumi et al., 2021; Zeng et al., 2024; Rajonhson et al., 2024; Dong P. et al., 2025; Dong X. et al., 2025), most studies assess isolated endpoints such as digestive enzymes, immune markers, or pathogen resistance without resolving how probiotics coordinately restructure microbiome networks and host proteomic architecture (Wang et al., 2022; Liang et al., 2025; Aldersey et al., 2026). Consequently, an integrated, comparative multi-omics investigation linking probiotic-induced microbiome restructuring to hepatopancreatic proteomic reprogramming under real farming conditions remains lacking.

To address this gap, we hypothesized that a defined single-strain probiotic (*L. salivarius*) would exert more targeted and mechanistically coherent effects on hepatopancreatic networks than a commercially available mixed-strain EM probiotic or a non-supplemented control. We therefore implemented a field-based comparative multi-omics framework integrating hepatopancreatic microbiome profiling (16S and 18S rRNA gene sequencing), quantitative 4D-data-independent acquisition (4D-DIA) proteomics, and histological assessment. Given that hepatopancreatic health is central to shrimp growth, development, and production performance, our objectives were to (i) evaluate improvements in tissue integrity, reflected by increased abundance of specialized hepatopancreatic cell types; (ii) characterize strain-specific alterations in hepatopancreatic microbial diversity and community structure and their role in hepatopancreatese; and (iii) identify differentially expressed proteins (DEPs) to elucidate probiotic strain-specific modulation of hepatopancreatic functional networks, including metabolic regulation, detoxification, mineral transport, redox homeostasis, and immune pathways. By resolving strain-specific functional signatures through a systems-level approach, this study presents one of the first comprehensive multi-omics comparisons of defined probiotic strategies using *L. sali*var*ius*. Conducted under real-world production conditions, the research elucidates how these strains modulate shrimp hepatopancreas function, thereby establishing a mechanistic foundation for precision microbiome interventions in sustainable shrimp aquaculture.

## 2 Materials and methods

### 2.1 Experimental site and environmental conditions

A 90-day production-level trial was conducted at a semi-intensive aquaculture facility in Haikou City, Hainan Province. The trial ran from 28 April to 28 July 2023. The site was selected for its tropical monsoon climate, enabling an evaluation of probiotic efficacy under realistic farming conditions with natural environmental conditions. Monthly meteorological data (temperature and precipitation) were recorded throughout the experimental period using the Microsoft News weather forecast website^1^ (Supplementary Table 1). The climate is characterized by consistently high year-round temperatures (summer: 30–33 °C; winter: 11–17 °C) and variable annual precipitation (2.58 to 78.85 mm).

### 2.2 Experimental design and pond system

The study employed a completely randomized design with three treatments, each replicated in three ponds (*n* = 9 ponds total) (Supplementary Figure 1). The experimental system consisted of nine uniform, rectangular earthen ponds (60 m^2^ each), arranged in three rows to prevent cross-contamination. To ensure independent water management, separate supply and drainage canals were used. Each pond was equipped with an independent aerator and a water pump. To mitigate soil erosion, all ponds were lined with HDPE plastic geomembranes. The pond profile featured a dike height of 0.8–1.2 m bottom slope 0.4 m, and maintained water depth (0.6–0.9 m) (Supplementary Figure 1b). The ponds were assigned to the following three treatment groups with three replicates groups: Control (Group W): Fed a basal diet without any probiotic supplementation (ponds WS1, WS2, and WS3). Commercial Probiotic (Group T): Fed the basal diet supplemented with a commercially used *Lactobacillus*-based mixed-strain effective microorganisms (EM) formulation (ponds TS1, TS2, and TS3). Experimental Probiotic (Group H): Fed the basal diet supplemented with *L. salivarius* strain GZPH2 (ponds HS1, HS2, and HS3).

### 2.3 Shrimp stocking, husbandry, and probiotic feeding regimen

Juvenile Pacific white shrimp (*L. vannamei*) at the PL10 stage were stocked into 60 m^2^ earthen ponds at a commercial density of 120 shrimp m^−2^ (7,200 individuals pond^−1^) and reared for 90 days under semi-intensive culture conditions. Throughout the experimental period, shrimp were fed a commercially formulated high-protein diet (Guangdong Yuehai Feed Group Co., China) three to four times daily in accordance with standard commercial aquaculture practice. The diet contained ≥42% crude protein, ≥4% crude lipid, ≥10.75% carbohydrates, ≤5% crude fiber, ≥2.25% lysine, ≥1% total phosphorus, ≤4% calcium, ≤3% NaCl, ≤16% ash, and ≤12% moisture.

Initial water quality parameters were: pH 7.50, total ammonia nitrogen (TAN) 0.82 mg L^−1^, NO₂^−^–N 0.08 mg L^−1^, and NO₃^−^–N 4.0 mg L^−1^, determined colorimetrically. Salinity was maintained at 15 ppt, and 30% of pond water was exchanged weekly to maintain nitrogen balance and environmental stability. No disease outbreaks were observed during the trial. Temporal dynamics of pH and nitrogenous compounds are provided in Supplementary Tables 2, 3.

Standing biomass was estimated weekly using a spatially randomized sampling framework to minimize within-pond heterogeneity bias. Shrimp were collected from three randomly selected pond locations using a cast net, and 50 individuals were randomly subsampled and weighed individually (precision ±0.01 g) to determine mean body weight (MBW). Shrimp were immediately returned to the pond to minimize handling stress.

Biomass (kg) was calculated as:


$$\text{Biomass}=\frac{\text{Mean body weight}\;(g)\times \text{Estimated surviving population}}{1,000}$$

where division by 1,000 converts grams to kilograms. Survival was updated weekly based on direct mortality counts and validated at final harvest by total enumeration. Feeding rates were adjusted weekly based on estimated biomass and feed tray monitoring, beginning at 8–10% of biomass during early growth and gradually reduced to 3–5% as shrimp increased in size. Final biomass and survival were confirmed by complete harvest, ensuring accurate feed conversion ratio (FCR) calculation (see Supplementary note 1). Probiotic treatments were administered via feed supplementation according to the experimental design (see Supplementary Figures 2, 3 and Supplementary note 2).

### 2.4 Sample collection

Upon harvest at the end of the 90-day experiment, shrimp were transported live from the facility to the laboratory for analysis. From each replicate pond, 25 visibly healthy shrimp without any physical damage were randomly selected. These shrimp were placed in polyethylene bags filled with oxygenated water (maintaining a dissolved oxygen level of 5 mg/L). The bags were then secured in a temperature-controlled, insulated container (a rectangular cool-sheet box) to maintain a stable temperature during transport, which lasted less than 72 h. Immediately upon arrival, the containers were unsealed, and shrimp vitality was assessed. All specimens exhibited active gill movement and a robust response to gentle physical stimulation, confirming they were alive and free from overt physical impairment.

### 2.5 Histological analysis

Three adult *L. vannamei* from each treatment were euthanized by thermal shock. The hepatopancreas was dissected and fixed in Bouin’s solution for 24 h. Samples were coded according to pond origin and tissue type: WH, HH, and TH. Tissues were processed through graded alcohol dehydration, cleared in xylene, and embedded in paraffin at 65 °C. Blocks were cooled to −20 °C, sectioned at 2–4 μm, mounted on slides, and dried at 60 °C. Sections were deparaffinized, rehydrated, and stained with hematoxylin and eosin (H&E). After dehydration and clearing, slides were mounted with neutral gum.

Histological observations were performed using an upright light microscope (Nikon, Japan) equipped with a digital camera, and images were captured using Slideviewer software (v2.5). Hepatopancreatic tubule length and width were measured, and the proportions of E-cells (embryonic cells), R-cells (resorptive cells), F-cells (fibrillar cells), B-cells (blister-like cells), and M-cells (microfold/midgut cells) were quantified across proximal, middle, and distal regions. Ten digital images per sample were analyzed to evaluate structural and cellular alterations under experimental conditions.

### 2.6 Minerals analysis

The mineral content of shrimp hepatopancreas from three sample groups (WH, HH, and TH), was evaluated using a technique described by Liu et al. (2021) using an inductively coupled plasma emission spectrometer (iCAP 7400 ICP-OES Analyzer, Thermo Fisher Scientific, United States). Each group was examined in triplicate. Approximately 5 g of each sample was placed in a polytetrafluoroethylene (PTFE) test tube (Ningbo Ja-Hely Technology Co., Ltd., China) and combined with 12 mL of concentrated nitric acid (68%) (Shijiazhuang Famechem Chemical Co., Ltd., China). The mixture was digested until colorless, cooled, transferred to a 50 mL volumetric flask, and diluted with deionized water to a specific volume. A blank experiment was also performed.

### 2.7 Illumina MiSeq high-throughput sequence analysis

Shrimp hepatopancreas tissue was aseptically dissected following surface sterilization with 75% ethanol and sterile distilled water. For each biological replicate, hepatopancreatic tissue from three shrimp was pooled and homogenized in sterile water. Total genomic DNA was extracted from the homogenates using the FAST DNA Spin Kit (MO BIO Laboratories) according to the manufacturer’s protocol. DNA concentration, purity, and integrity were assessed via NanoDrop 2000 spectrophotometry (Thermo Scientific) and 1% agarose gel electrophoresis. Qualified DNA was stored at −80 °C until further processing.

PCR amplification of the 16S rRNA V3–V4 and 18S rRNA V4 regions was performed on a GeneAmp 9,700 thermocycler (ABI) using primer pairs 338F/806R and 3NDF/V4-euk-R2, respectively, following the thermal cycling conditions detailed in previous studies (Xiong et al., 2017; Xiong et al., 2018). The 20 μL reaction mixture composition was as described by Yang et al. (2022). To minimize PCR bias, each sample was amplified in triplicate, and the pooled amplicons were purified using a QIAquick PCR Purification Kit (Qiagen).

Purified libraries were quantified, pooled in equimolar ratios, and sequenced on an Illumina MiSeq platform (Illumina) following the manufacturer’s guidelines. Sequence data were processed according to established protocols for 16S (Xiong et al., 2017) and 18S rRNA genes (Dai et al., 2017). Briefly, paired-end reads were merged using FLASH (Magoč and Salzberg, 2011) and processed with the QIIME pipeline (v1.9.0) (Caporaso et al., 2010). Sequences were quality-filtered; chimeras and singletons were identified and removed using UCHIME (Edgar et al., 2011). Operational taxonomic units (OTUs) were clustered at 97% similarity with UCLUST (Edgar, 2010). For prokaryotic (16S) data, taxonomic classification was performed against the Silva database (release 128), and non-prokaryotic OTUs and singletons were removed. Samples were rarefied to 42,239 sequences per sample for downstream analysis. For eukaryotic (18S) data, phylotypes were classified using the Silva database (release 119), with non-eukaryotic OTUs and singletons removed prior to rarefaction to 22,495 sequences per sample. The raw sequence data are available in the NCBI SRA under accession number PRJNA1158989.

### 2.8 4D data-independent acquisition proteomics analysis

Proteomic profiling of hepatopancreas samples was conducted by Shanghai Majorbio Bio-Pharm Technology Co., Ltd., following their established protocols (Ren et al., 2022). Hepatopancreas tissue was collected from five healthy shrimp per replicate group and preserved in phosphate-buffered saline (PBS) at −20 °C. Samples were thawed, homogenized in lysis buffer (8 M urea, 1% SDS) with protease inhibitor, and centrifuged, protein concentration was measured using the bicinchoninic acid (BCA) assay kit (Thermo Scientific). For digestion, 100 μg of protein was suspended in triethylammonium bicarbonate buffer (TEAB) then treated with tris (2-carboxyethyl) phosphine (TCEP) at 37 °C for 60 min, alkylated with iodoacetamide (IAM) in the dark, and digested with trypsin overnight at 37 °C. The peptides were desalted, dried, and quantified using a peptide quantification kit (Thermo Fisher Scientific).

Peptide analysis was performed on an Evosep-One system coupled to a TimsTOF Pro2 mass spectrometer (Bruker) operating in DIA-PASEF mode. Raw data were processed with Spectronaut software (v14) for protein identification and quantification, applying standard filters (e.g., protein FDR ≤0.01). Shared and modified peptides were excluded and proteins were identified using at least one unique peptide. The peak areas were summed for quantification. This method enables comprehensive proteomic profiling of shrimp hepatopancreas samples.

### 2.9 Statistical analysis

Data were analyzed using IBM SPSS version 26.0. Differences among the three groups were assessed using one-way ANOVA followed by Tukey’s *post-hoc* test, with significance set at *p* < 0.05. Pearson correlation coefficients (*r*) were also calculated. Results were presented as mean ± standard deviation from three experiments in tables, or as figures generated with R (version 4.3.2).

For microbiome analysis, alpha diversity indices (Coverage, ACE, Chao1, Shannon, Simpson) were calculated using Mothur (v.1.30.2) based on a homogenized ASV table. Beta diversity was evaluated using principal coordinate analysis (PCoA) and ANOSIM (999 permutations) with the vegan package in R (v.3.3.1).

Community composition and relative abundance were visualized using Venn diagrams, bar plots, and pie plots at various taxonomic levels. The Kruskal–Wallis *H* test and Tukey–Kramer test were used to determine statistical significance in species abundance differences among groups. Linear discriminant analysis (LDA) effect size (LEfSe) was employed to identify differentially abundant taxa.

### 2.10 Functional analysis

Functional analysis of 16S rRNA ASV and proteomic data was performed on the Majorbio Cloud platform.^2^

Microbial phenotypes were predicted from 16S rRNA ASV data using the BugBase platform. Following normalization by predicted 16S rRNA gene copy number, phenotypes were inferred, including Gram staining, biofilm formation, pathogenicity, oxygen tolerance, and oxidative stress. Additionally, 16S rRNA gene sequences were functionally annotated by matching them to Kyoto Encyclopedia of Genes and Genomes (KEGG) orthology identifiers (KO IDs) to map associated metabolic pathways.

Protein sequences were functionally annotated by searching against the KEGG, Evolutionary Gene Genealogy Non-supervised Orthologous Groups (EggNOG), Gene Ontology (GO), Protein Families Database (Pfam), and subcellular localization databases. Differentially expressed proteins (DEPs) between groups were identified based on |log2FC| ≥0.263 and *p* < 0.05. Enriched KEGG pathways among DEPs were determined using Fisher’s exact test with Benjamini–Hochberg correction (*p*-adjusted <0.05). Protein–protein interaction networks for DEPs were analyzed using the STRING database (v11.5) via REACTOME.

## 3 Results

### 3.1 Growth performance, survival, and feed utilization

Initial body weights did not differ among treatments (*p* > 0.05), confirming baseline homogeneity. After 90 days, probiotic supplementation significantly enhanced growth (Table 1; Supplementary Table 5). Final body weight was highest in TH, followed by HH, both exceeding WH (*p* < 0.05), with no difference between probiotic groups. Weight gain followed the same pattern, indicating a consistent growth-promoting effect. Survival was significantly improved in probiotic treatments, reaching 83% (HH) and 81% (TH) versus 75% in WH (*p* < 0.05). Consequently, final stock numbers and total biomass were markedly higher in HH and TH compared with WH, representing a 26–27% yield increase. Feed conversion ratio was significantly reduced in HH and TH relative to WH (*p* < 0.05), reflecting ~16–17% improved feed efficiency. Collectively, probiotics substantially enhanced production performance.

**Table 1:** Growth performance, survival, and feed utilization of shrimp.

Parameter	WH	HH	TH
Initial body weight (g)	0.100 ± 0.010	0.103 ± 0.010	0.100 ± 0.010
Final body weight (g)	21.33 ± 1.15ᶜ	24.41 ± 0.62ᵃ	24.85 ± 0.22ᵃ
Total weight gain (g)	21.23 ± 1.15ᶜ	24.31 ± 0.62ᵃ	24.75 ± 0.22ᵃ
Survival rate (%)	75.0 ± 1.20ᶜ	83.0 ± 3.08ᵃ	81.0 ± 1.90ᵇ
Initial stocking (shrimp/m^2^)	120	120	120
Initial shrimp number	7,200	7,200	7,200
Final shrimp number	5,429 ± 34^c^	5,980 ± 5^a^	5,849 ± 17^b^
Total biomass gain (kg)	115.26 ± 1.81^b^	145.37 ± 0.71^a^	144.76 ± 0.84^a^
Total feed input (kg)	216.9 ± 2.10^b^	230.4 ± 0.94^a^	228.3 ± 0.96^a^
Feed conversion ratio (FCR)	1.88 ± 0.01ᵃ	1.59 ± 0.04ᵇ	1.58 ± 0.03ᵇ

Data were shown as mean ± standard deviation (weight: *n* = 50 shrimp/group; other parameters: *n* = 3 ponds/group). Different letters in the same row indicate significant difference (*p* < 0.05), and same letters indicate no difference (*p* > 0.05).

### 3.2 Significant changes in morphological characteristics of shrimp hepatopancreas

**Figure 1:**
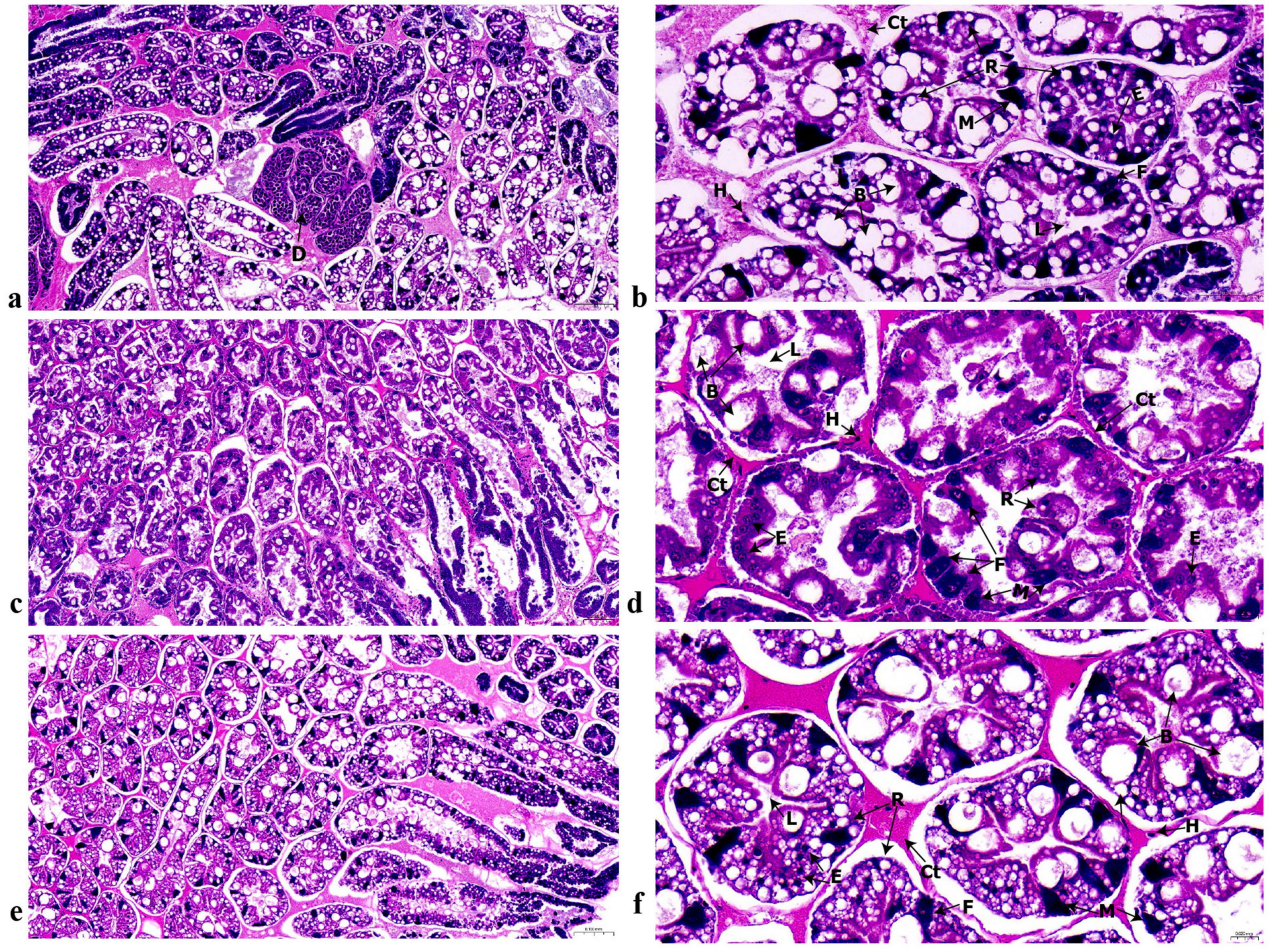
Longitudinal and transverse histological sections of hepatopancreatic tubules in the three shrimp groups (WH, HH, and TH). Micrographs display tissue from **(a,b)** WH group, **(c,d)** HH group, and **(e,f)** TH group. Cellular and structural features are labeled: E, E-cell; R, R-cell; F, F-cell; B, B-cell; M, M-cell; Ep, epithelium; L, lumen; Ct, connective tissue; H, hemolymph.

Histological analysis revealed clear strain-specific differences in hepatopancreatic structure. H&E-stained sections confirmed the typical pseudo-stratified epithelium of hepatopancreatic tubules, comprising B-, E-, F-, M-, and R-cells (Figure 1 and Table 2). In WH, the hepatopancreas exhibited partially disorganized tubules with poorly delineated epithelial boundaries. Quantitative assessment of longitudinal sections indicated that WH shrimp possessed a predominance of storage-type B-cells and R-cells (Table 2).

**Table 2:** Number of different cell types in longitudinal and transverse sections of the epithelium of hepatopancreatic tubules in three groups of shrimp.

	WH		HH		TH	
Cell types	Longitudinal section	Transverse section	Longitudinal section	Transverse section	Longitudinal section	Transverse section
B-cell	23.00 ± 4.16^a^	7.2 ± 1.64^a^	13.80 ± 4.76^b^	3.2 ± 1.09^c^	24.40 ± 3.56^a^	6.5 ± 1.38^b^
R-cell	56.00 ± 2.06^ab^	68.40 ± 14.77^ab^	44.20 ± 11.37^b^	48.4 ± 2.07^b^	64.80 ± 15.07^a^	74.40 ± 15.23^a^
E-cell	15.20 ± 3.56^b^	6.80 ± 2.16^b^	56.80 ± 16.84^a^	20.60 ± 6.65^a^	21.2 ± 20.24^ab^	7.6 ± 3.05^b^
F-cell	13.00 ± 3.93^b^	2.60 ± 1.67^a^	29.40 ± 17.27^a^	4.20 ± 1.48^a^	8.20 ± 1.92^c^	2.40 ± 1.14^a^
M-cell	20.40 ± 2.88^ab^	4.40 ± 1.14^a^	12.40 ± 5.98^b^	3.20 ± 1.30^a^	28.60 ± 6.38^b^	4.60 ± 0.54^a^

Data are shown as the mean ± standard deviation (*n* = 20). Different letters in the same row indicate significant differences (*p* < 0.05) and the same letters indicate no difference (*p* > 0.05).

Marked strain-specific differences were observed between the two probiotic groups (Table 2). In both longitudinal and transverse sections, the HH exhibited significantly higher proportions of regenerative E-cells (up to 2.68-fold) and secretory F-cells (up to 3.59-fold) compared to TH (*p* < 0.05). Conversely, TH consistently displayed a storage- and resorptive-dominant profile, with significantly greater abundances of B-cells, R-cells, and M-cells than HH (*p* < 0.05). Differences in B-, F-, and M-cells were less pronounced in transverse sections, although the trend for TH to retain higher storage-associated cell proportions remained.

### 3.3 Mineral composition of the hepatopancreas

The mineral composition of the hepatopancreas varied significantly among the experimental groups (Table 3). The WH and HH exhibited significantly higher concentrations of copper (Cu), selenium (Se), sodium (Na), and zinc (Zn) compared to the TH (*p* < 0.05). The WH also showed significantly elevated levels of potassium (K), and calcium (Ca) relative to the probiotic-treated groups (*p* < 0.05). In contrast, the HH demonstrated significantly higher iron (Fe), phosphorus (P) and magnesium (Mg) contents (*p* < 0.05). The TH exhibited significantly reduced levels of all analyzed minerals compared to both WH and HH (*p* < 0.05), indicating a lower mineral accumulation capacity in the hepatopancreas.

**Table 3:** Mineral composition (μg/g sample) of hepatopancreas samples from three groups of shrimp.

Basic nutrient	WH	HH	TH
Na	529.1 ± 52.71^a^	488.41 ± 79.13^a^	140.71 ± 14.7^b^
Se	508.96 ± 105.78^a^	469.73 ± 165.33^a^	203.2 ± 4.65^b^
P	483.05 ± 25.97^b^	829.51 ± 22.07^a^	220.04 ± 15.85^c^
Ca	185.25 ± 46.68^a^	136.62 ± 45.91^ab^	46.04 ± 9.59^b^
Mg	100.79 ± 15.38^b^	189.49 ± 31.60^a^	27.04 ± 1.80^c^
Fe	7.00 ± 1.02^a^	4.08 ± 2.15^b^	3.30 ± 0.17^b^
Cu	6.06 ± 0.59^a^	6.26 ± 1.87^a^	1.02 ± 0.01^b^
Zn	4.09 ± 1.35^a^	4.01 ± 1.08^a^	1.27 ± .09^b^
K (g/L)^a^	3.93 ± 0.8^a^	2.25 ± 2.12^ab^	1.36 ± 1.02^b^

All data are presented as mean ± standard deviation (*n* = 3). Different letters in the same column indicate significant differences (*p* < 0.05), whereas the same letters indicate no difference (*p* > 0.05). ^*^K concentration was higher, which is why the (g/L) measurement unit was used.

### 3.4 Microbial analysis

#### 3.4.1 Microbial diversity

Alpha diversity analysis revealed pronounced, treatment-dependent shifts in microbial richness and evenness among the WH, HH, and TH groups. In prokaryotic communities (Table 4), in the WH, despite exhibiting the highest sequencing depth (*p* > 0.05), whereas ASV richness increased by 2.9-fold in HH and 4.1-fold in TH, with corresponding increases in ACE and Chao1 indices (2.9-fold in HH and 4.2-fold in TH; *p* < 0.01), indicating a substantial expansion of microbial taxa, particularly in TH. Community diversity was similarly elevated, as the Shannon index increased by 3.3-fold in HH and 3.2-fold in TH, while the Simpson index declined to 0.34-fold and 0.53-fold, respectively, with the most pronounced reduction observed in HH.

**Table 4:** Bacterial diversity and richness indices for hepatopancreas samples from three groups of shrimp.

Group	Sequence number	ASV number	Shannon index	Simpson index	ACE Index	Chao1 index	Coverage
WH	47576.67 ± 3250.5^a^	45.67 ± 21.36^b^	0.68 ± 0.23^b^	0.7 ± 0.12^a^	45.3 ± 20.75^b^	45.11 ± 20.41^b^	0.99 ± 0.00^a^
HH	46645.67 ± 2880.42^a^	132.00 ± 45.21^ab^	2.27 ± 0.83^a^	0.24 ± 0.17^a^	132.17 ± 46.83^ab^	132.61 ± 47.68^ab^	0.99 ± 0.00^a^
TH	42379.00 ± 140.00^a^	188.00 ± 66.00^a^	2.19 ± 1.15^a^	0.37 ± 0.28^a^	188.72 ± 65.15^a^	187.32 ± 64.79^a^	0.99 ± 0.00^a^

All data are presented as mean ± standard deviation (*n* = 3). Different letters in the same column indicate significant differences (*p* < 0.05), whereas the same letters indicate no difference (*p* > 0.05).

In contrast, eukaryotic communities (Table 5) displayed contrasting responses: HH exhibited higher richness and diversity, with ASVs increasing by 1.36-fold, ACE and Chao1 by 1.65- and 1.36-fold, and the Shannon index by 1.12-fold. Conversely, TH demonstrated a simplified eukaryotic structure, characterized by reduced ASV richness (0.89-fold) and a sharp decline in Shannon diversity (0.51-fold), accompanied by a 2.39-fold increase in Simpson index, indicating greater dominance of fewer taxa.

**Table 5:** Eukaryotic diversity and richness indices for hepatopancreas samples from three groups of shrimp.

Group	Sequence number	ASV number	Shannon index	Simpson index	ACE index	Chao1 index	Coverage
WH	30,548 ± 5060.26^a^	24.00 ± 13.00^a^	2.16 ± 0.59^ab^	0.18 ± 0.06^b^	20.18 ± 18.57^a^	23.66 ± 12.50^a^	0.99 ± 0.00^b^
HH	30146.67 ± 7661.01^a^	32.67 ± 2.51^a^	2.42 ± 0.48^a^	0.16 ± 0.12^b^	33.29 ± 2.27^a^	32.22 ± 2.16^a^	0.99 ± 0.00^b^
TH	33033.33 ± 1889.5^a^	21.33 ± 3.51^a^	1.10 ± 0.15^b^	0.43 ± 0.02^a^	21.00 ± 3.00^a^	21.00 ± 3.00^a^	1.00 ± 0.00^a^

All data are shown as the mean ± standard deviation (*n* = 3). Different letters in the same column indicate significant difference (*p* < 0.05), whereas the same letters indicate no difference (*p* > 0.05).

**Figure 2:**
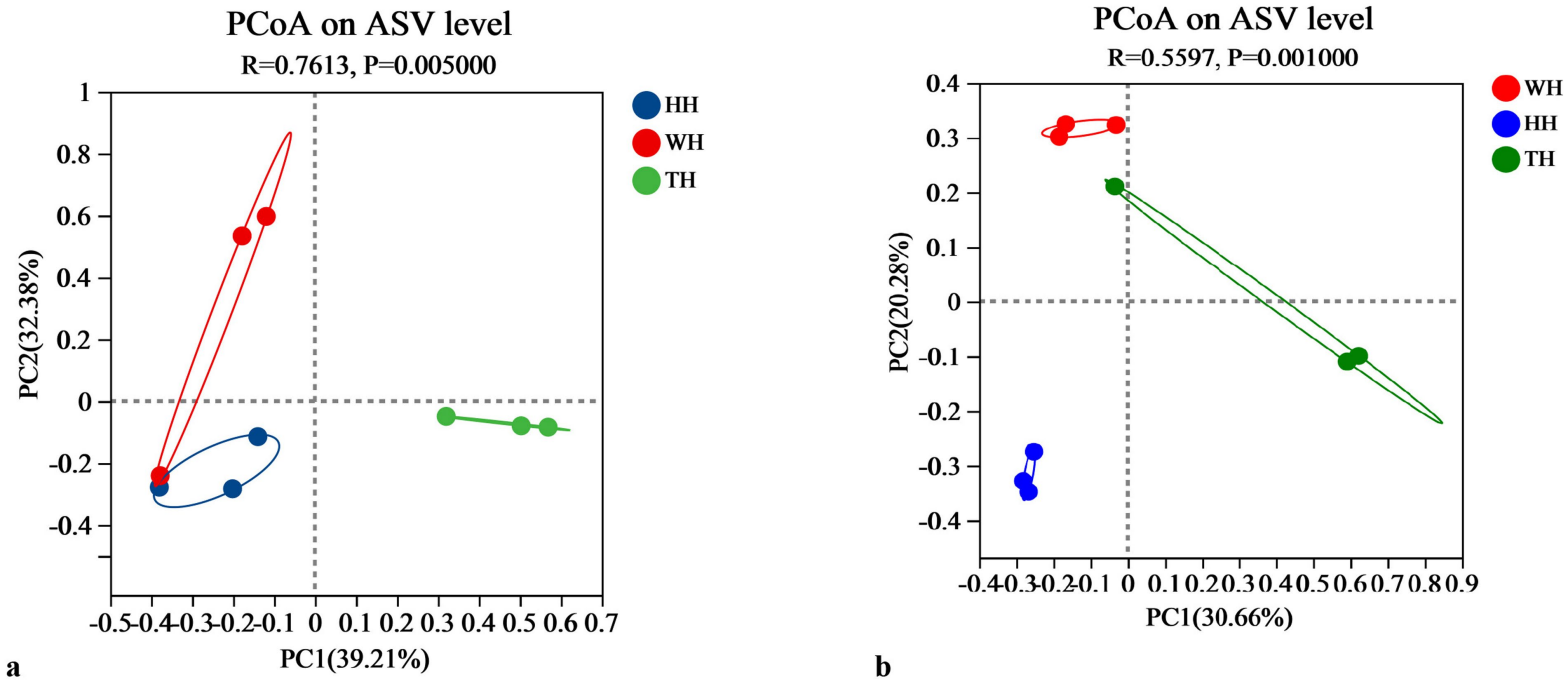
Beta-diversity analysis of hepatopancreas microbiota. Principal coordinate analysis (PCoA) plots based on Bray–Curtis distances for **(a)** prokaryotic communities (16S rRNA gene) and **(b)** eukaryotic communities (18S rRNA gene) across the three shrimp groups (WH, HH, and TH).

Beta diversity, which measures differences in microbial community composition between samples, provides key insights into the structural and functional variations within microbial ecosystems. In this study, beta diversity was assessed using principal coordinate analysis (PCoA) based on Bray–Curtis dissimilarity. For prokaryotic communities (16S rRNA gene), PCoA revealed significant clustering (ANOSIM, *R* = 0.7613, *p* = 0.005), with clear separation between the probiotic-supplemented groups (HH and TH) and the control (WH) (Figure 2a). Similarly, eukaryotic communities (18S rRNA gene) exhibited distinct clustering (ANOSIM, *R* = 0.5597, *p* = 0.001), demonstrating that both probiotic treatment and climatic conditions significantly influenced the hepatopancreas microbiota composition (Figure 2b).

#### 3.4.2 Prokaryotic community structure in the hepatopancreas

**Figure 3:**
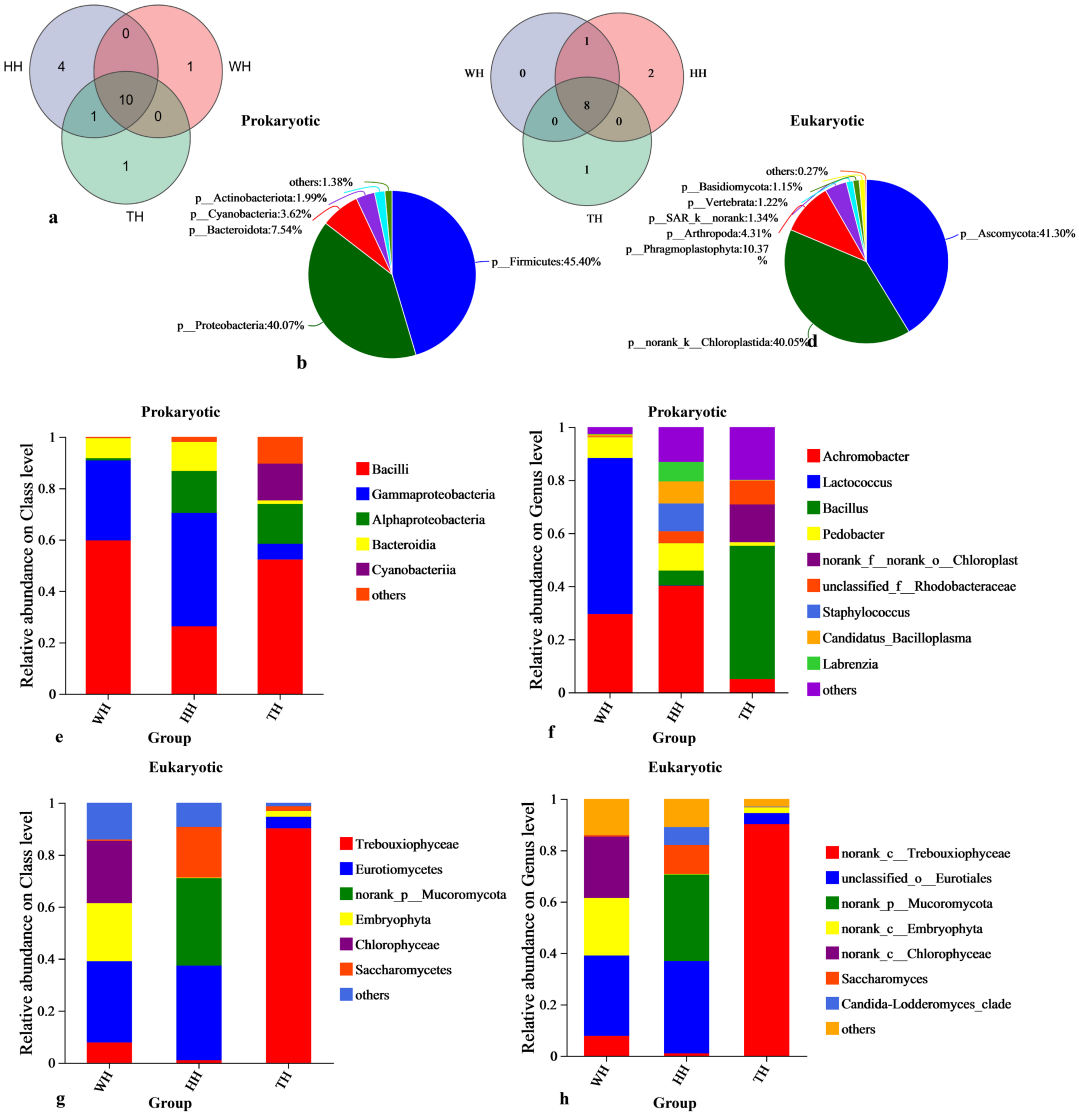
Microbial community composition in shrimp hepatopancreas. **(a,c)** Venn diagrams show unique and shared prokaryotic and eukaryotic phyla across WH, HH, and TH groups. **(b,d)** Pie charts display the relative abundance of core prokaryotic and eukaryotic phyla. Bar charts depict the relative abundance of the most abundant **(e,f)** prokaryotic and **(g,h)** eukaryotic genera at the sample level. The *x*-axis represents the sample groups (HH, TH, WH), and the *y*-axis indicates the relative abundance (%) of prokaryotic or eukaryotic taxa. Colors distinguish between different taxa.

High-throughput 16S rRNA sequencing revealed that probiotic supplementation markedly shaped the hepatopancreatic microbiome. Overall, 21 bacterial phyla were detected across all groups, with HH exhibiting the highest taxonomic richness (15 phyla), followed by TH (12 phyla) and WH (11 phyla) (Figure 3a). Despite group-specific differences, 10 core phyla, including *Bacillota*, *Pseudomonadota*, *Bacteroidota*, *Cyanobacteriota*, and *Actinomycetota* dominated all samples, accounting for over 98% of total bacterial abundance (Figure 3b).

**Figure 4:**
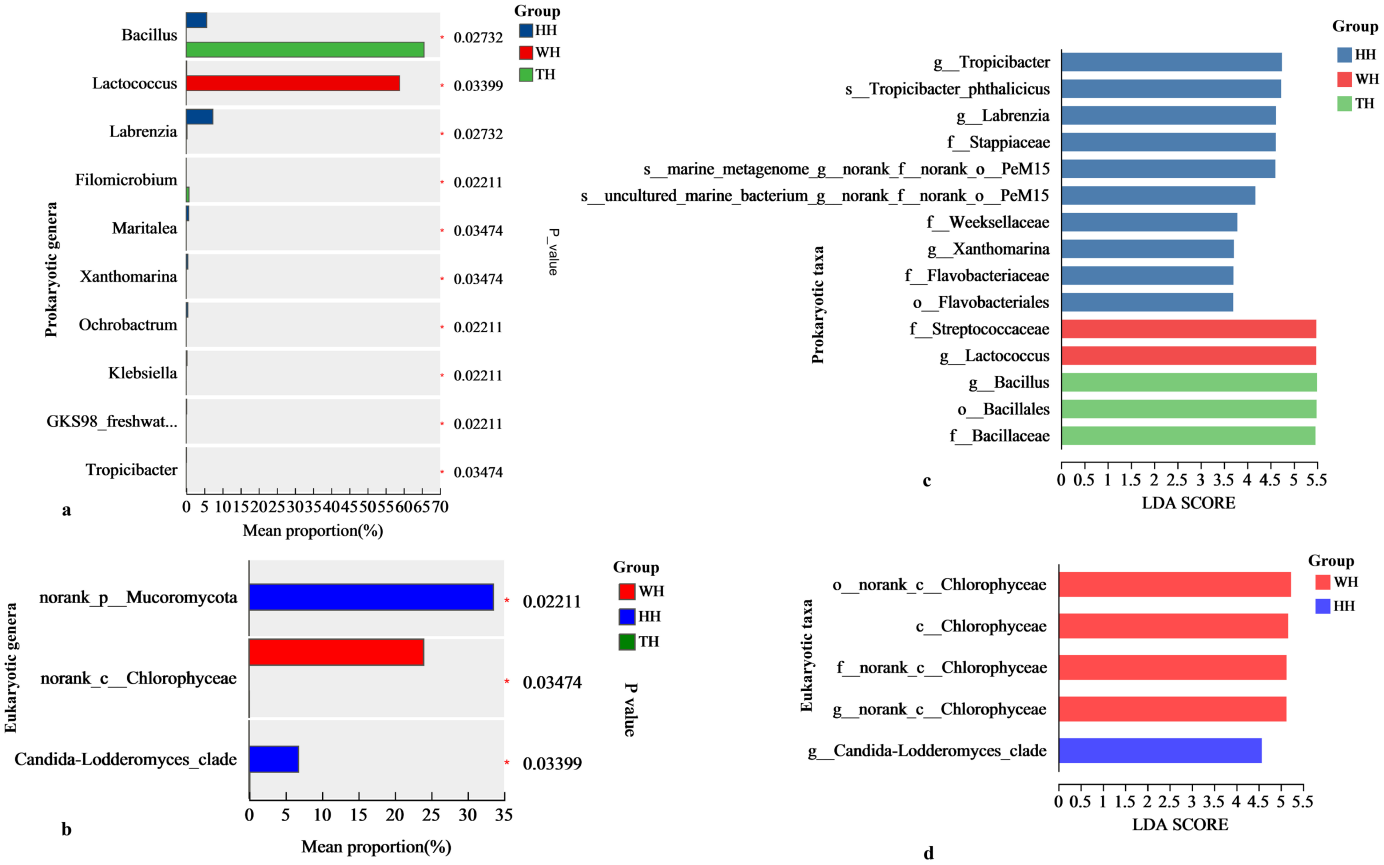
Differential abundance analysis of hepatopancreas microbiota. **(a, b)** Kruskal–Wallis H‑test bar plots showing prokaryotic **(a)** and eukaryotic **(b)** genera with significant differences among groups. The *x*‑axis shows mean relative abundance (%), and the *y*‑axis lists microbial taxa. Significance threshold: ^*^p ≤ 0.05. **(c, d)** LEfSe analysis highlighting differentially abundant prokaryotic **(c)** and eukaryotic **(d)** taxa. The *x*‑axis displays LDA scores (log_₁₀_), and the y‑axis shows discriminative taxa. Colors represent groups: HH (blue), WH (red), TH (green).

At the class and genus levels (Figures 3e,f), HH consistently exhibited the richest diversity, harboring 33 classes and 143 genera, compared with 134 genera in TH and 57 in WH. HH was characterized by an enrichment of *Alphaproteobacteria* and *Gammaproteobacteria*, whereas TH was dominated by *Bacilli* and *Cyanobacteriia*, and WH was dominated by *Bacilli* and *Gammaproteobacteria* (Figure 3e). Key dominant genera across all groups included *Bacillus*, *Lactococcus*, *Achromobacter*, and *Pedobacter*, but their relative abundances shifted dramatically depending on the probiotic treatment. Notably, HH favored potentially beneficial taxa such as *Achromobacter* and *Pedobacter*, while TH was enriched in *Bacillus*, and WH was dominated by *Lactococcus* (Figures 3f, 4a).

LEfSe analysis at the taxonomic level revealed strain-specific patterns of microbial enrichment (Figure 4c). The HH group exhibited selective enrichment of several taxa, including beneficial members such as *g__Labrenzia* and *g__Tropicibacter* (both belonging to *c__Alphaproteobacteria*), as well as *g__Xanthomarina* (within *f__Flavobacteriaceae*), which are associated with nutrient cycling and biofilm formation. In contrast, enrichment in the TH group was predominantly confined to *Bacillus*-related taxa. The WH group showed enrichment of *g__Lactococcus* and *f__Streptococcaceae*.

**Figure 5:**
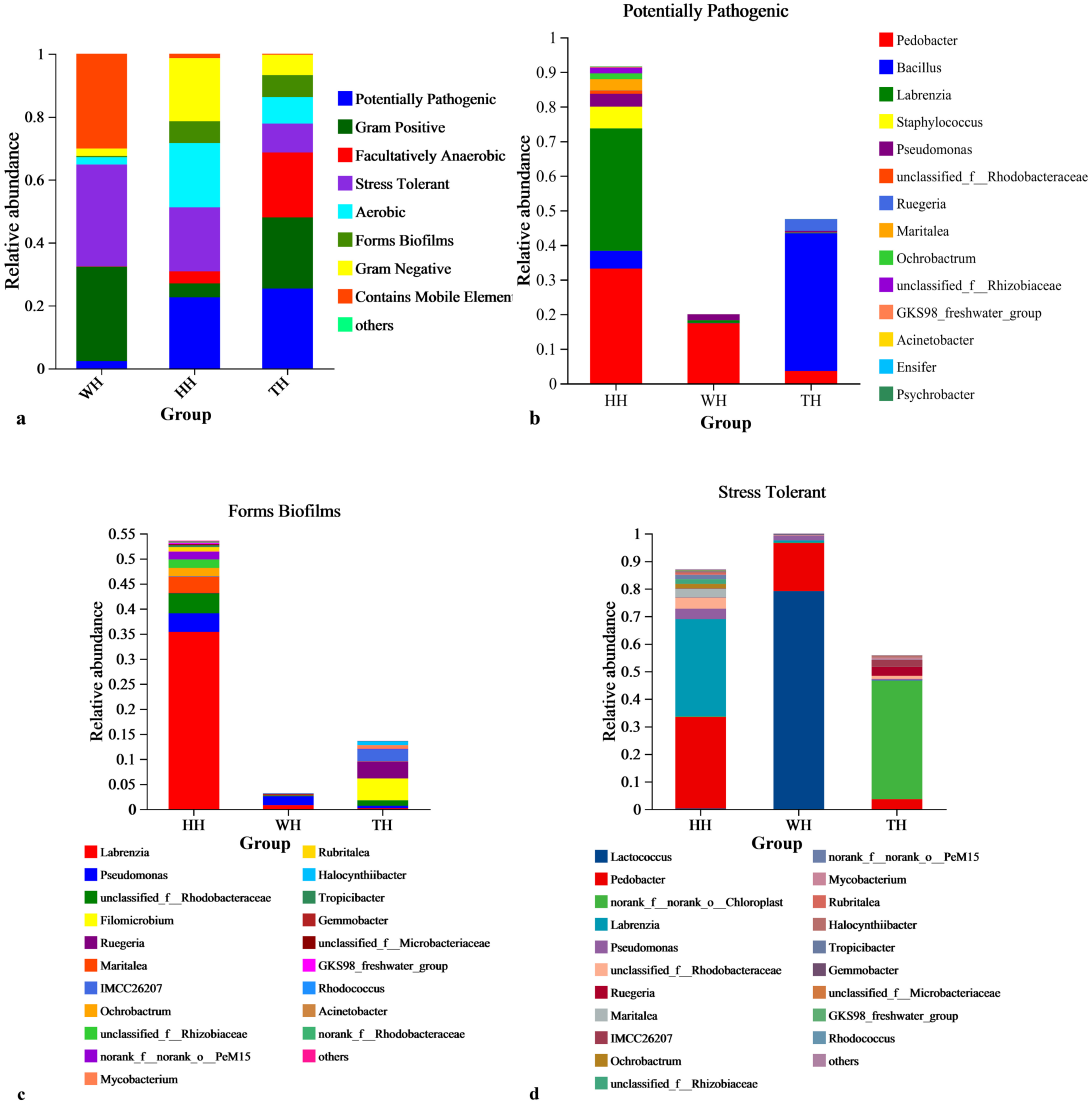
BugBase phenotype prediction of the hepatopancreas prokaryotic community. **(a)** Relative abundance of predicted phenotypic categories across sample groups (HH, TH, WH). The *y*‑axis shows relative abundance (%) of stress‑tolerant, potentially pathogenic, biofilm‑forming, Gram‑negative, Gram‑positive, aerobic, anaerobic, facultatively anaerobic bacteria, and taxa with mobile elements. **(b–d)** Genus‑level profiles of **(b)** potentially pathogenic, **(c)** biofilm‑forming, and **(d)** stress‑tolerant phenotypes. Relative abundance (%) of taxa is shown on the *y*‑axis, with sample groups on the *x*‑axis. Each color gradient corresponds to a specific genus in sub-figures **b–d**.

Functional inference using BugBase indicated that both HH and TH increased the abundance of biofilm-forming genera compared with WH, with HH particularly enhancing *Rhodobacteraceae*-associated biofilm formers (Figures 5a,c). Potentially opportunistic pathogens were most prevalent in TH (25%) (Figures 5a,b), consistent with KEGG analysis showing higher abundance of antimicrobial and secondary metabolite biosynthesis proteins, including surfactin, fengycin, and microcystin synthetase (Supplementary Table 11). In contrast, stress-tolerant bacteria were most abundant in WH (29%) (Figures 5a,d), which also showed higher levels of virulence-factor- and toxin-related proteins (Supplementary Table 11). KEGG functional annotation further revealed probiotic-dependent differences in hepatopancreatic functions (Supplementary Tables 11–15), with HH enriched in immune/virulence-related proteins, AMP synthesis, xenobiotic metabolism, tissue-repair pathways, and nitrogen- and sulfur-cycling processes, whereas TH showed stronger enrichment of proteolysis, amino-acid, lipid, and energy-metabolism pathways.

#### 3.4.3 Eukaryotic community composition in the hepatopancreas

High-throughput 18S rRNA sequencing demonstrated that probiotic supplementation altered the hepatopancreatic eukaryotic microbiome. The total number of enriched eukaryotic taxa at the phylum level was highest in the HH group (12), followed by TH (9) and WH (8) (Figure 3c). The hepatopancreatic eukaryotic community was primarily dominated by the fungal phyla *Ascomycota* and *Basidiomycota*, together with the animal phylum *Arthropoda*, while plant-related lineages including *norank_k__Chloroplastida* and *Phragmoplastophyta* were also detected (Figure 3d).

At the class and genus levels, HH showed the greatest diversity, comprising 18 classes and 26 genera, compared with 16 classes/18 genera in WH and 14 classes/14 genera in TH (Figures 3g,h). The dominant classes included *Eurotiomycetes*, *Trebouxiophyceae*, *Chlorophyceae*, and *Saccharomycetes* (Figure 3g). Genera with ≥5% relative abundance belonged to both fungal and algal lineages, indicating that the hepatopancreatic eukaryome consisted primarily of these core groups (Figure 3h).

Significance testing revealed that the fungal taxa *norank_p__Mucoromycota* and *Candida–Lodderomyces_clade* were significantly enriched in HH, whereas the green-algal taxon *norank_c__Chlorophyceae* was significantly higher in WH (Figure 4b). LEfSe analysis showed a consistent pattern (Figure 4d), with WH enriched in *Chlorophyceae*-associated taxa and HH selectively enriched in *Candida–Lodderomyces_clade*. No taxa were significantly enriched in TH, indicating that mixed-strain probiotic supplementation exerted minimal selective pressure on the eukaryotic community.

#### 3.4.4 Significant interspecies correlations between dominant prokaryotic and eukaryotic genera

Spearman’s correlation analysis revealed robust interkingdom associations between the top 20 prokaryotic and eukaryotic genera (Supplementary Figure 4). Several strong positive correlations (*r* > 0.7, *p* < 0.01) were detected between biofilm-associated bacteria, *unclassified_f_Rhodobacteraceae* and *Maritalea*, and members of the *Candida–Lodderomyces*_*clade* (Figure 5c; Supplementary Figure 4), suggesting potential co-occurrence or cooperative interactions within biofilm-enriched niches. Contrasting interaction patterns were also observed. *Bacillus* exhibited significant negative correlations (*r* < −0.6, *p* < 0.05) with *unclassified_o_Capnodiales* and *norank_p_SAR*, whereas *Lactococcus* demonstrated opposite association trends with these eukaryotic groups (Supplementary Figure 4), highlighting taxon-specific ecological relationships within the microbial network. Ecologically relevant antagonistic interactions were identified for the algal lineage *norank_c_Trebouxiophyceae*, which was negatively correlated with the opportunistic genus *Pedobacter* (*r* = −0.58, *p* < 0.05; Figure 5b; Supplementary Figure 4), yet positively associated with the biofilm-forming *Filomicrobium* (*r* = 0.65, *p* < 0.01; Figure 5c; Supplementary Figure 4). Moreover, *norank_p_Mucoromycota* occupied a central position within the interaction network, displaying positive correlations with beneficial biofilm-associated genera (*Labrenzia* and *Maritalea*; *r* > 0.6, *p* < 0.01) as well as with *Staphylococcus* (*r* = 0.52, *p* < 0.05; Figures 5b,c; Supplementary Figure 4). Collectively, these findings indicate a complex web of cooperative and antagonistic interspecies relationships that may contribute to microbiome structuring and ecological stability under probiotic modulation.

#### 3.4.5 Probiotic modulation of microbiome-mineral/metal interactions in the hepatopancreas

**Figure 6:**
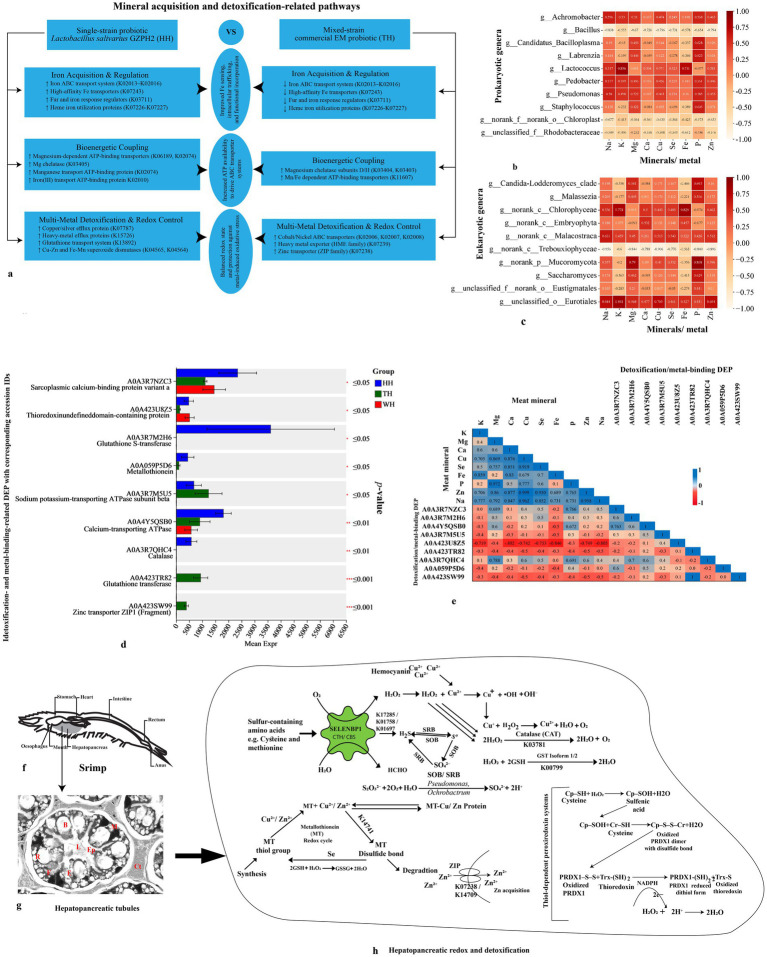
Strain‑specific probiotic modulation of mineral acquisition, microbiome–mineral interactions, detoxification pathways, and redox homeostasis in the hepatopancreas of *Litopenaeus vannamei*. (a) Conceptual model illustrating differential mineral acquisition and detoxification-related pathways enrichment between the single‑strain probiotic *Lactobacillus salivarius* GZPH2 (HH) and the mixed‑strain EM probiotic (TH), highlighting strain‑specific regulation of iron acquisition, bioenergetic coupling, and multi‑metal detoxification/redox control. **(b,c)** Correlation heatmaps showing associations between dominant prokaryotic **(b)** and eukaryotic **(c)** genera and hepatopancreatic mineral profiles. Color gradients represent correlation strength (red, positive; orange, negative). Relative taxonomic abundance (%) is plotted on the *y*‑axis, and mineral concentrations on the x‑axis. **(d)** Relative expression of significant detoxification‑ and metal‑binding‑related differentially expressed proteins (DEPs) across groups (HH, blue; TH, green; WH, red). Significance: ^*^p ≤ 0.05, ^**^p ≤ 0.01, and ^***^p ≤ 0.001. Metal‑binding DEP names with corresponding accession IDs are shown on the *y*‑axis, and mean DEP expression levels on the *x*‑axis. **(e)** Triangular correlation matrix heatmap illustrating Pearson correlation coefficients between hepatopancreatic mineral concentrations and expression profiles of detoxification/metal‑binding DEPs. **(f)** Anatomical location of the hepatopancreas and **(g)** representative histological micrograph showing cellular organization of hepatopancreatic tubules (E, E-cell; R, R-cell; F, F-cell; B, B-cell; M, M-cell; Ep, epithelium; L, lumen; Ct, connective tissue). **(h)** Proposed mechanistic model of hepatopancreatic redox regulation and metal detoxification, illustrating coordinated roles of hemocyanin-mediated copper redox cycling, metallothionein metal sequestration, glutathione-dependent detoxification, catalase activity, thioredoxin–peroxiredoxin (PRDX1) cycling, selenium-binding protein (SELENBP1) involvement, and ZIP-mediated zinc uptake.

Probiotic supplementation restructured the gut microbiome, establishing a mineral–microbe association network with distinct, taxon-specific architectures (Figures 6b,c). *Achromobacter*, *Labrenzia*, *Pseudomonas*, *norank_p__Mucoromycota*, *unclassified_o__Eurotiales*, and *Saccharomyces* were positively associated with hepatopancreatic Mg and P, while *Lactococcus* and *norank_c__Chlorophyceae* correlated with K and Fe. Conversely, *Bacillus* and *Trebouxiophyceae* taxa inversely correlated with Na, Ca, Se, Cu, and Zn, suggesting taxon-specific roles as net mineral sinks. Trace metals elicited opposing microbial signatures: u*nclassified_f__Rhodobacteraceae* correlated negatively with Cu and Zn, whereas *unclassified_o__Eurotiales* correlated positively, revealing divergent metal ecophysiology within the community.

Functional reconstruction of KEGG Ortholog (KO) pathways involved in metal ion transport and homeostasis, detoxification predicted by PICRUSt2 from 16S rRNA profiles, revealed clear probiotic-specific restructuring of microbial metal networks (Figure 6a and Supplementary Table 6). HH exhibited the strongest enrichment of iron acquisition systems, including coordinated upregulation of iron-complex ABC transporters, high-affinity Fe transporters, iron-responsive regulators, and heme utilization proteins. This concerted pattern indicates activation of an integrated iron-scavenging and regulatory framework rather than isolated transporter induction. Concurrent enrichment of magnesium-linked ATP-dependent transport modules supports the energetic demands of intensified metal uptake. Detoxification capacity was simultaneously reinforced through elevated heavy metal efflux systems and increased abundance of Fe–Mn and Cu–Zn superoxide dismutases, suggesting tight coupling between iron import and oxidative stress buffering. In contrast, TH showed partial activation of iron uptake pathways relative to WH but remained markedly lower than HH, while preferentially maintaining cobalt/nickel transporters and selected efflux systems, consistent with a detoxification-biased metal strategy.

### 3.5 Proteomics analysis

#### 3.5.1 Probiotic strain-specific remodeling of the hepatopancreatic proteome

High-resolution 4D-DIA proteomics quantified 353 confidently annotated proteins from 3,018 peptides (Supplementary Table 7), enabling direct comparison between HH and TH probiotic treatments. HH exhibited a higher overall proteome coverage (304 proteins) than TH (291 proteins), indicating broader proteomic activation under the single-strain formulation (Supplementary Figure 5). Direct HH vs. TH comparison identified 113 differentially expressed proteins (DEPs), with a near-balanced distribution (62 higher in HH; 51 higher in TH), underscoring substantial functional divergence between probiotic strategies despite shared probiotic exposure (Supplementary Figure 5c). Notably, the magnitude and functional orientation of these differences were biologically distinct rather than merely quantitative.

**Figure 7:**
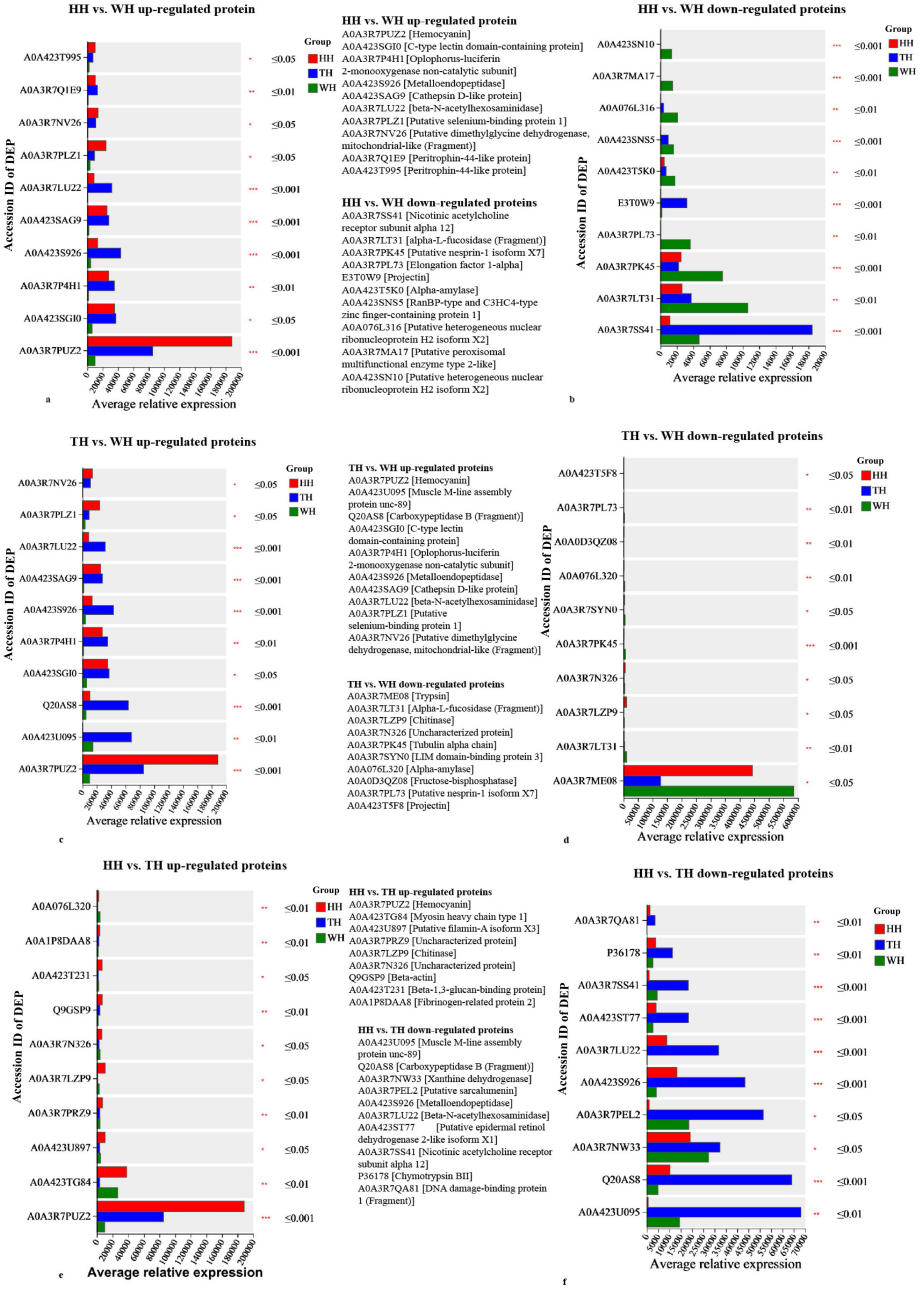
Expression profiles of significant differentially expressed proteins (DEPs). Bar charts show the mean relative expression of **(a,b)** upregulated and downregulated DEPs in HH vs. WH, **(c,d)** TH vs. WH, and **(e,f)** HH vs. TH comparisons. DEPs accession IDs are on the *y*-axis; mean relative expression is on the *x*-axis. Groups are color-coded: HH (red), TH (blue), WH (green). Significance levels: ^*^p ≤ 0.05, ^**^p ≤ 0.01, and ^***^p ≤ 0.001.

Functionally, HH preferentially enriched oxygen transport and redox-associated proteins, with hemocyanin (Figures 7a,c,e) and selenium-binding protein 1 (SELENBP; Figure 7a) emerging as key discriminators, highlighting enhanced oxygen delivery and selenium-linked antioxidant regulation. In contrast, TH selectively upregulated immune-effector and barrier-related proteins, including C-type lectin domain-containing protein (CTLD), beta-N-acetylhexosaminidase (HEXA_B), cathepsin D-like protein (CTSDP), and peritrophin-44-like protein (PT44), indicating reinforced pathogen recognition and lysosomal processing capacity (Figures 7a,c). Notably, opposing expression patterns were observed for neuromuscular-related proteins, where nicotinic acetylcholine receptor subunit alpha 12 and muscle unc-89 were significantly downregulated in HH but upregulated in TH relative to WH (Figures 7b,f), further underscoring mechanistic specialization.

#### 3.5.2 Probiotic supplementation alters digestive enzyme profiles

**Figure 8:**
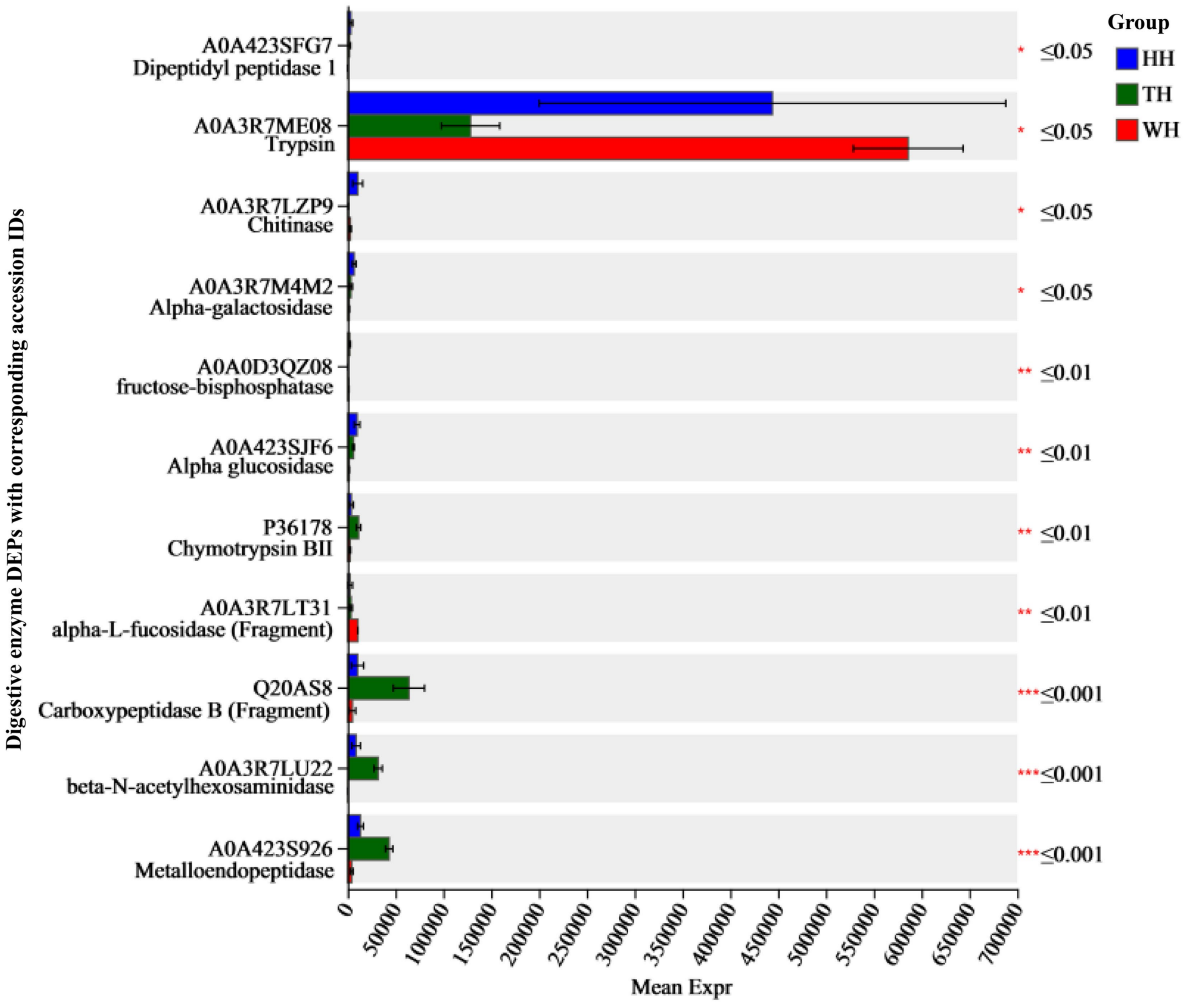
Expression of differentially expressed digestive enzymes. Bar charts show the mean relative expression of significant digestive enzyme DEPs across the HH, TH, and WH groups. The *y*‑axis lists DEP names with corresponding accession IDs; the *x*‑axis shows mean relative expression levels. Groups are color-coded: HH (blue), TH (green), WH (red). Significance levels: ^*^p ≤ 0.05, ^**^p ≤ 0.01, and ^***^p ≤ 0.001.

Proteomic analysis demonstrated that different probiotics distinctly modulated the production of digestive enzymes in the shrimp hepatopancreas under tropical semi-intensive rearing conditions (Figure 8). Both HH and TH exhibited significantly reduced trypsin expression compared to WH, with higher levels in TH than in HH (TH > HH; *p* < 0.05). This decrease was accompanied by a coordinated upregulation of alternative proteolytic enzymes, including carboxypeptidase B, metalloendopeptidase, chymotrypsin BII, and dipeptidyl peptidase 1. Carbohydrate metabolism was significantly enhanced in the probiotic groups, as evidenced by increased expression of alpha-glucosidase and alpha-galactosidase A, following the trend HH > TH > WH (Figure 8; Supplementary Figures 7a,c,e). Metabolic specialization was further reflected by elevated fructose-bisphosphatase (FBPase) in HH, whereas α-L-fucosidase was more abundant in WH compared to the probiotic groups. Chitinolytic enzymes also showed probiotic-specific regulation, with chitinase predominantly upregulated in the HH and beta-N-acetylhexosaminidase (HEXA_B) enriched in the TH.

#### 3.5.3 Proteomic evidence of probiotic-induced remodeling of mineral homeostasis, redox regulation, and immune pathways in the hepatopancreas

**Figure 9:**
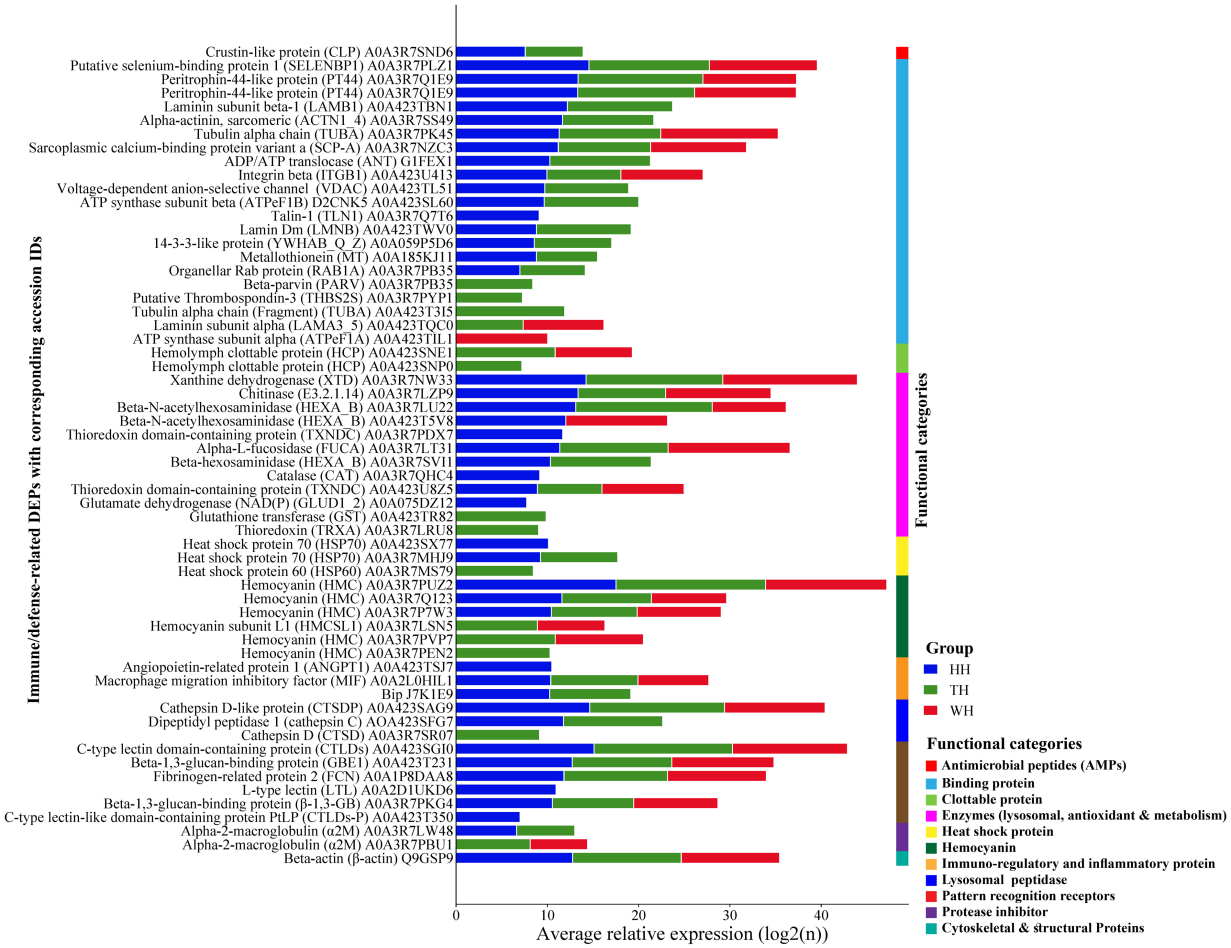
Expression profiles of immune‑ and stress‑related differentially expressed proteins (DEPs). Bar charts display the mean relative expression of immune‑ and stress‑associated DEPs across the HH, TH, and WH groups. Proteins are organized into functional categories: antimicrobial peptides, binding proteins, clottable proteins, enzymes, heat shock proteins, hemocyanin, immunoregulation and inflammation, lysosomal peptidases, pattern‑recognition receptors, protease inhibitors, and cytoskeletal/structural proteins, each represented by distinct colors within the figure. The *y*‑axis lists DEP names with corresponding accession IDs, while the *x*‑axis shows mean relative expression levels. Group colors: HH (blue), TH (green), WH (red).

Proteomic profiling revealed pronounced probiotic-driven remodeling of mineral transport, metal detoxification, redox regulation, and immune-related proteins in the hepatopancreas (Figures 6d,h, 9; Supplementary Figure 6; Supplementary Tables 8–10). Compared with the control (WH), both probiotic treatments (HH and TH) significantly altered the abundance of ion-regulatory, metal-binding, antioxidant, and immune-associated proteins, with clear strain-specific patterns (Supplementary Tables 8, 9).

HH exhibited a strong Ca^2+^-regulatory signature characterized by enrichment of Ca^2+^-transporting ATPase SERCA (ATP2A), calmodulin, sarcoplasmic Ca^2+^-binding protein (SCP-A), and voltage-dependent anion channel (VDAC2), indicating enhanced endoplasmic reticulum sequestration and mitochondrial Ca^2+^ flux control (Figures 6d,h and Supplementary Table 8). Na^+^/K^+^-ATPase subunits also differed between treatments, with ATP1A more abundant in HH and ATP1B enriched in TH. Zinc transporter ZIP1 was detected only in TH. Metallothionein (MT4) and selenium-binding protein (SELENBP1) were markedly higher in HH, whereas TH showed stronger induction of peroxiredoxin isoforms (PRDX1, PRDX3), glutathione S-transferase (GST)2, and epoxide hydrolase (EPHX1) (Figure 6h and Supplementary Table 9). Catalase (CAT) and sulfur-metabolism enzymes cystathionine gamma-lyase (CTH) and cystathionine beta-synthase (CBS) were detected primarily in HH, indicating treatment-specific redox and detoxification programming (Figure 6h and Supplementary Table 9). Correlation analysis showed coordinated mineral–protein networks involving Se, Ca, P, Fe, Cu, and Zn, with strong associations among CAT, GST, Ca^2+^-ATPase, metallothionein, and SCP-A (Figure 6e).

KEGG pathway analysis identified 60 immune-related differentially expressed proteins (DEPs), with both probiotic treatments showing higher abundance than the control (Figure 9 and Supplementary Table 10). TH exhibited slightly more immune-related DEPs (50) than HH (45). The antimicrobial peptide crustin-like protein (CLP) was detected only in HH and TH, with higher expression in HH. Chitinase and β-N-acetylhexosaminidase were upregulated in HH but reduced or absent in TH. HH uniquely expressed angiopoietin-related protein, L-type lectin, C-type lectin-like PtLP, talin-1, and heat shock protein 70 (HSP70), whereas hemolymph clottable protein, beta-parvin, HSP60, and several hemocyanin isoforms were detected only in TH (Figures 7, 9 and Supplementary Table 10). Among hemocyanin-related DEPs, DEP A0A3R7PUZ2 showed higher abundance in HH than in TH (Figures 7a,c,e).

KEGG functional annotation (Supplementary Table 10) indicated that these immune-related DEPs participated in multiple pathways. Hemocyanin-associated proteins were linked to tyrosine metabolism, glycerophospholipid metabolism, and melanogenesis, whereas chitinase and β-N-acetylhexosaminidase were involved in lysosomal function and glycosphingolipid biosynthesis (Supplementary Figures 8a,c). HSP70 was associated with MAPK signaling, antigen processing and presentation, and longevity-regulating pathways, while talin-1 and beta-parvin were connected to the PI3K–Akt signaling pathway.

KEGG enrichment analysis further revealed distinct pathway activation between treatments (Supplementary Figure 6). HH showed significant enrichment of leukocyte transendothelial migration, NET formation, focal adhesion, regulation of actin cytoskeleton, and Rap1 signaling (*p* < 0.01), whereas TH exhibited stronger enrichment of antigen processing and presentation, apoptosis, and longevity-regulating pathways (*p* < 0.001). Both probiotic groups also showed significant enrichment of thermogenesis, apoptosis, and environmental adaptation pathways (*p* < 0.001), confirming broad activation of hepatopancreatic immune and metabolic functions (Supplementary Figure 6).

#### 3.5.4 Probiotic modulation of microbiome–immune protein interactions in the hepatopancreas

**Figure 10:**
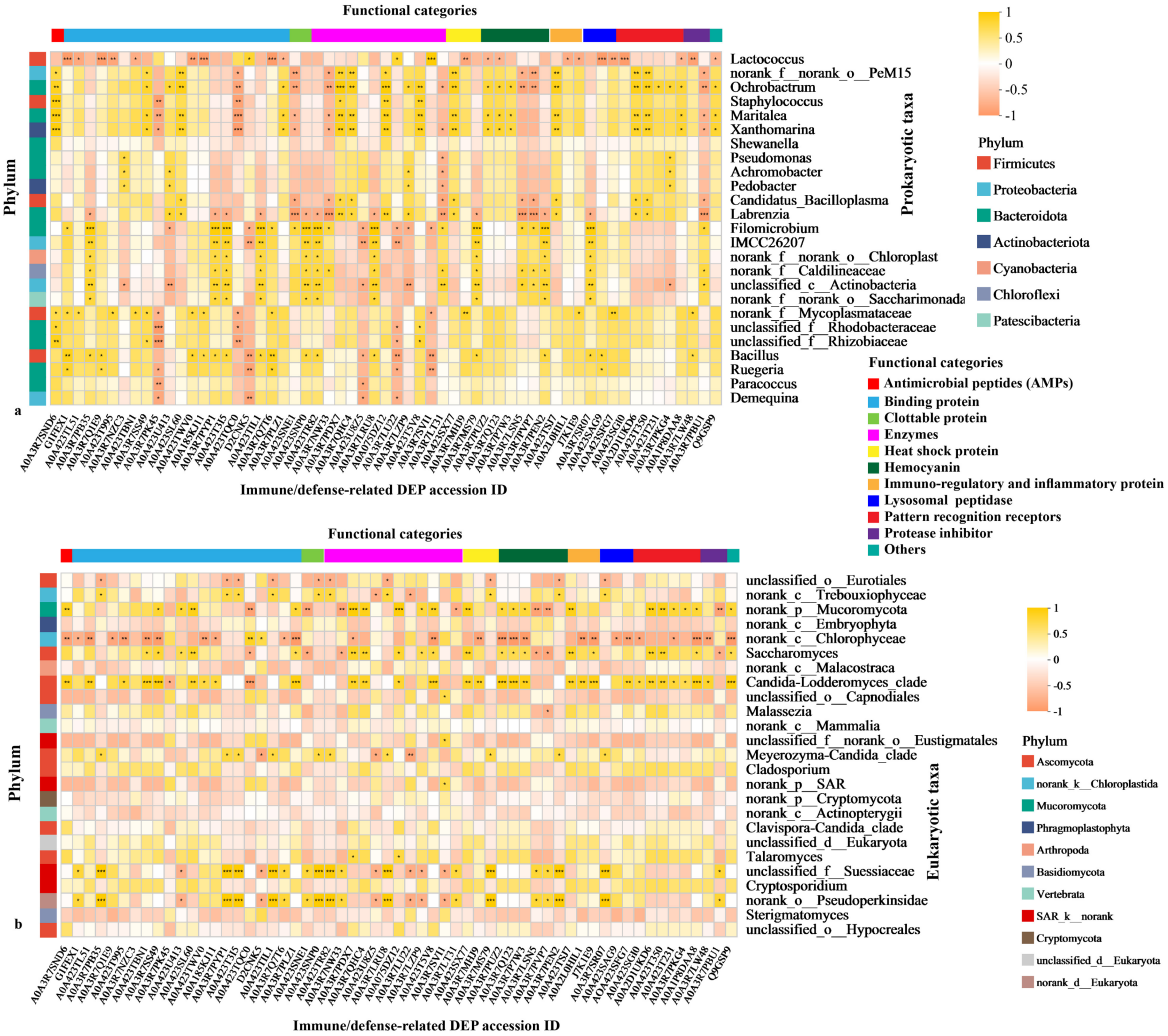
Cross-domain correlations between microbiota and host functional proteins. Spearman correlation heatmaps between the top 25 **(a)** prokaryotic and **(b)** eukaryotic genera and 60 immune/defense-related DEPs. The *x*‑axis lists DEP accession IDs, while the *y*‑axis lists microbial taxa. Correlation strength is encoded by color, and statistical significance is indicated by asterisks (^*^p ≤ 0.05, ^**^p ≤ 0.01, ^***^p ≤ 0.001). The correlation coefficient (*r*) is classified as follows: a very strong positive correlation is indicated by 1.0 ≥ *r* > 0.75, a strong positive correlation by 0.75 ≥ *r* > 0.5, a moderate positive correlation by 0.50 ≥ *r* > 0.25, a weak positive correlation by 0.25 ≥ *r* > 0, no correlation by *r* = 0, a weak negative correlation by 0.0 > *r* ≥ −0.25, a moderate negative correlation by −0.25 > *r* ≥ −0.50, a strong negative correlation by −0.50 > *r* ≥ −0.75, and a very strong negative correlation by −0.75 > *r* ≥ −1.0.

Spearman’s correlation analysis identified structured associations between the top 25 prokaryotic/eukaryotic genera and 60 immune- and stress-related DEPs in the shrimp hepatopancreas (Figure 10). Distinct taxon–protein interaction patterns were observed among WH, HH, and TH groups. Although *Bacillota* predominated in WH and TH, *Lactococcus* was enriched in WH and negatively correlated (*r* ≥ 0.5, *p* < 0.001) with immune-related DEPs organellar rab protein RAB1A, lamin Dm (LMNB), PT44, ADP/ATP translocase (ANT), cathepsin D-like protein (CTSDP), whereas *Bacillus* in TH showed positive correlations with the same proteins (*r* ≥ 0.5, *p* < 0.05) (Figure 10a). BugBase analysis indicated enrichment of stress-tolerant and potentially pathogenic taxa (*Staphylococcus*, *Maritalea*, *unclassified_f_Rhizobiaceae* and *unclassified_f_Rhodobacteraceae*) in HH/TH. Antimicrobial peptides (e.g., CLP), exclusive to probiotic groups, were strongly positively correlated with these genera (*r* ≥ 0.5, *p* < 0.05–0.001) (Figures 5, 10a). Hemocyanin correlated positively with aerobic taxa (*Ochrobactrum*, *Maritalea*, *Xanthomarina*) and negatively with anaerobes (*Lactococcus*, *Candidatus_Bacilloplasma*).

Eukaryotic taxa showed similar trends, with *norank_p_Mucoromycota* and *Candida-Lodderomyces_clade* positively associated with immune/stress DEPs, whereas ATP synthase subunit alpha (ATPeF1A) and fibrinogen-related protein 2 (FCN) were negatively correlated with *norank_c_Chlorophyceae* (Figure 10b). Overall, probiotics reshaped microbiome–immune protein interaction networks in the hepatopancreas.

## 4 Discussion

The hepatopancreas of penaeid shrimp serves as the principal organ responsible for digestion, nutrient assimilation, mineral storage, immune defense, and stress regulation, and therefore plays a central role in determining growth performance and physiological homeostasis. In the present study, an integrated multi-omics approach combining histological evaluation, microbiome profiling, mineral analysis, and hepatopancreatic proteomics demonstrated that probiotic supplementation remodels this organ at multiple biological levels, leading to improved growth, feed efficiency, and survival in *L. vannamei* (Table 1). Notably, the single-strain probiotic (HH) and the mixed-strain probiotic (TH) did not act redundantly, but instead established distinct functional states within the hepatopancreas, indicating that probiotic effects arise from specific physiological reprogramming rather than from a generalized growth-promoting response. Although both treatments increased biomass by approximately ~26–27% compared with the control (Table 1), the underlying mechanisms differed substantially, suggesting that comparable production outcomes can be achieved through alternative metabolic, microbial, and immunological strategies. The following sections therefore examine how strain-specific probiotics differentially influence hepatopancreatic cellular organization, microbiome architecture, mineral–protein coupling, metabolic regulation, and immune defense pathways.

### 4.1 Strain-specific probiotic performance gains are linked to distinct hepatopancreatic functional remodeling

Improved performance in shrimp fed the single-strain probiotic HH and the mixed-strain probiotic TH was associated with clear differences in hepatopancreatic epithelial organization, indicating strain-specific functional remodeling of this organ (Figure 1 and Table 2). HH showed significantly higher proportions of embryonic cells (E-cells) and fibrillar cells (F-cells), whereas TH was dominated by resorptive cells (R-cells) and blister-like cells (B-cells). Because E-cells act as progenitors for all hepatopancreatic cell types, their abundance reflects active epithelial renewal, while F-cells are involved in protein synthesis, immune molecule production, and detoxification (Vogt, 2019; Zhu et al., 2022; Florea et al., 2026). Consistent with this role, HH showed higher levels of hemocyanin and lectin-type pathogen-recognition proteins, together with enrichment of heavy-metal detoxification and xenobiotic-metabolism pathways (Supplementary Tables 9, 14). The predominance of E- and F-cells therefore suggests accelerated epithelial regeneration and a shift toward a metabolically active and protective hepatopancreatic state (Ribeiro et al., 2014).

In contrast, TH maintained higher proportions of R- and B-cells, which are responsible for nutrient absorption, lipid and glycogen storage, and digestive enzyme secretion (Vogt, 2019; Zhu et al., 2022). This pattern agreed with KEGG and proteomic results showing stronger enrichment of lipid digestion, amino-acid metabolism, and digestive enzyme pathways in TH (Figure 8 and Supplementary Table 12), indicating that the mixed-strain probiotic favored a digestion-oriented functional state rather than rapid epithelial renewal.

These cellular differences were closely associated with microbiome and metabolic changes. HH increased microbial diversity and enriched prokaryotic and eukaryotic taxa (Figures 3–5), and functional annotation indicated higher glycolysis and fatty-acid synthesis, pathways required for ATP production and membrane formation during tissue repair (Supplementary Table 12). In contrast, TH showed higher abundance of proteins related to amino-acid supply and gluconeogenesis, suggesting increased biosynthetic and digestive activity. Similar metabolic shifts have been reported during hepatopancreatic regeneration, where glucose metabolism, lipid synthesis, and amino-acid utilization support cell proliferation and protection of hepatopancreatic tissue (Duan et al., 2024; Zhu et al., 2024; Duan L. et al., 2025; Duan D. et al., 2025; Duan Y. et al., 2025).

The regenerative phenotype in HH was further supported by higher abundance of calcium-regulatory proteins (Ca^2+^-ATPase, calmodulin, SCP-A) and significantly greater calcium accumulation in the hepatopancreas (Supplementary Table 8; Table 3). Calcium signaling is a key regulator of epithelial proliferation and differentiation and can mediate microbiome-driven transcriptional responses (Singh and Pandey, 2020; Zhou et al., 2024). These results suggest that the single-strain probiotic promoted a repair-oriented and metabolically active hepatopancreatic state, whereas the mixed-strain probiotic supported digestive and storage functions.

### 4.2 Single probiotic outperforms mixed consortium by reshaping microbiome structure toward balanced community architecture

Alpha- and beta-diversity analyses (Tables 4, 5) demonstrated that probiotic supplementation restructured the hepatopancreatic microbiome of *L. vannamei*, but the mode of community assembly differed markedly between treatments. Both probiotics increased richness and Shannon diversity relative to the control; however, the single-strain probiotic treatment primarily increased evenness, whereas the mixed consortium probiotic mainly expanded richness. This distinction indicates that the principal effect of the single probiotic was not simple diversification but reorganization of community architecture, resulting in a more equitable distribution of taxa that limits dominance of opportunistic groups. These findings support the ecological concept that microbiome stability depends more on balanced abundance structure than on total taxonomic richness, suggesting that probiotic efficacy is determined by how community structure is reshaped rather than by how many taxa are introduced (Wittebolle et al., 2009).

At the phylum level, both treatments preserved the core microbiota (*Bacillota*, *Pseudomonadota*, *Bacteroidota*, *Cyanobacteriota*, and *Actinomycetota*) (Figures 3a,b), consistent with previous reports in *L. vannamei* (Yang S. et al., 2018; Yang W. et al., 2018; Zeng et al., 2017). HH promoted enrichment of *Alphaproteobacteria* while constraining *Gammaproteobacteria* expansion, resulting in a more favorable G/A ratio for HH, TH, and WH of 44.06 ± 23.12%, 16.34 ± 14.98%, and 2.70; 6.09 ± 0.33%, 15.49 ± 12.36%, and 0.39; and 6.09 ± 0.33%, 0.84 ± 0.54%, and 7.25, respectively (Figure 3e), indicating a more balanced microbial profile in HH. Similar patterns have been reported in healthy shrimp microbiomes, where *Alphaproteobacteria* dominance is associated with balance microbiomes (Zhu et al., 2016), whereas *Gammaproteobacteria* expansion is frequently linked to stress, dysbiosis, or disease (Kitsanayanyong et al., 2025). These results suggest that the single probiotic reshaped the dominant phylum toward a health-associated internal structure rather than simply altering total abundance, reflecting regulation of community hierarchy rather than replacement of taxa.

Genus-level analysis further indicated that HH promoted formation of a cooperative and metabolically integrated microbial network. Single-strain probiotic supplementation selectively enriched several members of the family *Rhodobacteraceae*, including *Labrenzia*, *Maritalea*, *Ruegeria*, *Tropicibacter*, and *unclassified_f__Rhodobacteraceae* (Figures 3f, 5c). Functional annotation of 16S rRNA genomes revealed that HH showed higher representation of ecological processes such as carbohydrate degradation, aerobic denitrification, assimilatory nitrate reduction, ammonium assimilation, and sulfur oxidation (Supplementary Tables 13, 15), consistent with previous studies reporting similar functional roles for *Rhodobacteraceae*-dominated communities (Rajeev and Cho, 2024). These KEGG pathways are characteristic of metabolically cooperative microbiomes that support host homeostasis through efficient nutrient turnover and environmental buffering.

In contrast, the control group displayed features of dysbiosis, including higher abundance of *Lactococcus* (Figures 3f, 5b), genera frequently associated with stress conditions or opportunistic infections in aquatic animals (Xu et al., 2024; Paul et al., 2025). Functional annotation supported this interpretation, as WH showed increased abundance of virulence-factor and toxin-related genes (Supplementary Table 11), which are typically involved in microbial competition, host invasion, and pathogenicity (Billington et al., 2000; Jeong et al., 2023). Both probiotic treatments reduced these signatures, but HH produced the most even and cooperative community structure, consistent with restoration of microbiome equilibrium rather than competitive suppression. In contrast to HH, the TH consortium generated a functionally specialized but taxonomically dominated community in which *Bacillus* accounted for 65.61% of total abundance. This dominance coincided with enrichment of antimicrobial and secondary-metabolite KEGG pathways, including surfactin, fengycin, polyketide, and siderophore biosynthesis (Supplementary Table 11), consistent with the well-established capacity of *Bacillus* spp. to produce antimicrobial lipopeptides that suppress competing microbes and enhance host disease resistance (Lu et al., 2023; Qiao et al., 2024; Yan et al., 2026). TH also showed higher abundance of toxin-associated genes, including thiol-activated cytolysin, hemolysin III, microcystin, and immune-inhibitor proteins (Supplementary Table 11), indicating intensified microbial competition and defense activity (Ramarao and Sanchis, 2013; Saha et al., 2022; Moreira et al., 2025). This competitive functional profile corresponded with the higher digestive enzyme activity, growth performance, and metabolic capacity observed in TH (Table 1, Figure 8, Supplementary Figures 4c, 5c, and Supplementary Table 13), where amino-acid metabolism, lipid digestion, TCA cycle, and carbohydrate pathways were most enriched. Together, these results indicate that the consortium drove the microbiome toward a high-output functional state dominated by a few taxa, rather than toward a structurally balanced community.

Cross-kingdom analysis further highlighted differences in community organization. HH enriched *norank_p_Mucoromycota* and *unclassified_o_Eurotiales*, which positively correlated with HH-associated bacteria such as *Labrenzia* and *Maritalea* (Figure 5c; Supplementary Figure 4), suggesting coordinated bacterial–fungal interactions known to contribute to immune regulation and microbiome stability in aquatic hosts (Huang et al., 2024). In contrast, TH showed higher abundance of *norank_c__Trebouxiophyceae* within *Chloroplastida* (Figures 3g, 4d), indicating shifts in oxygen-related metabolic processes that may influence hemocyanin-mediated immune responses (Huang et al., 2020), but with reduced overall evenness, consistent with a dominance-driven community structure.

Collectively, these findings demonstrate that probiotic effectiveness is determined by the balance of microbiome architecture rather than by the dominance of a few taxa. The even, cooperative community promoted by GZPH2 enhances host resilience, underscoring its potential as a targeted probiotic strategy for sustaining hepatopancreatic health in shrimp aquaculture. In contrast, the mixed consortium primarily increased richness without achieving the same balance.

### 4.3 Mineral–hepatopancreatic microbiome–protein coupling defines probiotic-specific ion and redox strategies

The hepatopancreas of *L. vannamei* functions as the central hub of nutrient metabolism, mineral storage, and detoxification, thereby orchestrating growth performance and immune competence (Vogt, 2019). As the primary site of metal bioaccumulation in crustaceans, it integrates trace-element uptake with antioxidant buffering to prevent metal-induced oxidative injury (Heidarieh et al., 2013; Karam et al., 2023). In line with this physiological centrality, Table 3 and Figure 6 demonstrate that probiotic supplementation drives distinct restructuring of mineral transport, microbiome composition, and redox-regulatory proteins.

Mineral profiling (Table 3) revealed that HH accumulated higher Mg, Fe, Ca, and Se, whereas TH exhibited comparatively reduced mineral levels. These elements are directly linked to hepatopancreatic function: Mg sustains ATP-dependent enzymatic reactions and protein synthesis (Davis et al., 1992); Fe supports respiratory and immune oxidoreductases but can catalyze ROS formation when unchelated (Mu et al., 2021; Lin et al., 2025); Ca regulates epithelial renewal and digestive secretion (Pinto et al., 2015); and Se strengthens selenoprotein-driven antioxidant defense (Huang et al., 2022; Lee et al., 2025). Collectively, their coordinated regulation underpins metabolic turnover, digestive efficiency, and oxidative resilience.

Microbiome–mineral network analysis (Figures 6b,c) confirms that probiotic-induced restructuring of the hepatopancreatic microbial community directly modulates trace-element bioavailability (Matthews et al., 2023; Jyoti and Dey, 2025). This was particularly evident in the single probiotic group (HH), which exhibited an acquisition-intensive metabolic configuration. Enrichment of KEGG pathways related to ion acquisition and transport, specifically the upregulation of Fe- and Mg-transport modules (Figure 6a and Supplementary Table 6), supported this finding and was corroborated by corresponding increases in Fe and Mg concentrations (Table 3).

Proteomic data further revealed higher abundance of ATP-dependent ion pumps (Na^+^/K^+^-ATPase, Ca^2+^-ATPase), MT4, and SELENBP1 in HH (Figures 6d,h and Supplementary Tables 8, 9). These ion-transport programs were tightly coupled with antioxidant effectors, including Cu–Zn superoxide dismutase, catalase, and GST1 (Koner et al., 2021; Zhou et al., 2022; Furukawa and Fujieda, 2025). Positive mineral–protein correlations (Figure 6e) indicate coordinated enhancement of metabolic flux and redox buffering, allowing elevated mineral uptake without triggering metal-driven oxidative injury. Sustained MT expression despite increased metal load underscores an adaptive homeostatic response that stabilizes intracellular metal pools while preventing ROS amplification (Chen et al., 2014; Malavolta and Pabis, 2022).

In contrast, the TH group exhibited a *Bacillus*-dominated microbiome (Figure 3f) that coincided with elevated ZIP expression (Supplementary Table 6) and reduced hepatopancreatic zinc concentrations (Table 3). Given that zinc is essential for *Bacillus* growth and metabolism (Yadav et al., 2023), its depletion likely reflects microbial competition for available Zn. The concurrent upregulation of the high-affinity zinc importer ZIP1 suggests a compensatory host response to zinc scarcity, consistent with metal-responsive transporter induction described in other systems (Xing et al., 2016). Proteomically, TH displayed a detoxification-oriented signature characterized by enrichment of ZIP1, GST2, epoxide hydrolase, and peroxiredoxins (PRDX1/3) (Figure 6h and Supplementary Table 9). This configuration indicates a moderated-uptake strategy coupled to enhanced peroxide management. The prominence of PRDX1/3 implies reliance on the thioredoxin–peroxiredoxin axis for precise intracellular H₂O₂ regulation, rather than catalase-dominated bulk detoxification (Neumann et al., 2009). Concurrently, attenuated Fe-transport and Ca^2+^-regulatory pathway enrichment (Figure 6a and Supplementary Table 6) supports a conservative ion-management state, limiting redox-active metal influx.

Together, these findings define two probiotic-specific immunometabolic states: HH promotes coordinated mineral acquisition with strong antioxidant buffering, whereas TH adopts a moderated-uptake, peroxide-regulated strategy. Mineral–microbiome–protein coupling thus emerges as a key determinant of hepatopancreatic homeostasis and oxidative balance in shrimp aquaculture.

### 4.4 Probiotics enhance shrimp metabolism through hepatopancreatic protein regulation and gut microbiota modulation

Probiotic supplementation reshaped hepatopancreatic metabolism through coordinated regulation of digestive enzymes and microbiome-derived metabolic functions, as supported by 16S rRNA functional KEGG prediction and 4D-DIA proteomic KEGG analysis (Figure 8 and Supplementary Tables 10, 13). Functional prediction of the gut microbiome revealed strain-specific metabolic programming (Supplementary Table 13). The mixed consortium (TH) markedly enriched proteolysis, amino-acid metabolism, lipid digestion, and TCA-cycle pathways, indicating high metabolic output driven primarily by protein utilization. In contrast, the single strain (HH) preferentially enhanced carbohydrate-degrading enzymes, glycolysis/gluconeogenesis, and energy-efficient pathways, suggesting improved metabolic balance and ATP efficiency under tropical culture conditions. These metabolic shifts coincided with improved growth performance and feed utilization in probiotic-fed shrimp (Table 1), consistent with previous reports showing that probiotics enhance shrimp growth by modulating gut microbiota, digestive enzyme activity, and host energy metabolism (Ringø et al., 2022; Yang et al., 2024).

Proteomic analysis confirmed distinct hepatopancreatic enzyme responses in *L. vannamei* (Figure 8 and Supplementary Table 10). With the exception of trypsin, several proteolytic enzymes including chymotrypsin BII, metalloendopeptidase, cathepsin D-like protein, dipeptidyl peptidase-1, carboxypeptidase A1, and carboxypeptidase were upregulated in both probiotic treatments, with stronger induction in TH, indicating enhanced proteolytic capacity. Both probiotics caused partial suppression of trypsin accompanied by compensatory chymotrypsin upregulation, moderate in HH but pronounced in TH, resembling developmental enzyme modulation reported in *Penaeus setiferus* (Lee and Lawrence, 1985). The increase of α2-macroglobulin isoforms in TH (Figure 8 and Supplementary Table 10), a known trypsin inhibitor (Harpel, 1973), suggests regulated proteolysis rather than uncontrolled enzyme activity.

Clear metabolic specialization was observed at the substrate level. TH treatment showed enrichment of aromatic amino-acid metabolism (tyrosine, phenylalanine, tryptophan), increased carboxypeptidase-B activity associated with arginine utilization (Hook and Loh, 1984), and activation of sphingolipid metabolism related to stress signaling (Figure 8, Supplementary Figures 7c, 8c, and Supplementary Table 10; Chung et al., 2000). These pathways indicate predominant amino-acid catabolism under high-protein dietary conditions, supporting rapid growth but with limited improvement in carbohydrate-based energy efficiency (Tobón-Cornejo et al., 2025).

In contrast, HH strongly promoted carbohydrate utilization. α-Glucosidase (1.60-fold) and α-galactosidase A (1.91-fold) were more highly induced than in TH (Figure 8), accompanied by enrichment of glycolysis/gluconeogenesis and glycosphingolipid biosynthesis pathways (Supplementary Figures 7a, 8a). Enhanced carbohydrate metabolism improves ATP yield efficiency, supports stress tolerance, and allows partial replacement of dietary protein with carbohydrates in feed formulations, which is considered a key strategy for sustainable shrimp production (Li et al., 2023; Bingqian et al., 2024).

Overall, both probiotics increased digestive and metabolic enzyme activity, but their functional outcomes differed. The mixed consortium mainly stimulated proteolytic and amino-acid metabolism, whereas the single strain preferentially enhanced carbohydrate utilization and energy efficiency. This metabolic profile suggests that *L. salivarius* GZPH2 is particularly suitable for tropical shrimp culture, where efficient energy use improves growth under environmental conditions (Singha et al., 2023) and reduces dependence on high-protein feed ingredients (Bingqian et al., 2024). Under the high-protein, low-carbohydrate diet used in this study (≥42% crude protein; ≥10.75% carbohydrate), probiotic-driven enhancement of carbohydrate metabolism may enable partial substitution of costly protein with carbohydrates, providing a practical strategy to improve both sustainability and production efficiency in shrimp aquaculture.

### 4.5 Immune defense mechanisms in shrimp hepatopancreas mediated by probiotic supplementation

Proteomic profiling clearly demonstrated that probiotic supplementation profoundly modulated immune defense mechanisms in the shrimp hepatopancreas, as evidenced by the significant differential expression of immune- and stress-associated proteins in both the single-strain (HH) and mixed-strain (TH) groups compared with the control (WH) (Figure 9 and Supplementary Table 10). Central immune effectors including hemocyanin, C-type lectin domain-containing proteins (CTLDs), crustacean-like proteins (CLPs), chitinase, hexosaminidase B (HEXA_B), peritrophin-44 (PT44), and β-actin, were consistently upregulated in probiotic-treated shrimp, following a general trend of HH > TH > WH (Figures 7, 9). Among these, hemocyanin exhibited the strongest induction, particularly in HH (*p* < 0.001; Figures 7a,c,e), and was significantly enriched in melanogenesis and tyrosine metabolism pathways (Supplementary Figures 8a,c). Beyond its classical role in oxygen transport, hemocyanin functions as a precursor of antimicrobial peptides and participates in activation of the phenoloxidase cascade, thereby contributing to broad-spectrum antimicrobial defense (Liu et al., 2007; Yang S. et al., 2018; Yang W. et al., 2018). Importantly, correlation analysis revealed that elevated hemocyanin expression was positively associated with beneficial aerobic taxa such as *Ochrobactrum*, *Maritalea*, *Xanthomarina*, and *Saccharomyces*, and negatively correlated with opportunistic anaerobes including *Lactococcus* and *Candidatus_Bacilloplasma* (Figures 10a,b), supporting its role in microbiome modulation and immune homeostasis (Zheng et al., 2021).

Distinct probiotic-specific regulatory patterns were observed for chitinolytic enzymes, suggesting divergent antifungal strategies between formulations. Chitinase was preferentially upregulated in HH, whereas HEXA_B expression predominated in TH (Figure 9 and Supplementary Table 10). Both enzymes are integral to chitin degradation and antifungal defense (Xi et al., 2015; Zhu et al., 2016), yet their differential induction implies that GZPH2 primarily enhances direct chitinase-mediated fungal lysis, while the mixed consortium favors downstream glycosidic hydrolysis via HEXA_B. Supporting this interpretation, chitinase levels in HH were strongly positively correlated with chitinase-producing fungal taxa, particularly the *Candida-Lodderomyces_*clade and *norank_p_Mucoromycota* (Figure 10b), indicating probiotic-driven modulation of fungal community dynamics. Concurrently, increased abundance of β-1,3-glucan-binding protein in HH further reinforced antifungal recognition and immune activation (Ma and Kanost, 2000; Lee et al., 2004).

Probiotic supplementation also strengthened epithelial integrity and cytoskeletal stability, key determinants of hepatopancreatic resilience under tropical heat stress. PT44 and β-actin were significantly more elevated in HH than TH (Figures 7, 9), consistent with their roles in maintaining peritrophic matrix structure and cytoskeletal organization during environmental stress (Wang et al., 2015). As ectothermic organisms, shrimp rely heavily on molecular chaperones for thermal adaptation; accordingly, probiotic treatment modulated heat shock responses, with TH significantly upregulating HSP60 and HH inducing distinct HSP70 isoforms (Figure 9) as well as thermogenesis pathway was enriched in both groups (Supplementary Figures 6j,k). Previously found that these chaperones are essential for proteostasis under thermal stress (Jeyachandran et al., 2023). Moreover, the upregulation of HEXA_B, CTSD, CTLDs, and RAB1A (Figure 9 and Supplementary Table 10) suggests activation of lysosomal and autophagic degradation pathways that remove damaged proteins and organelles, thereby maintaining cellular homeostasis during heat exposure (Gyurkovska et al., 2023; Calzoni et al., 2024).

KEGG pathway enrichment further underscored the immunomodulatory impact of probiotics, revealing significant overrepresentation of proteins involved in neutrophil extracellular trap (NET)-like formation, NOD-like receptor signaling, and cytoskeletal-mediated phagocytosis (Supplementary Figure 6 and Supplementary Table 10). NET-associated proteins, including PT44, SELENBP1, and β-actin were markedly elevated in probiotic groups (Supplementary Figures 6a,c), with SELENBP1 and β-actin more abundant in HH, whereas PT44 was more pronounced in TH, indicating formulation-specific modulation of extracellular antimicrobial mechanisms (Vorobjeva and Pinegin, 2014; Papayannopoulos, 2018). Enhanced NOD-like receptor signaling was suggested by increased PT44 expression (TH > HH) and exclusive detection of thioredoxin (TRXA) in TH (Supplementary Table 10), highlighting redox-regulated innate immune activation (Muri and Kopf, 2022). Additionally, proteins central to phagocytosis and intracellular trafficking, β-actin, integrin β1 (ITGB1), 14–3-3-like protein, and RAB1A, were differentially expressed (Supplementary Figures 6d,f), supporting improved cytoskeletal remodeling and pathogen clearance (Hynes, 2002; Pollard and Cooper, 2009). Activation of PI3K-Akt, MAPK, and Hippo pathways by probiotic-induced proteins such as SCP-A, VDAC, β-actin, and Talin-1 (detected exclusively in HH) further indicates coordinated regulation of immune signaling, stress adaptation, and cellular proliferation (Supplementary Figures 6c,g,h; Dupont et al., 2011). Notably, the absence of RAB1A and VDAC in the control group emphasizes the central role of probiotics in fortifying immune resilience (Bron et al., 2017).

Collectively, these findings demonstrate that while both probiotic formulations enhanced hepatopancreatic immune defense relative to the control, the single-strain GZPH2 (HH) elicited a more robust and coordinated activation of immune effectors, antifungal enzymes, cytoskeletal regulators, and stress-responsive proteins. This superior molecular response likely underpins improved pathogen resistance and thermal resilience, highlighting the strategic advantage of precision single-strain probiotic supplementation for optimizing shrimp health and supporting sustainable aquaculture under fluctuating tropical conditions.

## 5 Conclusion

This study demonstrates that probiotic supplementation improves growth of *L. vannamei* by remodeling hepatopancreatic function, but different probiotic strategies achieve this through distinct physiological states rather than a single universal mechanism. The single-strain probiotic (*L. sali*var*ius* GZPH2) induced a regeneration-oriented and metabolically efficient configuration characterized by epithelial renewal, balanced microbiome architecture, enhanced carbohydrate utilization, coordinated mineral acquisition, and strong antioxidant and immune buffering. In contrast, the mixed consortium promoted a digestion-dominated, high-metabolic-output state defined by proteolysis-driven energy production, Bacillus-dominated microbiota, moderated ion uptake, and peroxide-regulated detoxification. Despite producing similar growth gains, these strategies differ in energy efficiency, microbiome stability, and stress resilience, indicating that probiotic efficacy depends on how hepatopancreatic homeostasis is reprogrammed rather than on simple increases in microbial diversity or enzyme activity. The results highlight the hepatopancreas as the central integrative organ linking microbiome composition, mineral balance, and host proteome to growth performance, and demonstrate that precision selection of probiotic strains can direct shrimp metabolism toward either rapid nutrient turnover or efficient, stress-resistant production. These findings highlight that probiotic efficacy depends on targeted functional remodeling rather than mere microbial addition, providing a framework for precision interventions in sustainable shrimp aquaculture.

## Data Availability

The datasets presented in this study can be found in online repositories. The names of the repository/repositories and accession number(s) can be found at: https://www.ncbi.nlm.nih.gov/, PRJNA1158989; https://ngdc.cncb.ac.cn/omix, PRJCA042022.

## Footnotes

1
https://www.msn.cn/zh-cn/weather/forecast/

2
https://cloud.majorbio.com

## Glossary

ACE: Abundance-based coverage estimator
ACTN1_4: Alpha-actinin, sarcomeric
AMPK: AMP-activated protein kinase
AMPs: Antimicrobial peptides
ANT: ADP/ATP translocase
ATPeF1A: ATP synthase subunit alpha
ATPeF1B: ATP synthase subunit beta
Ca^2+^ ATPase: Calcium-transporting ATPase
CAT: Catalase
CTLDs: C-type lectin domain-containing protein
CTLDs-P: C-type lectin-like domain-containing protein PtLP
CTSD: Cathepsin D-like protein/cathepsin D
DIA: 4D data-independent acquisition
DEPs: Differentially expressed proteins
FBPase: Fructose-bisphosphatase
FUCA: Alpha-L-fucosidase
GBE1: Beta-1,3-glucan-binding protein
GO: Gene Ontology
GST: Glutathione transferase
HCP: Hemolymph clottable protein
HEXA_B: Beta-N-acetylhexosaminidase
HEXA_B: Beta-hexosaminidase
HSP70/60: Heat shock protein 70/60
IAM: Iodoacetamide
ITGB1: Integrin beta
KEGG: Kyoto Encyclopedia of Genes and Genomes
LAMA3_5: Laminin subunit alpha
LAMB1: Laminin subunit beta-1
LDA: Linear discriminant analysis
LEfSe: Linear discriminant analysis effect size
LMNB: Lamin Dm
MS: Mass spectrometry
MT: Metallothionein
NCBI: National Center for Biotechnology Information
NET: Neutrophil extracellular trap formation
PCoA: Principal coordinate analysis
PO: Phenoloxidase
PRRs: Pattern recognition receptors
PT44: Peritrophin-44-like protein
RAB1A: Organellar Rab protein
SCP-A: Sarcoplasmic calcium-binding protein variant A
SELENBP1: Putative selenium-binding protein 1
TEAB: Triethylammonium bicarbonate buffer
TCEP: Tris (2-carboxyethyl) phosphine
TEM: Leukocyte transendothelial migration
TRXA: Thioredoxin
TUBA: Tubulin alpha chain
TXNDC-X7: Thioredoxin domain-containing protein
TXNDC-Z5: Thioredoxin domain-containing protein
VDAC: Voltage-dependent anion-selective channel
XTD: Xanthine dehydrogenase
α2M: Alpha-2-macroglobulin
