# Supplementary material for: Lactobacillus salivarius GZPH2 reshapes hepatopancreatic microbiome structure and enhances immunometabolism in Litopenaeus vannamei under farm conditions
Source: Front Microbiol. 2026 Apr 16;17:1762396. doi: 10.3389/fmicb.2026.1762396 (PMC13128629; doi:10.3389/fmicb.2026.1762396)
Supplement: Supplementary file 1 [file Data_Sheet_1.pdf]

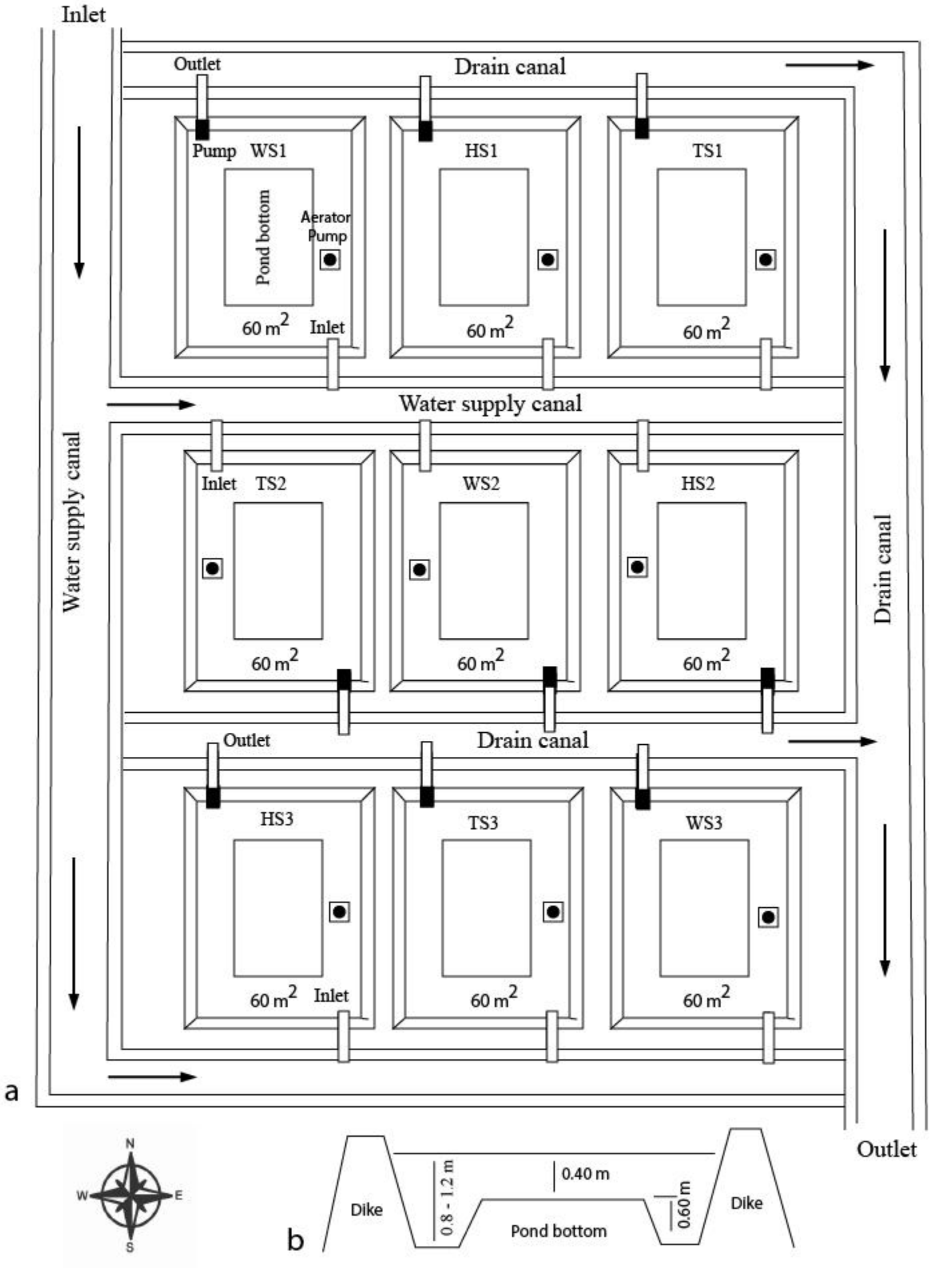

Supplementary Figure 1. Schematic diagram of the experimental pond system used for shrimp cultivation. (A) Layout of nine uniform ponds (60 m² each) divided into three treatment groups with three replicates: control W (WS1, WS2 and WS3) without probiotics, group H (HS1, HS2 and HS3) supplemented with *Lactobacillus salivarius*, and group T (TS1, TS2 and TS3) supplemented with a commercial EM probiotic. Water was supplied via a central canal and drained through side canals. Each pond was equipped with a pump and aerator. (B) Cross-sectional profile of a representative pond showing the dike (0.8–1.2 m height), bottom slope (0.4mm), and maintained water depth (0.6–0.9 m).

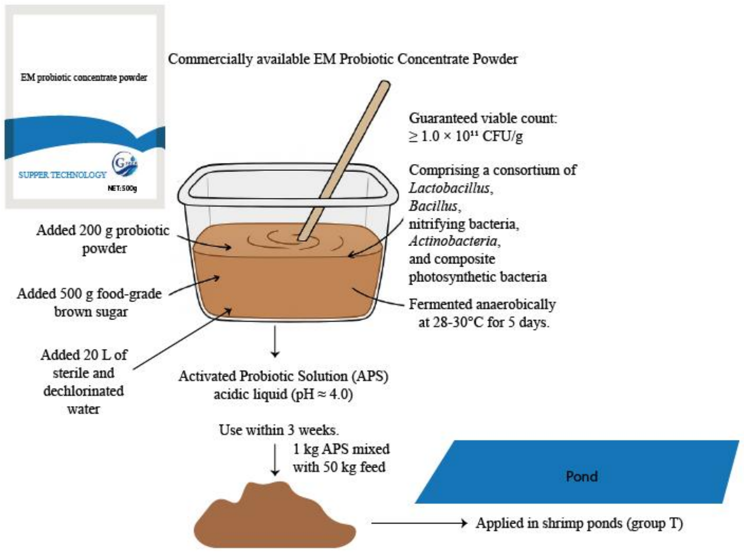

Supplementary Figure 2. Preparation and application of commercially available EM probiotic concentrate powder (comprising *Lactobacillus*, *Bacillus*, nitrifying bacteria, *Actinobacteria*, and photosynthetic bacteria) for shrimp cultivation.

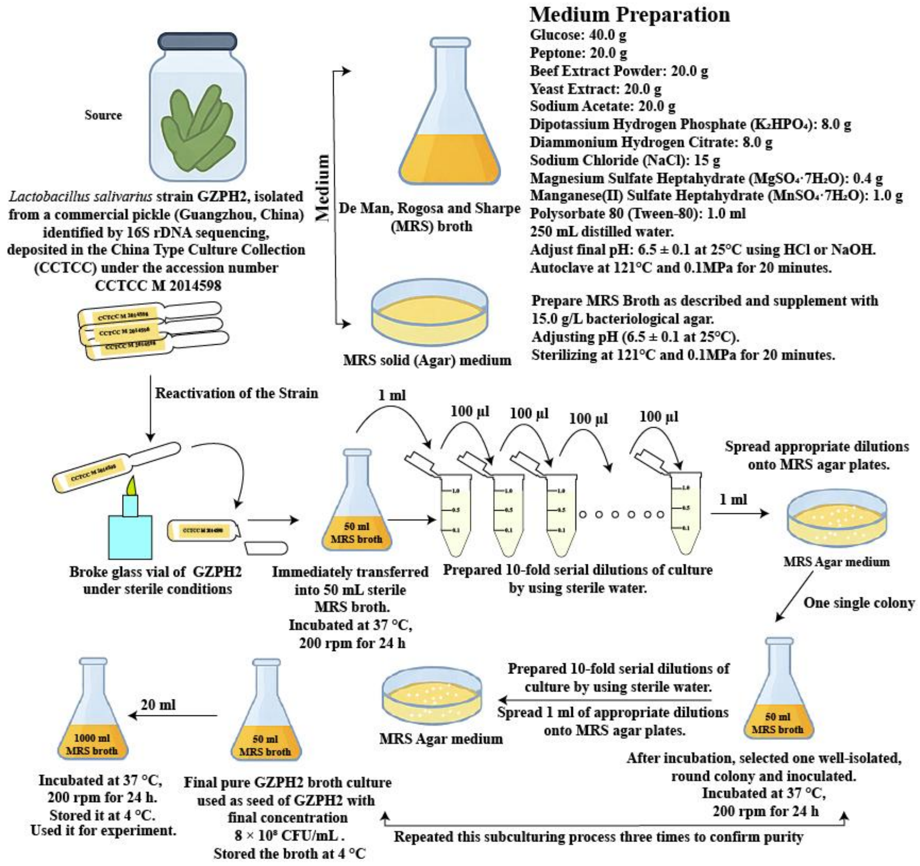

Supplementary Figure 3. Workflow for reactivation and culture purification of *Lactobacillus salivarius* strain GZPH2

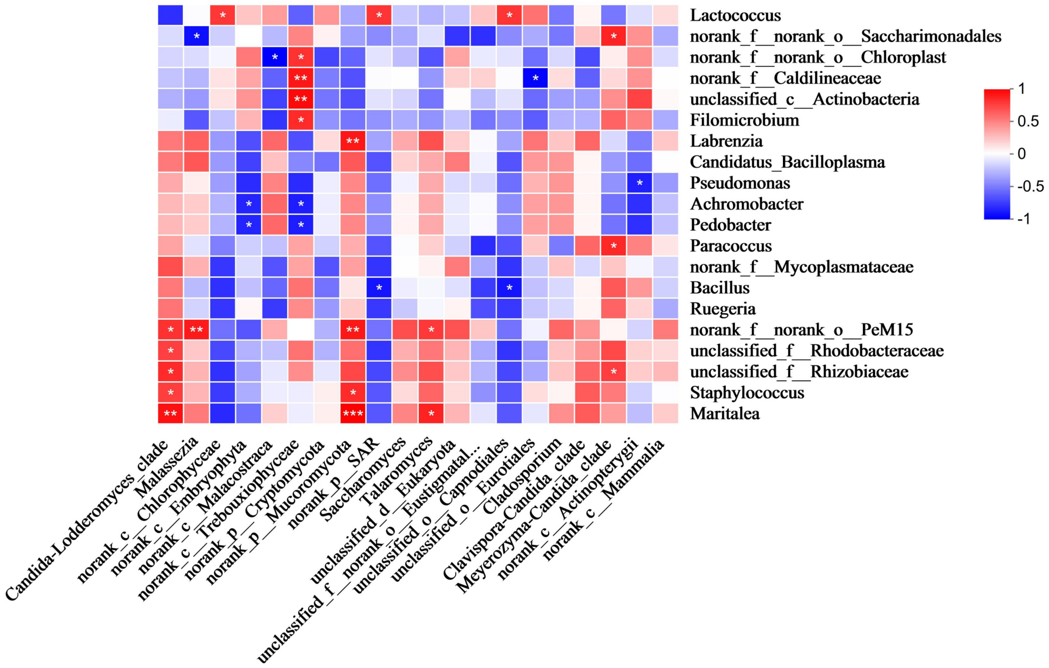

Supplementary Figure 4. Spearman correlation heatmap between the top 20 prokaryotic and eukaryotic genera. The x-axis shows eukaryotic genera; the y-axis shows prokaryotic genera. Correlation coefficient (r) is indicated by color, and significance is denoted by asterisks (*$P \leq 0.05$, **$P \leq 0.01$, ***$P \leq 0.001$). Correlation coefficient (r) is categorized as follows: $1.0 \geq r > 0.75$ indicates a very strongly positive correlation, $0.75 \geq r > 0.5$ indicates a strongly positive correlation, $0.50 \geq r > 0.25$ indicates a moderately positive correlation, $0.25 \geq r > 0$ indicates a weakly positive correlation, $r = 0$ indicates no correlation, $0.0 > r \geq -0.25$ indicates a weakly negative correlation, $-0.25 > r \geq -0.50$ indicates a moderately negative correlation, $-0.50 > r \geq -0.75$ indicates a strongly negative correlation, and $-0.75 > r \geq -1.0$ indicates a very strongly negative correlation.

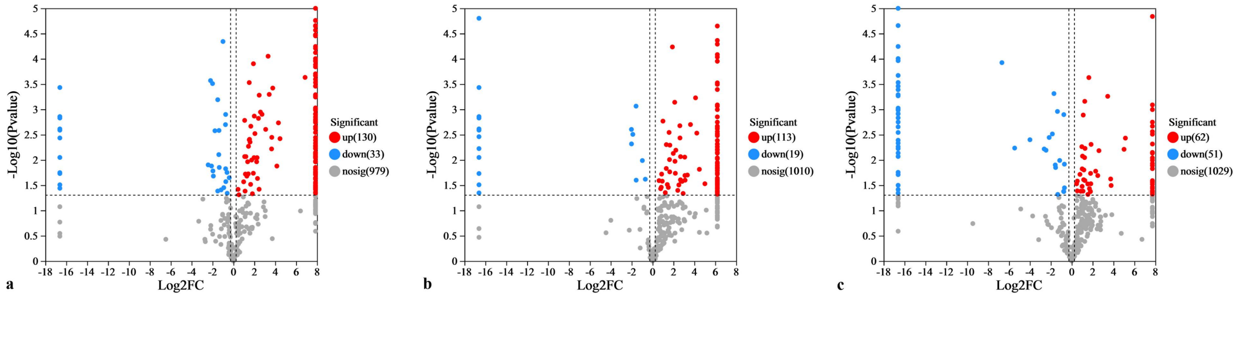

Supplementary Figure 5. Volcano plots of differentially expressed proteins (DEPs) in shrimp hepatopancreas. DEPs were identified in pairwise comparisons: (A) TH vs. WH, (B) HH vs. WH, and (C) HH vs. TH. Proteins are plotted based on their log2(fold-change) (x-axis) and statistical significance (-log10(p-value), y-axis). Red points denote significantly upregulated proteins, and blue points denote significantly downregulated proteins.

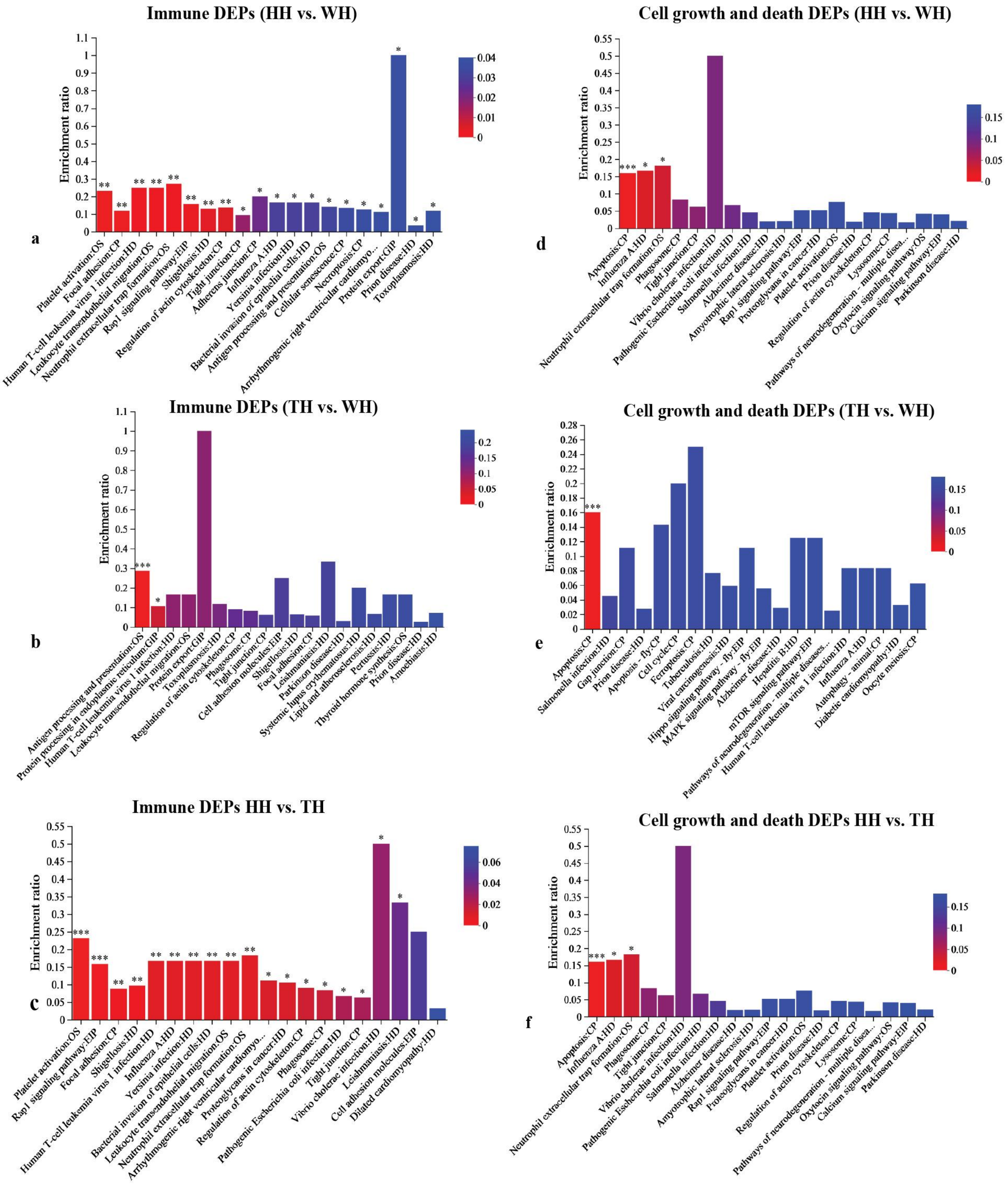

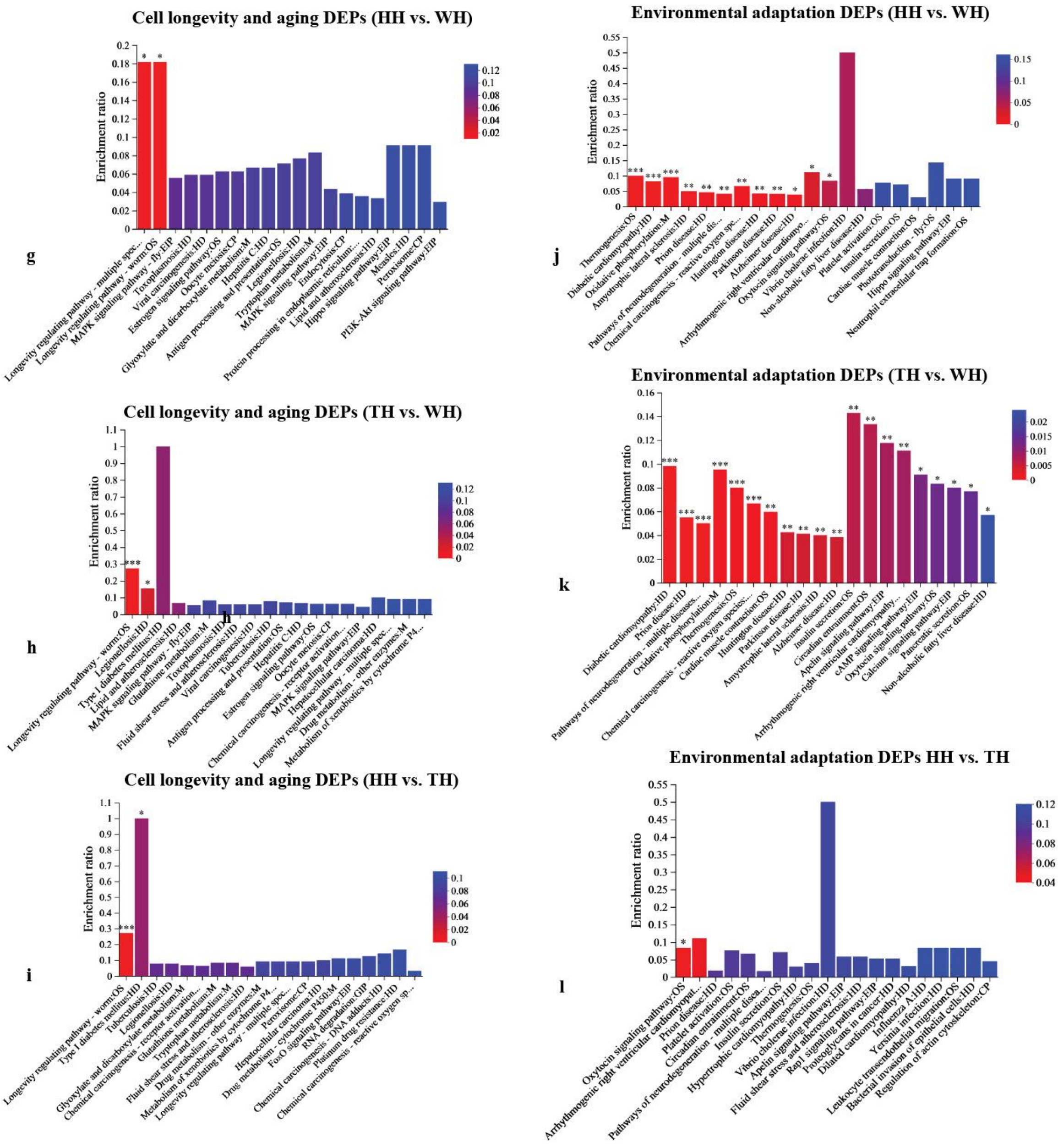

Supplementary Figure 6. KEGG pathway enrichment analysis of differentially expressed proteins (DEPs). Enriched pathways are grouped into four functional categories: (A-C) Immune system, (D-F) Cell growth and death, (G-I) Cellular aging and longevity, and (J-L) Environmental adaptation. The y-axis represents the enrichment factor, and the x-axis lists the pathway names. The color intensity indicates the statistical significance of enrichment (red, most significant), with significance levels denoted as **P* < 0.05, ***P* < 0.01, and ****P* < 0.001.

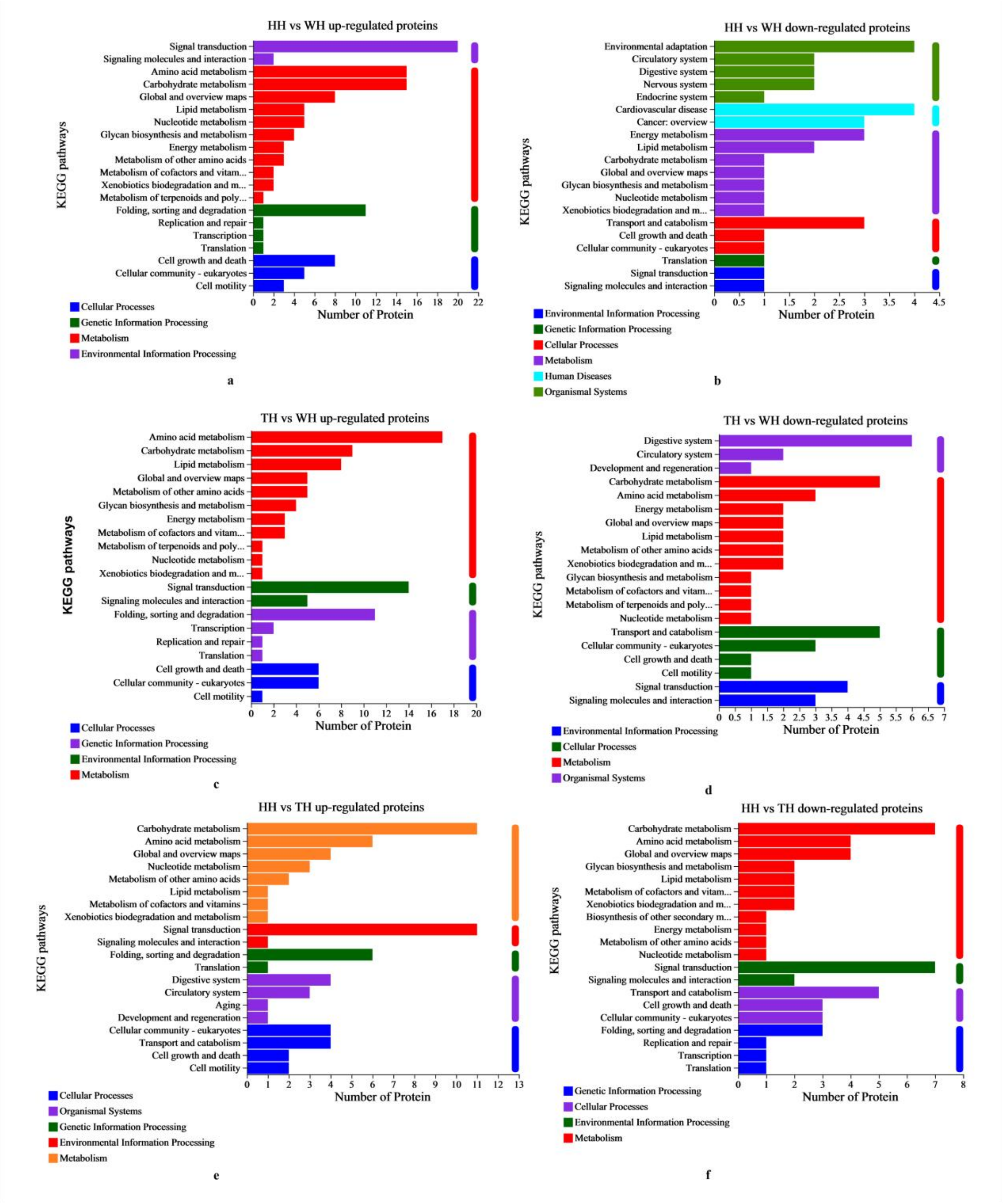

Supplementary Figure 7. KEGG pathway enrichment of all differentially expressed proteins (DEPs). Bar charts show the number of (A, C, E) upregulated and (B, D, F) downregulated DEPs annotated to specific metabolic pathways for the comparisons HH vs. WH, TH vs. WH, and HH vs. TH. Pathways are categorized into seven major KEGG groups and listed on the y-axis. The x-axis shows the number of DEPs per pathway.

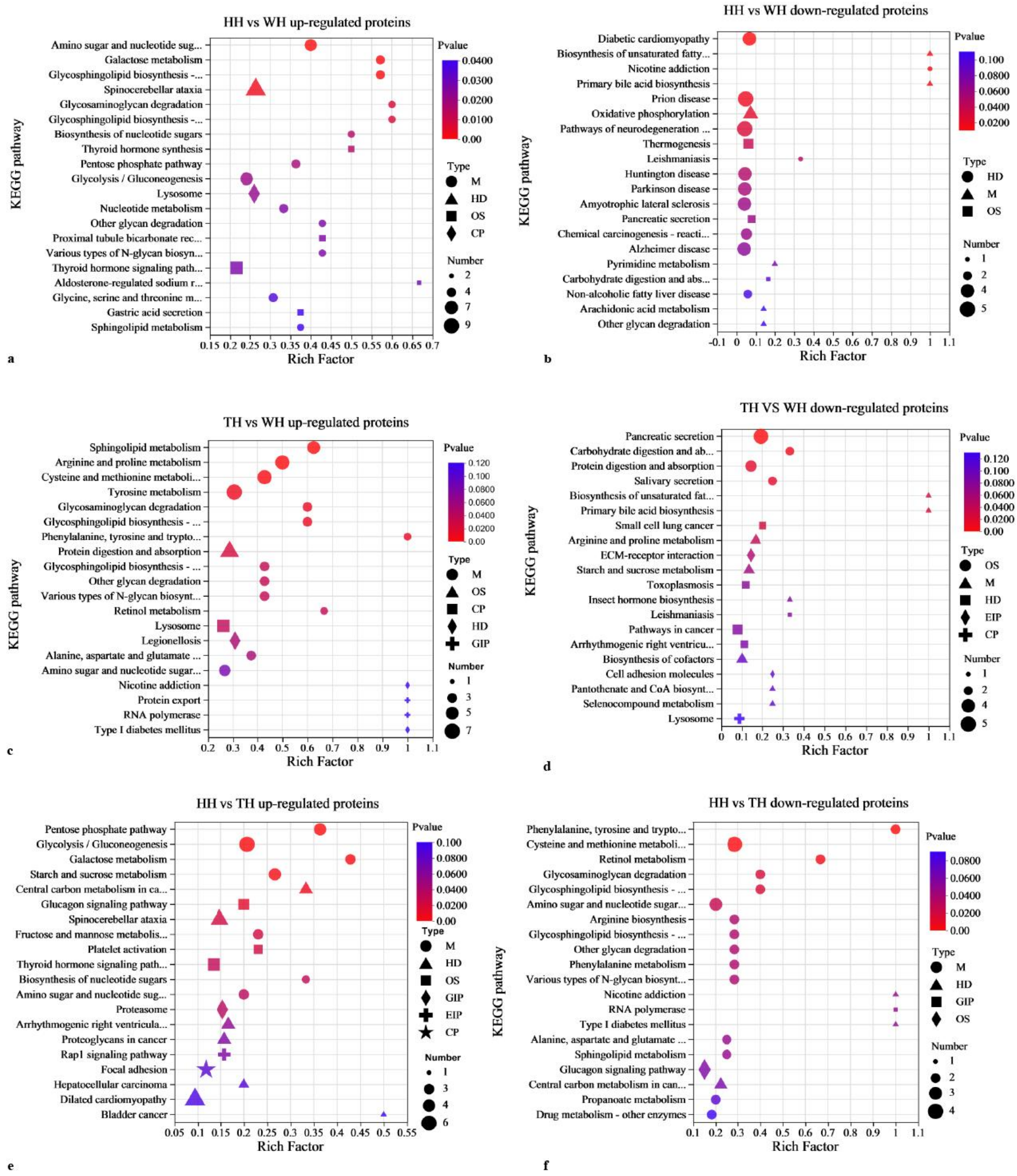

Supplementary Figure 8. KEGG pathway enrichment analysis of differentially expressed proteins (DEPs). Bubble charts display enriched pathways for (A, B) HH vs. WH, (C, D) TH vs. WH, and (E, F) HH vs. TH comparisons. The x-axis shows the enrichment factor; the y-axis lists pathway names. Point size corresponds to the number of DEPs per pathway; color represents the statistical significance (-log10(adjusted p-value)). Point shape denotes the KEGG category: Metabolism (M), Genetic Information Processing (GIP), Environmental Information Processing (EIP),

Cellular Processes (CP), Organismal Systems (OS), Human Diseases (HD), and Drug Development (DD).
