# Supplementary material for: Lactobacillus salivarius GZPH2 reshapes hepatopancreatic microbiome structure and enhances immunometabolism in Litopenaeus vannamei under farm conditions
Source: Front Microbiol. 2026 Apr 16;17:1762396. doi: 10.3389/fmicb.2026.1762396 (PMC13128629; doi:10.3389/fmicb.2026.1762396)
Supplement: Supplementary file 2 [file Data_Sheet_2.pdf]

Supplementary Table 1. Monthly average maximum and minimum temperature and precipitation of Haikou City, Hainan in 2023

**Months**	**Temperature (°C)**		**Precipitation (mm)**
	**Maximum**	**Minimum**	
January	24	11	10.69
February	28	17	7.37
March	30	20	9.03
April	31	21	2.58
May	33	24	47.44
June	32	27	49.08
July	32	27	78.85
August	32	27	30.55
September	30	26	27.6
October	28	24	56.2
November	27	19	28.55
December	26	14	5.62

Source: https://www.msn.cn/zh-cn/weather/forecast/

Supplementary Table 2. Pond water pH measurements during experimental period

	**Pre-change (immediately before 30% water exchange)**			**Post-change (immediately after exchange)**		
**Day**	**Group WH**	**Group HH**	**Group TH**	**Group WH**	**Group HH**	**Group TH**
0	7.50 ± 0.10[a]	7.50 ± 0.01[a]	7.50 ± 0.07[a]	-	-	-
7	8.25 ± 0.12[a]	8.10 ± 0.10[ab]	8.00 ± 0.08[b]	7.95 ± 0.10[a]	7.85 ± 0.08[a]	7.75 ± 0.07[a]
14	8.35 ± 0.14[a]	8.15 ± 0.11[ab]	8.05 ± 0.09[b]	8.05 ± 0.11[a]	7.90 ± 0.09[a]	7.80 ± 0.07[a]
21	8.45 ± 0.15[a]	8.25 ± 0.12[b]	8.05 ± 0.09[b]	8.10 ± 0.12[a]	7.95 ± 0.09[a]	7.80 ± 0.07[a]
28	8.55 ± 0.16[a]	8.30 ± 0.12[b]	8.10 ± 0.10[b]	8.20 ± 0.13[a]	8.00 ± 0.10[a]	7.85 ± 0.08[a]
35	8.60 ± 0.17[a]	8.35 ± 0.13[b]	8.15 ± 0.10[b]	8.25 ± 0.13[a]	8.05 ± 0.11[a]	7.90 ± 0.08[a]
42	8.55 ± 0.16[a]	8.20 ± 0.13[b]	8.10 ± 0.09[b]	8.20 ± 0.13[a]	8.00 ± 0.10[a]	7.85 ± 0.07[a]
49	8.60 ± 0.15[a]	8.30 ± 0.13[b]	8.05 ± 0.09[b]	8.15 ± 0.12[a]	8.00 ± 0.10[a]	7.85 ± 0.07[a]
56	8.45 ± 0.15[a]	8.25 ± 0.13[b]	8.00 ± 0.09[b]	8.10 ± 0.12[a]	7.95 ± 0.10[a]	7.80 ± 0.07[a]
63	8.50 ± 0.14[a]	8.20 ± 0.12[ab]	7.95 ± 0.09[b]	8.05 ± 0.11[a]	7.95 ± 0.10[a]	7.75 ± 0.07[a]
70	8.45 ± 0.13[a]	8.15 ± 0.12[ab]	7.90 ± 0.08[b]	8.00 ± 0.11[a]	7.90 ± 0.10[a]	7.70 ± 0.06[a]
77	8.45 ± 0.13[a]	8.10 ± 0.12[ab]	7.85 ± 0.08[b]	7.95 ± 0.11[a]	7.85 ± 0.09[a]	7.65 ± 0.06[a]
84	8.40 ± 0.14[a]	8.10 ± 0.12[ab]	7.85 ± 0.08[b]	8.00 ± 0.11[a]	7.90 ± 0.10[a]	7.65 ± 0.06[a]

Data were shown as mean ± standard deviation (n = 3). Different letters in the same column indicate significant difference ($P < 0.05$), and same letters indicate no difference ($P > 0.05$).

Supplementary Table 3. Nitrogen dynamics measurements in shrimp pond water during experimental period

Day	Group	Pre-change (immediately before 30% water exchange)			Post-change (immediately after exchange)		
		**TAN ($mg \cdot L^{-1}$)**	**NO_2^-–N ($mg \cdot L^{-1}$)**	**NO_3^-–N ($mg \cdot L^{-1}$)**	**TAN ($mg \cdot L^{-1}$)**	**NO_2^-–N ($mg \cdot L^{-1}$)**	**NO_3^-–N ($mg \cdot L^{-1}$)**
0	WH	0.82 ± 0.08^{a}	0.08 ± 0.01^{a}	4.0 ± 0.8^{a}	-	-	-
	HH	0.82 ± 0.07^{a}	0.08 ± 0.02^{a}	4.0 ± 0.6^{a}	-	-	-
	TH	0.82 ± 0.06^{a}	0.08 ± 0.01^{a}	4.0 ± 0.9^{a}	-	-	-
7	WH	1.20 ± 0.10^{a}	0.22 ± 0.03^{a}	7.0 ± 0.8^{a}	0.84 ± 0.08^{a}	0.15 ± 0.02^{a}	4.9 ± 0.6^{a}
	HH	0.95 ± 0.08^{b}	0.13 ± 0.02^{b}	5.2 ± 0.7^{b}	0.67 ± 0.06^{a}	0.10 ± 0.02^{a}	3.9 ± 0.5^{a}
	TH	0.80 ± 0.06^{b}	0.10 ± 0.01^{c}	4.5 ± 0.5^{c}	0.56 ± 0.05^{a}	0.07 ± 0.01^{a}	3.2 ± 0.4^{a}
14	WH	1.50 ± 0.12^{a}	0.35 ± 0.04^{a}	9.0 ± 1.0^{a}	1.05 ± 0.09^{a}	0.22 ± 0.03^{a}	6.2 ± 0.7^{a}
	HH	1.05 ± 0.10^{b}	0.15 ± 0.03^{b}	5.5 ± 0.8^{b}	0.82 ± 0.08^{a}	0.13 ± 0.02^{a}	4.7 ± 0.6^{a}
	TH	0.95 ± 0.07^{c}	0.14 ± 0.02^{c}	5.2 ± 0.6^{b}	0.77 ± 0.06^{a}	0.12 ± 0.02^{a}	4.6 ± 0.5^{a}
21	WH	1.80 ± 0.15^{a}	0.50 ± 0.05^{a}	11.0 ± 1.2^{a}	1.25 ± 0.11^{a}	0.28 ± 0.03^{a}	7.5 ± 0.8^{a}
	HH	1.30 ± 0.12^{b}	0.22 ± 0.03^{b}	6.9 ± 0.9^{b}	0.95 ± 0.09^{a}	0.18 ± 0.02^{a}	5.2 ± 0.6^{a}
	TH	1.10 ± 0.08^{b}	0.20 ± 0.02^{b}	6.3 ± 1.0^{b}	0.75 ± 0.07^{a}	0.10 ± 0.01^{a}	4.9 ± 0.5^{a}
28	WH	2.10 ± 0.18^{a}	0.70 ± 0.06^{a}	13.0 ± 1.4^{a}	1.45 ± 0.13^{a}	0.35 ± 0.04^{a}	8.8 ± 0.9^{a}
	HH	1.35 ± 0.13^{b}	0.25 ± 0.04^{b}	7.8 ± .06^{b}	1.05 ± 0.10^{a}	0.22 ± 0.03^{a}	5.8 ± 0.1^{a}
	TH	1.05 ± 0.08^{b}	0.18 ± 0.02^{b}	6.5 ± 0.9^{b}	0.85 ± 0.08^{a}	0.13 ± 0.02^{a}	4.8 ± 0.5^{a}
35	WH	2.40 ± 0.20^{a}	0.90 ± 0.08^{a}	15.0 ± 1.6^{a}	1.60 ± 0.14^{a}	0.40 ± 0.05^{a}	9.8 ± 1.0^{a}
	HH	1.55 ± 0.14^{b}	0.35 ± 0.04^{b}	8.5 ± 1.1^{b}	1.10 ± 0.10^{a}	0.24 ± 0.03^{a}	6.2 ± 0.7^{a}
	TH	1.25 ± 0.08^{b}	0.30 ± 0.02^{b}	7.9 ± 0.7^{c}	0.90 ± 0.08^{a}	0.12 ± 0.02^{a}	5.8 ± 0.5^{a}
42	WH	2.60 ± 0.22^{a}	1.00 ± 0.09^{a}	16.0 ± 1.7^{a}	1.70 ± 0.15^{a}	0.45 ± 0.05^{a}	10.2 ± 1.1^{a}
	HH	1.60 ± 0.15^{b}	0.40 ± 0.05^{b}	10.0 ± 1.1^{b}	1.15 ± 0.10^{a}	0.25 ± 0.03^{a}	6.5 ± 0.7^{a}
	TH	1.40 ± 0.09^{b}	0.33 ± 0.02^{b}	9.5 ± 0.^{b}	0.95 ± 0.09^{a}	0.13 ± 0.02^{a}	5.6 ± 0.6^{a}
49	WH	2.70 ± 0.25^{a}	1.10 ± 0.10^{a}	17.0 ± 1.8^{a}	1.75 ± 0.16^{a}	0.48 ± 0.05^{a}	10.5 ± 1.1^{a}
	HH	1.70 ± 0.16^{b}	0.45 ± 0.05^{b}	10.5 ± 1.2^{b}	1.20 ± 0.11^{a}	0.26 ± 0.03^{a}	6.8 ± 0.7^{a}
	TH	1.37 ± 0.09^{b}	0.38 ± 0.02^{b}	9.5 ± 0.8^{c}	1.00 ± 0.09^{a}	0.13 ± 0.02^{a}	5.1 ± 0.6^{a}

Data were shown as mean ± standard deviation (n = 3). Different letters in the same column indicate significant difference ($P < 0.05$), and same letters indicate no difference ($P > 0.05$).

Supplementary Table 4. Nitrogen dynamics measurements in shrimp pond water during experimental period (continue)

		Pre-change (immediately before 30% water exchange)			Post-change (immediately after exchange)		
**Day**	**Group**	**TAN ($mg\cdot L^{-1}$)**	**NO_2^-–N ($mg\cdot L^{-1}$)**	**NO_3^-–N ($mg\cdot L^{-1}$)**	**TAN ($mg\cdot L^{-1}$)**	**NO_2^-–N ($mg\cdot L^{-1}$)**	**NO_3^-–N ($mg\cdot L^{-1}$)**
56	WH	2.80 ± 0.25^a	1.15 ± 0.11^a	17.5 ± 1.9^a	1.80 ± 0.17^a	0.50 ± 0.05^a	10.8 ± 1.1^a
	HH	1.75 ± 0.17^b	0.46 ± 0.05^b	10.8 ± 1.2^b	1.25 ± 0.11^a	0.27 ± 0.03^a	6.9 ± 0.7^a
	TH	1.45 ± 0.09^b	0.40 ± 0.02^b	9.9 ± 0.8^c	1.05 ± 0.09^a	0.13 ± 0.02^a	6.2 ± 0.6^a
63	WH	2.90 ± 0.27^a	1.20 ± 0.12^a	18.0 ± 2.0^a	1.85 ± 0.17^a	0.52 ± 0.05^a	11.0 ± 1.1^a
	HH	1.80 ± 0.17^b	0.48 ± 0.05^b	11.0 ± 0.8^b	1.30 ± 0.12^a	0.28 ± 0.03^a	7.0 ± 0.8^a
	TH	1.55 ± 0.10^b	0.45 ± 0.02^b	10.7 ± 0.8^b	1.10 ± 0.10^a	0.14 ± 0.02^a	6.3 ± 0.6^a
70	WH	3.00 ± 0.28^a	1.25 ± 0.12^a	18.5 ± 2.0^a	1.90 ± 0.18^a	0.54 ± 0.05^a	11.2 ± 1.1^a
	HH	1.85 ± 0.18^b	0.50 ± 0.05^b	11.2 ± 1.3^b	1.35 ± 0.12^a	0.28 ± 0.03^a	7.1 ± 0.8^a
	TH	1.55 ± 0.10^c	0.47 ± 0.02^c	10.5 ± 0.7^c	1.15 ± 0.10^a	0.14 ± 0.02^a	6.5± 0.6^a
77	WH	3.10 ± 0.30^a	1.28 ± 0.12^a	19.0 ± 2.1^a	1.95 ± 0.18^a	0.55 ± 0.05^a	11.4 ± 1.2^a
	HH	1.90 ± 0.18^b	0.52 ± 0.05^b	11.5 ± 1.3^b	1.40 ± 0.13^a	0.29 ± 0.03^a	7.2 ± 0.8^a
	TH	1.65 ± 0.10^b	0.52 ± 0.02^b	11.0 ± 0.9^b	1.15 ± 0.10^a	0.14 ± 0.02^a	6.9 ± 0.6^a
84	WH	3.20 ± 0.30^a	1.30 ± 0.13^a	19.5 ± 2.2^a	2.00 ± 0.20^a	0.56 ± 0.06^a	11.6 ± 1.2^a
	HH	1.95 ± 0.19^b	0.55 ± 0.05^b	12.0 ± 1.0^b	1.02 ±0.09^a	0.20 ±0.03^a	7.7 ±0.9^a
	TH	1.70 ± 0.11^b	0.55 ± 0.02^b	11.8± 0.9^b	0.49 ±0.04^a	0.04 ±0.01^a	7.5 ±0.5^a

Data were shown as mean ± standard deviation (n = 3). Different letters in the same row indicate significant difference ($P < 0.05$), and same letters indicate no difference ($P > 0.05$).

Supplementary Table 5. Average body weight (g) of shrimp in experimental groups

Day	WH	HH	TH
0	0.10 ± 0.01^a	0.10 ± 0.01^a	0.10 ± 0.01^a
7	1.28 ± 0.02^a	1.31 ± 0.02^a	1.30 ± 0.02^a
14	2.40 ± 0.02^a	2.46 ± 0.02^a	2.45 ± 0.02^a
21	3.60 ± 0.02^a	3.68 ± 0.02^a	3.66 ± 0.02^a
28	4.60 ± 0.02^a	4.71 ± 0.02^a	4.70 ± 0.02^a
35	6.40 ± 0.03^a	6.55 ± 0.02^a	6.53 ± 0.02^a
42	7.90 ± 0.04^a	8.05 ± 0.03^a	8.03 ± 0.03^a
49	9.70 ± 0.30^a	10.20 ± 0.28^a	10.15 ± 0.29^a
56	11.50 ± 0.50^{ab}	12.30 ± 0.45^a	12.25 ± 0.46^a
63	13.30 ± 0.60^b	14.40 ± 0.50^a	14.35 ± 0.52^a
70	15.20 ± 0.58^b	16.20 ± 0.35^a	16.15 ± 0.36^a
77	17.40 ± 0.70^b	18.60 ± 0.40^a	18.70 ± 0.38^a
84	19.50 ± 0.95^b	21.80 ± 0.55^a	22.10 ± 0.30^a
90	21.33 ± 1.15^b	24.41 ± 0.62^a	24.85 ± 0.22^a

Data were shown as mean ± standard deviation (n = 50). Different letters in the same row indicate significant difference ($P < 0.05$), and same letters indicate no difference ($P > 0.05$).

Supplementary Table 6. Top 50 most-abundant KEGG Orthologs (KO) involved in metal ion transport and homeostasis as predicted by PICRUSt2 from 16S rRNA data

KO ID	KO Description	WH (mean)	HH (mean)	TH (mean)	Highest Group
K02015	Iron complex transport system permease protein	200037.34	276342.53	167756.28	HH
K02014	Iron complex outer membrane receptor protein	172627.5	243604.89	41206.06	HH
K02013	Iron complex transport ATP-binding protein	185515.7	219763.53	111768.8	HH
K02016	Iron complex substrate-binding protein	143890.1	227420.81	155956.99	HH
K02010	Iron(III) transport ATP-binding protein	95955.24	149860.52	53277.42	HH
K02012	Iron(III) transport substrate-binding protein	96439.52	157532.02	67689.93	HH
K03711	Ferric uptake regulator (Fur)	39317.14	72282.67	49985.12	HH
K16091	Fe(III) dicitrate transport protein	13821	19436.68	2553.19	HH
K13255	Ferric iron reductase FhuF	0.03	83.26	10.6	HH
K07243	High-affinity iron transporter	13824	22190.04	9743.83	HH
K16264	Cobalt-zinc-cadmium efflux protein	14103.74	35435.19	27147.92	HH
K15726	Heavy metal efflux system protein	20980.27	29746.64	4801.2	HH
K07239	Heavy metal exporter (HME family)	6.19	4.48	539.75	TH
K07787	Copper/silver efflux protein	3780.33	5950.02	1337.66	HH
K07245	Copper resistance protein D	13890.47	19537.65	5259.83	HH
K07233	Copper resistance protein B	13816.6	19042.93	2190.2	HH
K14166	Copper transport protein	58	8385.67	10982.5	TH
K01533	P-type Cu^{2+} transporter	33045.34	18788.18	13990.29	WH
K16267	Zinc and cadmium transporter	13523.5	18316.75	2217.25	HH
K09817	Zinc transport ATP-binding protein	29813.48	19241.1	19427.59	WH
K07238	Zinc transporter (ZIP family)	14247.34	19947.33	31033.78	TH
K02074	Zinc/manganese transport ATP-binding protein	13770.83	22318.44	8947.98	HH
K02006	Cobalt/Nickel transport ATP-binding protein	160	557.89	8762.44	TH
K02007	Cobalt/Nickel permease protein	156.67	558.89	8987.44	TH
K02008	Cobalt/Nickel permease protein	148.67	481.05	8737.44	TH

Supplementary Table 6. Top 50 most-abundant KEGG Orthologs (KO) involved in metal ion transport and homeostasis as predicted by PICRUSt2 from 16S rRNA data (continue)

KO ID	KO Description	WH (mean)	HH (mean)	TH (mean)	Highest Group
K07241	Nickel/Cobalt transporter (NiCoT)	55	2997.98	107.6	HH
K10824	Nickel transport ATP-binding protein	0.03	6701.2	34.32	HH
K15584	Nickel transport substrate-binding protein	0.03	3829.2	23.32	HH
K11710	Mn/Zn/Fe transport ATP-binding protein	188.67	5813.17	3180.17	HH
K11607	Mn/Fe transport ATP-binding protein	57.78	933.38	1125.6	TH
K03322	Manganese transport protein	46309.4	30767.98	14224.22	WH
K06189	Magnesium/Cobalt transporter	13956.08	21906.39	7483.91	HH
K03404	Magnesium chelatase subunit D	228.67	3715.29	6934	TH
K03403	Magnesium chelatase subunit H	161.33	4591.4	6485.75	TH
K03405	Magnesium chelatase subunit I	3964.67	9969.39	3109.85	HH
K00383	Glutathione reductase	42793.44	30423.16	14401.16	WH
K13892	Glutathione transport ATP-binding protein	40594.44	57757.57	10091.1	HH
K13889	Glutathione substrate-binding protein	27044.67	36637.12	4214.6	HH
K11747	Glutathione-regulated K^+ efflux protein	27315.13	41066.37	4942.66	HH
K03325	Arsenite transporter	17463.57	28164.8	17858.31	HH
K04565	Cu-Zn superoxide dismutase	13662.47	21007.1	19612.93	HH
K04564	Fe-Mn superoxide dismutase	64235.31	69826.73	59657.82	HH
K09823	Zinc uptake regulator	14078.58	26301.05	8731.91	HH
K09826	Iron response regulator	399.11	6952.53	5667.5	HH
K07226	Heme iron utilization protein	531.44	5298.79	4098.56	HH
K07227	Heme iron utilization protein	13.01	412.33	491.96	TH
K01990	ABC-2 type transport ATP-binding	1824.6	1711.3	1689.5	WH
K01992	ABC-2 type transport permease	1791.4	1654.8	1632.7	WH
K02003	Putative ABC transport ATP-binding	821.5	799.4	784.7	WH
K02004	Putative ABC permease	798.6	805.8	812.4	TH

Supplementary Table 7. The basic information of identified proteins

Annotation database	Number of comments	Total number of proteins	Annotation rate	e_value
NR	352	353	99.90 %	-
SubCell-Location	353	353	100 %	-
GO	293	353	83.05 %	1e-5
EggNOG	330	353	93.60 %	1e-5
KEGG	281	353	79.50 %	1e-5
Pfam	326	353	92.23 %	1e-5

Supplementary Table 8. DEPs involved in mineral transport and absorption pathways in the hepatopancreas of shrimp from the experimental three groups

Accession	Protein Name	Symbol	KO ID	Pathway (KEGG)	HH/ WH	TH/ WH	HH/ TH	Functional Category
A0A139Z424	Na+/K+-transporting ATPase subunit alpha	ATP1A	K01539	Mineral absorptio; Thyroid hormone synthesis	UP	UP	UP	Na+/K+ Exchange; Osmoregulation
A0A3R7M5U5	Na+/K+-transporting ATPase subunit beta	ATP1B	K01540	Mineral absorptio; Gastric acid secretion	UP	UP	DOWN	Na+/K+ Pump Stability
A0A4Y5QSB0	Ca-transporting ATPase (SERCA)	ATP2A	K05853	Calcium signaling; Cardiac muscle contraction	UP	UP	UP	Ca2+ uptake into ER/SR
G0YVG1	Calmodulin	CaM	K02183	Calcium signaling; Neurotrophin	UP	UP	UP	Ca2+ sensor; Signal transduction
A0A423SW99	solute carrier family 39 (zinc transporter) ZIP1	ZIP1_2_3	K14709	Parkinson disease; Alzheimer disease	NF	UP	DOWN	Zn2+ uptake
A0A3R7NZC3	Sarcoplasmic calcium-binding protein (SCP-A)	HRC; SCP-A	K23450	Calcium signaling; Cardiac muscle contraction	UP	DOWN	UP	Ca2+ buffering
A0A423TL51	Voltage-dependent anion channel	VDAC2	K15040	Calcium signaling; Neurodegeneration	UP	UP	UP	Mitochondrial Ca2+ channel

Supplementary Table 9. DEPs detoxification and antioxidant defense related pathways in the hepatopancreas of shrimp from the experimental three groups

Accession	Protein Name	Symbol	KO ID	Pathway (KEGG)	HH/ WH	TH/ WH	HH/ TH	Functional Category
A0A3R7QHC4	Catalase	CAT	K03781	Peroxisome; Longevity	UP	NF	UP	H2O2 decomposition
A0A3R7PJ04	Glutathione peroxidase 6	GPx6	K00432	Glutathione metabolism	UP	UP	DOWN	Lipid peroxide reduction
A0A3R7LRU8	Thioredoxin	trxA	K03671	NOD-like receptor	UP	UP	DOWN	Disulfide reduction
D0FH89	Peroxiredoxin (PRDX1)	PRDX1	K13279	Peroxisome; Cobalamin transport and metabolism	UP	UP	DOWN	ROS scavenging
A0A3R7PDX7	Peroxiredoxin (PRDX3)	PRDX3	K20011	-	UP	UP	DOWN	Mitochondrial ROS (mROS)
A0A3R7M2H6	Glutathione S-transferase (Isoform 1)	GST	K00799	Glutathione metabolism	UP	NF	UP	Xenobiotic conjugation
A0A423TR82	Glutathione S-transferase (Isoform 2)	GST	K00799	Glutathione metabolism	NF	UP	DOWN	Xenobiotic conjugation
A0A059P5D6	Metallothionein	MT4	K14741	-	UP	UP	UP	Heavy metal detox/storage
A0A3R7PLZ1	Selenium-binding protein 1	SELENBP1	K17285	Sulfur metabolism	UP	UP	UP	Selenium transport; Detox
A0A423TEZ4	Cystathionine gamma-lyase (CTH)	CTH	K01758	Cysteine metabolism	UP	UP	UP	Glutathione synthesis; H2S production
A0A3R7PF73	Cystathionine beta-synthase (CBS)	CBS	K01697	Cysteine metabolism	DOWN	DOWN	UP	Homocysteine metabolism
A0A3R7NV26	Dimethylglycine dehydrogenase	DMGDH	K00315	Glycine metabolism	UP	UP	UP	Amino acid detox
A0A3R7Q7A4	Aldehyde dehydrogenase	ALDH	K00128	Glycolysis; Fatty acid	UP	DOWN	UP	Aldehyde detox
A0A3R7M6S0	Epoxide hydrolase	EPHX1	K01253	Xenobiotics metabolism	NF	UP	DOWN	Toxin hydrolysis
A0A075DZ12	Glutamate dehydrogenase (GDH)	GDH	K00261	Nitrogen metabolism	UP	UP	UP	Ammonia detox

Supplementary Table 10. DEPs linked to the immune system, cellular defense, and stress tolerance pathways in the hepatopancreas of shrimp from the experimental three groups (continue)

Accession	Protein name	HH/WH	TH/WH	HH/TH	Types	KEGG pathway
A0A3R7SND6	Crustin-like protein (CLP)	Up	Up	Up	Antimicrobial peptides (AMPs)	Unknown
A0A3R7PLZ1	Putative selenium-binding protein 1 (SELENBP1)	Up	Up	Up	Binding protein	NET, Necroptosis
A0A3R7Q1E9	Peritrophin-44-like protein	Up	Up	Up	Binding protein	Necroptosis, Cellular senescence, NLR, NET
A0A423T995	Peritrophin-44-like protein	Up	Up	Up	Binding protein	Focal adhesion
A0A423TBN1	Laminin subunit beta-1 (LAMB1)	Up	Up	Up	Binding protein	Unknown
A0A3R7SS49	Alpha-actinin, sarcomeric (ACTN1_4)	Up	Up	Up	Binding protein	Unknown
A0A3R7PK45	Tubulin alpha chain (TUBA)	Down	Down	Up	Binding protein	Unknown
A0A3R7NZC3	Sarcoplasmic calcium-binding protein variant a (SCP-A)	Up	Down	Up	Binding protein	PI3K-Akt, Focal adhesion, LTM, PA, Cell adhesion, ECM
G1FEX1	ADP/ATP translocase (ANT)	Up	Up	Down	Binding protein	Tight junction, Adherens junction, Focal adhesion, LTM, Regulation of actin cytoskeleton

Supplementary Table 10. DEPs linked to the immune system, cellular defense, and stress tolerance pathways in the hepatopancreas of shrimp from the experimental three groups (continue)

Accession	Protein name	HH/WH	TH/WH	HH/TH	Types	KEGG pathway
A0A423TL51	Voltage-dependent anion-selective channel (VDAC)	Up	Up	Up	Binding protein	Tight junction, Adherens junction, Focal adhesion, Cell adhesion, PI3K-Akt, LTM, PA, Rap1, *Yersinia* infection, Pathogenic *E. coli* infection, Bacterial invasion of epithelial cells
A0A423U413	Integrin beta (ITGB1)	Up	Down	Up	Binding protein	Apoptosis, Phagosome, Pathogenic *Escherichia coli* (*E. coli*) infection, Salmonella infection
Q9GSP9	β-actin	Up	Up	Up	Cytoskeletal protein	Thermogenesis, LTM, PA, NET, Hippo, Rap1, Apoptosis, Focal adhesion, Phagosome, *Vibrio cholerae (V. cholerae)* infection, *Yersinia* infection, Pathogenic *E. coli* infection, *Salmonella* infection, Bacterial invasion of epithelial cells
A0A3R7LT31	Alpha-L-fucosidase (FUCA)	Down	Down	Down	Enzymes	Lysosome
A0A3R7SVI1	Beta-hexosaminidase (HEXA_B)	Up	Up	Down	Enzymes	Glycosphingolipid biosynthesis - ganglio series, Lysosome
A0A3R7QHC4	Catalase (CAT)	Up	NF	Up	Enzymes	Longevity regulating, FoxO

Supplementary Table 10. DEPs linked to the immune system, cellular defense, and stress tolerance pathways in the hepatopancreas of shrimp from the experimental three groups (continue)

Accession	Protein name	HH/WH	TH/WH	HH/TH	Types	KEGG pathway
A0A423U8Z5	Thioredoxin domain-containing protein (TXNDC-Z5)	Down	Down	Up	Enzymes	Unknown
A0A075DZ12	Glutamate dehydrogenase (NAD(P) (GLUD1_2)	Up	NF	Up	Enzymes	Necroptosis
A0A423TR82	Glutathione transferase (GST)	NF	Up	Down	Enzymes	Longevity regulating pathway
A0A3R7LRU8	Thioredoxin (TRXA)	NF	Up	Down	Enzymes	Salmonella infection, NLR
A0A423SX77	Heat shock protein 70 (HSP70)	Up	NF	Up	Heat shock protein	MAPK, Estrogen signaling, Longevity regulating pathway
A0A3R7MHJ9	Heat shock protein 70 (HSP70)	Up	Up	Up	Heat shock protein	MAPK, Longevity regulating, Antigen processing, Estrogen signaling
A0A3R7MS79	Heat shock protein 60 (HSP60)	NF	Up	Down	Heat shock protein	Longevity regulating, RNA degradation
A0A3R7PUZ2	Hemocyanin (HMC)	Up	Up	Up	Hemocyanin	Tyrosine metabolism, Glycerophospholipid metabolism, Melanogenesis
A0A3R7Q123	Hemocyanin (HMC)	Up	Up	Up	Hemocyanin	Tyrosine metabolism, Melanogenesis
A0A3R7P7W3	Hemocyanin (HMC)	Up	Up	Up	Hemocyanin	Tyrosine metabolism, Melanogenesis
A0A3R7LSN5	Hemocyanin subunit L1 (HMCSL1)	Down	Up	Down	Hemocyanin	Tyrosine metabolism, Melanogenesis
A0A3R7PVP7	Hemocyanin (HMC)	Down	Up	Down	Hemocyanin	Tyrosine metabolism, Melanogenesis
A0A3R7PEN2	Hemocyanin (HMC)	Down	Up	Down	Hemocyanin	Tyrosine metabolism, Melanogenesis

Supplementary Table 10. DEPs linked to the immune system, cellular defense, and stress tolerance pathways in the hepatopancreas of shrimp from the experimental three groups (continue)

Accession	Protein name	HH/WH	TH/WH	HH/TH	Types	KEGG pathway
A0A423TSJ7	Angiopoietin-related protein 1 (ANGPT1)	Up	NF	Up	Immuno-regulation and inflammation	Unknown
A0A2L0HIL1	Macrophage migration inhibitory factor (MIF)	Up	Up	Up	Immuno-regulation and inflammation	Unknown
J7K1E9	Bip	Up	Up	Up	Immuno-regulation and inflammation	Antigen processing
A0A423SAG9	Cathepsin D-like protein (CTSD)	Up	Up	Down	Lysosomal peptidase	Apoptosis, Lysosome, Estrogen signaling, Autophagy
AOA423SFG7	Dipeptidyl peptidase 1 (cathepsin C)	Up	Up	Up	Lysosomal peptidase	Apoptosis, Lysosome
A0A3R7SR07	Cathepsin D (CTSD)	NF	Up	Down	Lysosomal peptidase	Apoptosis, Lysosome, Sphingolipid signaling, Autophagy
A0A423SGI0	C-type lectin domain-containing protein (CTLDs)	Up	Up	Down	Pattern recognition receptors	Sphingolipid signaling, Lysosome, Autophagy, Apoptosis
A0A423T231	Beta-1,3-glucan-binding protein (GBE1)	Up	Down	Up	Pattern recognition receptors	Unknown
A0A1P8DAA8	Fibrinogen-related protein 2 (FCN)	Up	Up	Up	Pattern recognition receptors	Unknown
A0A2D1UKD6	L-type lectin	Up	NF	Up	Pattern recognition receptors	Protein processing in endoplasmic reticulum
A0A3R7PKG4	Beta-1,3-glucan-binding protein	Up	Down	Up	Pattern recognition receptors	Unknown
A0A423T350	C-type lectin-like domain-containing protein PtLP (CTLDs-P)	Up	NF	Up	Pattern recognition receptors	Protein processing in endoplasmic reticulum

Supplementary Table 10. DEPs linked to the immune system, cellular defense, and stress tolerance pathways in the hepatopancreas of shrimp from the experimental three groups (continue)

Accession	Protein name	HH/WH	TH/WH	HH/TH	Types	KEGG pathway
A0A3R7LW48	Alpha-2-macroglobulin (α2M)	Up	Up	Up	Protease inhibitor	Unknown
A0A3R7PBU1	Alpha-2-macroglobulin (α2M)	Down	Up	Down	Protease inhibitor	Unknown
Q9GSP9	β-actin	Up	Up	Up	Cytoskeletal protein	Thermogenesis, LTM, PA, NET, Hippo, Rap1, Apoptosis, Focal adhesion, Phagosome, *V. cholerae* infection, *Yersinia* infection, Pathogenic *E. coli* infection, *Salmonella* infection, Bacterial invasion of epithelial cells

Supplementary Table 11. KEGG functional annotation and differential abundance of proteins involved in immune defense, virulence, toxin production, and antimicrobial peptide (AMP) synthesis across WH, HH, and TH groups

KO ID	KO_Description	WH (Mean ± SD)	HH (Mean ± SD)	TH (Mean ± SD)	Dominant Group	Role / Function
K01183	Chitinase [EC:3.2.1.14]	28825 ± 23900	3385 ± 2870	21422 ± 13050	WH	Breaks down chitin in fungal cell walls; involved in defense against fungal pathogens
K03781	Catalase [EC:1.11.1.6]	27687 ± 32795	49493 ± 5500	38538 ± 18773	HH	Converts hydrogen peroxide to water and oxygen; protects against oxidative stress from host immune response
K03810	Virulence factor	28669 ± 23800	1498 ± 1450	10592 ± 6600	WH	General virulence factor; contributes to pathogenicity and host immune evasion

Supplementary Table 11. KEGG functional annotation and differential abundance of proteins involved in immune defense, virulence, toxin production, and antimicrobial peptide (AMP) synthesis across WH, HH, and TH groups (continued)

KO ID	KO_Description	WH (Mean ± SD)	HH (Mean ± SD)	TH (Mean ± SD)	Dominant Group	Role / Function
K04565	Superoxide dismutase, Cu-Zn family [EC:1.15.1.1]	13662 ± 15084	21006 ± 7087	19613 ± 1667	HH	Detoxifies superoxide radicals; protects bacteria from oxidative burst during host immune response
K04784	Yersiniabactin nonribosomal peptide synthetase	0.033 ± 0.00	44.09 ± 75.19	0.033 ± 0.00	HH	Siderophore synthesis enzyme for iron scavenging and virulence
K04787	Mycobactin salicyl-AMP ligase	9.68 ± 8.55	22.67 ± 6.03	87.50 ± 66.50	TH	Activates salicylic acid for siderophore (mycobactin) biosynthesis for iron acquisition
K04788	Mycobactin phenyloxazoline synthetase	9.68 ± 8.55	26.00 ± 9.54	87.50 ± 66.50	TH	Catalyzes heterocycle formation in siderophore synthesis
K04789	Mycobactin peptide synthetase MbtE	9.68 ± 8.55	20.33 ± 5.86	87.50 ± 66.50	TH	NRPS enzyme for siderophore peptide chain assembly
K04790	Mycobactin polyketide synthetase MbtC	9.68 ± 8.55	20.33 ± 5.86	75.00 ± 54.00	TH	Polyketide synthase required for siderophore backbone formation
K04791	Mycobactin polyketide synthetase MbtD	9.68 ± 8.55	20.33 ± 5.86	75.00 ± 54.00	TH	Polyketide synthase involved in mycobactin biosynthesis
K04792	Mycobactin peptide synthetase MbtF	9.68 ± 8.55	20.33 ± 5.86	87.50 ± 66.50	TH	Non-ribosomal peptide synthetase involved in mycobactin siderophore synthesis

Supplementary Table 11. KEGG functional annotation and differential abundance of proteins involved in immune defense, virulence, toxin production, and antimicrobial peptide (AMP) synthesis across WH, HH, and TH groups (continued)

KO ID	KO_Description	WH (Mean ± SD)	HH (Mean ± SD)	TH (Mean ± SD)	Dominant Group	Role / Function
K04793	Mycobactin lysine-N-oxygenase	9.68 ± 8.55	25.00 ± 13.45	87.50 ± 66.50	TH	Oxidizes lysine during siderophore biosynthesis
K05375	MbtH protein	197 ± 250	1584 ± 1400	10726 ± 6550	TH	Involved in siderophore biosynthesis; helps bacteria acquire iron in iron-limited host environments
K05595	Multiple antibiotic resistance protein	31666 ± 37800	55625 ± 11250	14999 ± 7200	HH	Confers resistance to multiple antibiotics; helps bacteria survive during host treatment
K07213	Copper chaperone	14171 ± 16587	31069 ± 7654	23488 ± 1576	HH	Delivers copper to detoxifying enzymes; protects against copper toxicity used by host immune system
K07273	Lysozyme	32823 ± 19760	21654 ± 17870	13688 ± 4820	WH	Hydrolyzes peptidoglycan in bacterial cell walls; directly kills bacteria
K07449	DNA adenine methylase [EC:2.1.1.72]	410 ± 260	1535 ± 1600	14958 ± 11800	TH	Regulates gene expression through DNA methylation; involved in phase variation to evade host immunity
K09607	Immune inhibitor A [EC:3.4.24.-]	13 ± 22	4286 ± 4100	31822 ± 20000	TH	Metalloprotease that degrades host antimicrobial peptides and immune proteins
K11031	Thiol-activated cytolysin	4 ± 7	1309 ± 1390	10577 ± 6650	TH	Pore-forming toxin that binds cholesterol; lyses host cells and evades phagocytosis

Supplementary Table 11. KEGG functional annotation and differential abundance of proteins involved in immune defense, virulence, toxin production, and antimicrobial peptide (AMP) synthesis across WH, HH, and TH groups (continued)

KO ID	KO_Description	WH (Mean ± SD)	HH (Mean ± SD)	TH (Mean ± SD)	Dominant Group	Role / Function
K11041	Exfoliative toxin A/B	28659.27 ± 26118.40	132.81 ± 173.55	7.02 ± 7.01	WH	Serine protease toxin that cleaves host skin proteins causing tissue damage
K11068	Hemolysin III	56642 ± 10300	56910 ± 5300	31126 ± 10000	HH	Pore-forming toxin; lyses erythrocytes and other host cells; contributes to tissue damage
K13275	Major intracellular serine protease [EC:3.4.21.-]	4 ± 7	1319 ± 1380	10593 ± 6620	TH	Degrades host proteins; may interfere with host immune signaling pathways
K13280	Signal peptidase I [EC:3.4.21.89]	184 ± 250	1583 ± 1400	10860 ± 6400	TH	Cleaves signal peptides from secreted proteins; essential for processing virulence factors
K13300	Cytochrome c550	10 ± 4	1324 ± 1350	10662 ± 6530	TH	Part of electron transport chain; helps manage oxidative stress during host infection
K13611	Polyketide synthase PksJ	0.033 ± 0.00	0.033 ± 0.00	10.27 ± 10.23	TH	Polyketide synthase for secondary metabolite / antibiotic production
K13612	Polyketide synthase PksL	0.033 ± 0.00	0.033 ± 0.00	10.27 ± 10.23	TH	Polyketide synthase involved in antimicrobial compound biosynthesis

Supplementary Table 11. KEGG functional annotation and differential abundance of proteins involved in immune defense, virulence, toxin production, and antimicrobial peptide (AMP) synthesis across WH, HH, and TH groups (continued)

KO ID	KO_Description	WH (Mean ± SD)	HH (Mean ± SD)	TH (Mean ± SD)	Dominant Group	Role / Function
K13657	Alpha-1,3-mannosyltransferase [EC:2.4.1.252]	106 ± 50	3605 ± 5000	20 ± 9	HH	Modifies surface glycans; alters antigenicity to evade host immune recognition
K13993	HSP20 family protein	34428 ± 23652	41968 ± 8688	28246 ± 431	HH	Small heat shock protein; protects cells from stress, including oxidative and thermal stress during infection
K15655	Surfactin family lipopeptide synthetase B	0.033 ± 0.00	0.033 ± 0.00	20.52 ± 20.48	TH	Produces surfactin antimicrobial biosurfactant, and antifungal and antiviral
K15664	Fengycin family lipopeptide synthetase A	0.033 ± 0.00	0.033 ± 0.00	10.27 ± 10.23	TH	Initiates fengycin antimicrobial lipopeptide synthesis
K15666	Fengycin family lipopeptide synthetase C	0.033 ± 0.00	0.033 ± 0.00	20.52 ± 20.48	TH	NRPS enzyme producing fengycin antimicrobial lipopeptide, and antifungal
K15667	Fengycin family lipopeptide synthetase D	0.033 ± 0.00	0.033 ± 0.00	20.52 ± 20.48	TH	NRPS module for fengycin antibiotic biosynthesis
K15668	Fengycin family lipopeptide synthetase E	0.033 ± 0.00	0.033 ± 0.00	10.27 ± 10.23	TH	Final module of fengycin peptide assembly
K16079	Outer membrane immunogenic protein	289 ± 150	10610 ± 13000	3378 ± 2500	HH	Surface-exposed protein; may act as decoy antigen to divert host immune response

Supplementary Table 11. KEGG functional annotation and differential abundance of proteins involved in immune defense, virulence, toxin production, and antimicrobial peptide (AMP) synthesis across WH, HH, and TH groups (continued)

**KO ID**	**KO_Description**	**WH (Mean ± SD)**	**HH (Mean ± SD)**	**TH (Mean ± SD)**	**Dominant Group**	**Role / Function**
K16079	Outer membrane immunogenic protein	289 ± 150	10610 ± 13000	3378 ± 2500	HH	Surface-exposed protein; may act as decoy antigen to divert host immune response
K16127	Microcystin synthetase protein McyG	41.67 ± 43.21	17.01 ± 15.27	6054.00 ± 4750.70	TH	Non-ribosomal peptide synthetase producing microcystin toxin
K19449	Xenobiotic Response Element (XRE) family transcriptional regulator	6 ± 6	1324 ± 1360	10728 ± 6600	TH	Regulates biofilm formation and stress responses; helps bacteria persist in hostile environments

Supplementary Table 12. KEGG functional annotation and differential abundance of proteins involved in growth develpoment or tissue repair in hepatopancrease across WH, HH, and TH groups

KO ID	KO_Description	Role	WHS (Mean ± SD)	HHS (Mean ± SD)	THS (Mean ± SD)	Dominant Group
K00003	homoserine dehydrogenase [EC:1.1.1.3]	Provides precursors for protein synthesis (Methionine, Threonine).	46940.64 ± 3777.66	45254.27 ± 2885.32	44091.53 ± 59031.5	TH
K00134	glyceraldehyde 3-phosphate dehydrogenase [EC:1.2.1.12]	Central to energy (ATP) production via glycolysis.	54928.96 ± 7712.67	71471.21 ± 17601.07	61586.96 ±45563.76	HH
K00626	acetyl-CoA C-acetyltransferase [EC:2.3.1.9]	Involved in energy metabolism (fatty acid oxidation).	145182.23 ± 117152.82	207687.84 ± 40178.18	125831.74±84097.21	HH

Supplementary Table 12. KEGG functional annotation and differential abundance of proteins involved in growth develpoment or tissue repair in hepatopancrease across WH, HH, and TH groups (continued)

KO ID	KO_Description	Role	WHS (Mean ± SD)	HHS (Mean ± SD)	THS (Mean ± SD)	Dominant Group
K00647	3-oxoacyl-[acyl-carrier-protein] synthase I [EC:2.3.1.41]	Key for fatty acid synthesis (new cell membranes).	14226.08 ± 16738.79	27291.48 ± 4215.47	12610.97±3731.52	HH
K01251	adenosylhomocysteinase [EC:3.3.1.1]	Regulates methylation potential for cell proliferation.	43098.74 ± 4139.92	31802.44 ± 6521.81	32425.95±6739.88	WH
K01476	arginase [EC:3.5.3.1]	Produces ornithine for polyamines (cell proliferation) and proline for collagen.	41516.79 ± 4836.79	33500.54 ± 3634.00	16333.75±21486.18	WH
K01915	glutamine synthetase [EC:6.3.1.2]	Produces glutamine, a fuel for rapidly dividing cells.	66980.79 ± 21567.52	92147.64 ± 16766.85	99375.84±35155.31	HH
K01952	phosphoribosylformylglycinamidine synthase [EC:6.3.5.3]	Crucial for de novo purine nucleotide synthesis (DNA/RNA).	105485.90 ± 49614.68	66567.03 ± 28980.06	86643.69±64303.49	WH
K01953	asparagine synthase (glutamine-hydrolysing) [EC:6.3.5.4]	Produces asparagine, essential for protein synthesis.	49153.70 ± 40459.68	78485.81 ± 29441.91	40023.91±63707.45	HH
K01610	phosphoenolpyruvate carboxykinase (ATP) [EC:4.1.1.49]	Key enzyme in gluconeogenesis for energy and biosynthesis.	4231.86 ± 4311.06	19495.10 ± 14295.69	27577.11±20794.51	TH
K01689	enolase [EC:4.2.1.11]	Part of glycolysis, central to energy production.	53832.03 ± 3025.71	61965.75 ± 6010.92	38791.19± 27167.63	HH
K01834	2,3-bisphosphoglycerate-dependent phosphoglycerate mutase [EC:5.4.2.11]	Essential step in glycolysis for energy production.	42605.33 ± 7581.24	44356.43 ± 19512.08	29416.33± 89559.31	HH
K09458	3-oxoacyl-[acyl-carrier-protein] synthase II [EC:2.3.1.179]	Key for fatty acid synthesis (new cell membranes).	64538.34 ± 16658.61	72587.51 ± 6782.78	54098.25±29725.58	HH

Supplementary Table 12. KEGG functional annotation and differential abundance of proteins involved in growth develpoment or tissue repair in hepatopancrease across WH, HH, and TH groups (continued)

KO ID	KO_Description	Role	WHS (Mean ± SD)	HHS (Mean ± SD)	THS (Mean ± SD)	Dominant Group
K09969	general L-amino acid transport system substrate-binding protein	Imports amino acids, providing building blocks for protein synthesis.	95470.44 ± 95806.67	143604.01 ± 53349.81	33989.61±19603.5	HH
K10780	enoyl-[acyl-carrier protein] reductase III [EC:1.3.1.104]	Involved in fatty acid synthesis for new membranes.	4.68 ± 4.05	14.01 ± 20.25	191.5± 81	TH
K00873	pyruvate kinase [EC:2.7.1.40]	Final glycolytic enzyme - converts phosphoenolpyruvate to pyruvate, generating ATP via Glycolysis	47316 ± 4185	45315 ± 3028	48647 ± 5023	TH
K00666	1-acyl-sn-glycerol-3-P acyltransferase	Lipid synthesis	4618±3506	6663±2651	3055±922	HH

Supplementary Table 13. KEGG functional annotation and differential abundance of proteins involved in metabolism/digestion across WH, HH, and TH groups

KO ID	KO_Description	Pathway/Function	WHS (Mean ± SD)	HHS (Mean ± SD)	THS (Mean ± SD)	Dominant Group
K00260	glutamate dehydrogenase [EC:1.4.1.2]	Amino acid metabolism (glutamate)	24.34 ± 17.71	6856.52 ± 8987.29	10843.97 ± 9229.06	TH
K01046	triacylglycerol lipase [EC:3.1.1.3]	Lipid digestion	489.87 ± 518.97	14905.42 ± 19748.37	21758.50 ± 18135.39	TH
K05349	beta-glucosidase [EC:3.2.1.21]	Carbohydrate digestion (cellulose)	11129.78 ± 12282.19	15922.92 ± 8194.72a	11637.74 ± 11037.21	HH
K00042	2-hydroxy-3-oxopropionate reductase [EC:1.1.1.60]	Glyoxylate/dicarboxylate metabolism	664.83 ± 541.05	9102.25 ± 7268.15	23369.18 ± 16614.97	TH

Supplementary Table 13. KEGG functional annotation and differential abundance of proteins involved in metabolism/digestion across WH, HH, and TH groups (continued)

KO ID	KO_Description	Pathway/Function	WHS (Mean ± SD)	HHS (Mean ± SD)	THS (Mean ± SD)	Dominant Group
K00231	protoporphyrinogen/coproporphyrinogen III oxidase [EC:1.3.3.4 1.3.3.15]	Porphyrin metabolism	159.80 ± 111.43	8392.17 ± 8635.68	23723.56 ± 16089.26	TH
K00899	5-methylthioribose kinase [EC:2.7.1.100]	Methionine salvage / Amino acid metabolism	30.68 ± 33.16	2824.09 ± 3281.04	21314.19 ± 18681.64	TH
K01582	lysine decarboxylase [EC:4.1.1.18]	Amino acid metabolism (lysine)	18.35 ± 16.73	2841.69 ± 3251.91	21300.21 ± 18672.92	TH
K01666	4-hydroxy 2-oxovalerate aldolase [EC:4.1.3.39]	Aromatic amino acid degradation	249.33 ± 303.97	1665.44 ± 1364.51	10813.54 ± 9177.22	TH
K00263	leucine dehydrogenase [EC:1.4.1.9]	Amino acid metabolism (leucine)	4278.67 ± 4745.48	18235.64 ± 6926.26	12935.94 ± 8265.81	HH
K01709	CDP-glucose 4,6-dehydratase [EC:4.2.1.45]	Carbohydrate metabolism	35.34 ± 25.87	1543.56 ± 1542.28	10815.50 ± 9119.81	TH
K00929	butyrate kinase [EC:2.7.2.7]	Short-chain fatty acid metabolism	7.34 ± 10.28	1354.00 ± 1428.33	10601.59 ± 9358.04	TH
K00048	lactaldehyde reductase [EC:1.1.1.77]	Carbohydrate metabolism	0.96 ± 1.60	45.09 ± 74.41	8.32 ± 10.53	HH
K00446	catechol 2,3-dioxygenase [EC:1.13.11.2]	Aromatic compound degradation	10.68 ± 18.34	850.08 ± 477.44	2242.63 ± 2629.26	TH
K00174	2-oxoglutarate/2-oxoacid ferredoxin oxidoreductase subunit alpha [EC:1.2.7.3 1.2.7.11]	Energy metabolism (TCA cycle)	519.50 ± 390.61	11162.74 ± 8629.42	15098.71 ± 5289.34	TH
K00004	(R,R)-butanediol dehydrogenase [EC:1.1.1.4 1.1.1.- 1.1.1.303]	Carbohydrate metabolism (butanediol)	472.67 ± 508.89	6255.19 ± 7083.83	11021.45 ± 8993.79	TH
K00978	glucose-1-phosphate cytidylyltransferase [EC:2.7.7.33]	Carbohydrate/nucleotide sugar metabolism	65.01 ± 50.92	1795.91 ± 1551.51	11291.00 ± 8585.09	TH
K00472	prolyl 4-hydroxylase [EC:1.14.11.2]	Amino acid metabolism (proline)	4.36 ± 7.49	1317.67 ± 1385.66	10593.00 ± 9361.39	TH

Supplementary Table 13. KEGG functional annotation and differential abundance of proteins involved in metabolism/digestion across WH, HH, and TH groups (continued)

KO ID	KO_Description	Pathway/Function	WHS (Mean ± SD)	HHS (Mean ± SD)	THS (Mean ± SD)	Dominant Group
K01222	6-phospho-beta-glucosidase [EC:3.2.1.86]	Carbohydrate digestion (cellobiose)	14.73 ± 17.95	1637.74 ± 1481.88	10924.66 ± 8946.52	TH
K01652	acetolactate synthase I/II/III large subunit [EC:2.2.1.6]	Amino acid biosynthesis (BCAA)	143364.7 ± 119083.8	202601.2 ± 38244.5	93306.0 ± 15707.8	HH
K00366	ferredoxin-nitrite reductase [EC:1.7.7.1]	Nitrogen metabolism	55.00 ± 55.08	1357.73 ± 1460.92	16645.63 ± 2651.71	TH

Supplementary Table 14. KEGG functional annotation and differential abundance of proteins involved in xenobiotics / drug metabolism across WH, HH, and TH groups

KO ID	Description	Category	WH Mean±SD	HH Mean±SD	TH Mean±SD	Dominant Group
K00128	aldehyde dehydrogenase (NAD+)	Xenobiotics degradation	54384±62878	130088±27214	72908±4394	HH
K00130	betaine-aldehyde dehydrogenase	Xenobiotics degradation	1115±615	21024±26443	9200±7102	HH
K00480	salicylate hydroxylase	Xenobiotics degradation	13921±16638a	25565±4633	8637±4923	HH
K00481	p-hydroxybenzoate monooxygenase	Xenobiotics degradation	361±282	6427±7046	3823±3115	HH
K00491	nitric-oxide synthase	Xenobiotics metabolism	51±70b	6470±9066	10685±6599	TH
K00496	alkane 1-monooxygenase	Xenobiotics degradation	1037±970	4991±2475	7725±6327	TH
K00537	arsenate reductase	Xenobiotics metabolism	46953±3248	45423±4617	25271±1954	
K00569	thiopurine S-methyltransferase	Drug metabolism	13685±17098	18986±9733	2832±215	HH
K00638	chloramphenicol acetyltransferase	Drug resistance	13678±17095	20325±9014	12769±6920	HH
K00663	aminoglycoside acetyltransferase	Drug resistance	261±311	1968±1330	11770±5661	TH

Supplementary Table 14. KEGG functional annotation and differential abundance of proteins involved in xenobiotics / drug metabolism across WH, HH, and TH groups (continued)

KO ID	Description	Category	WH Mean±SD	HH Mean±SD	TH Mean±SD	Dominant Group
K01130	arylsulfatase	Xenobiotics metabolism	17819±21258	36760±7476	14169±8502	HH
K01560	haloacid dehalogenase	Xenobiotics degradation	43318±3002	35862±5980	36115±3380	TH
K04091	alkanesulfonate monooxygenase	Xenobiotics metabolism	14656±17765	26445±3221	15738±4740	HH
K05595	multiple antibiotic resistance protein	Drug resistance	31666±37774a	55625±11272	14999±7188	HH

Supplementary Table 15. KEGG functional annotation and differential abundance of proteins involved in aerobic denitrification, assimilatory nitrate reduction, ammonium assimilation, and sulfur oxidation across WH, HH, and TH groups

KO ID	KO_Description	Process	Function/Role	WHS (Avg ± SD)	HHS (Avg ± SD)	THS (Avg ± SD)	Dominant Group
K00368	nitrite reductase (NO-forming) [EC:1.7.2.1]	Denitrification	Reduces nitrite (NO_2^-) to nitric oxide (NO) - key step in denitrification	13523 ± 16114	18815 ± 9486	2528 ± 20	HH
K04561	nitric oxide reductase subunit B [EC:1.7.2.5]	Denitrification	Reduces nitric oxide (NO) to nitrous oxide (N_2O) - detoxifies NO and completes denitrification	15601 ± 15780	16459 ± 9866	5327 ± 2331	HH
K00370	nitrate reductase / nitrite oxidoreductase, alpha subunit [EC:1.7.5.1 1.7.99.-]	Denitrification	Reduces nitrate (NO_3^-) to nitrite (NO_2^-) - first step of denitrification	13714 ± 15767	24209 ± 3056	13141 ± 6686	HH
K00371	nitrate reductase / nitrite oxidoreductase, beta subunit [EC:1.7.5.1 1.7.99.-]	Denitrification	Electron transfer subunit of nitrate reductase	13699 ± 15759	24194 ± 3096	13197 ± 6639	HH

Supplementary Table 15. KEGG functional annotation and differential abundance of proteins involved in aerobic denitrification, assimilatory nitrate reduction, ammonium assimilation, and sulfur oxidation across WH, HH, and TH groups (continued)

**KO ID**	**KO_Description**	**Process**	**Function/Role**	**WHS (Avg ± SD)**	**HHS (Avg ± SD)**	**THS (Avg ± SD)**	**Dominant Group**
K00372	assimilatory nitrate reductase catalytic subunit [EC:1.7.99.-]	Assimilatory nitrate reduction	Catalyzes reduction of nitrate to nitrite for incorporation into biomass	354 ± 285	3380 ± 2004	3870 ± 3056	TH
K00362	nitrite reductase (NADH) large subunit [EC:1.7.1.15]	Assimilatory nitrite reduction	Reduces nitrite to ammonium using NADH as electron donor	17547 ± 20388	33004 ± 4398	17279 ± 3889	HH
K00363	nitrite reductase (NADH) small subunit [EC:1.7.1.15]	Assimilatory nitrite reduction	Electron transfer subunit of NADH-dependent nitrite reductase	17506 ± 20352	32813 ± 4407	16786 ± 4255	HH
K01915	glutamine synthetase [EC:6.3.1.2]	Ammonium assimilation	Incorporates ammonium into glutamate to form glutamine - central nitrogen assimilation	67001 ± 24057	92134 ± 16944	67266 ± 32125	HH
K00265	glutamate synthase (NADPH) large chain [EC:1.4.1.13]	Ammonium assimilation	Glutamine:2-oxoglutarate aminotransferase - synthesizes two glutamates from glutamine and 2-oxoglutarate	17894 ± 20898	38533 ± 7723	21673 ± 425	HH
K00266	glutamate synthase (NADPH) small chain [EC:1.4.1.13]	Ammonium assimilation	Electron transfer subunit of glutamate synthase	18865 ± 20728	42646 ± 11922	25704 ± 17107	HH
K00956	sulfate adenylyltransferase subunit 1 [EC:2.7.7.4]	Sulfur oxidation	Converts sulfate to APS (adenosine-5'-phosphosulfate) - activation step	17191 ± 20321	24202 ± 12200	3472 ± 146	HH
K17218	sulfide:quinone oxidoreductase [EC:1.8.5.4]	Sulfur oxidation	Oxidizes sulfide to elemental sulfur or sulfite, transferring electrons to quinone	28044 ± 32310	44609 ± 11883	31514 ± 5851	HH

Supplementary Table 15. KEGG functional annotation and differential abundance of proteins involved in aerobic denitrification, assimilatory nitrate reduction, ammonium assimilation, and sulfur oxidation across WH, HH, and TH groups (continued)

KO ID	KO_Description	Process	Function/Role	WHS (Avg ± SD)	HHS (Avg ± SD)	THS (Avg ± SD)	Dominant Group
K16937	thiosulfate dehydrogenase (quinone) large subunit [EC:1.8.5.2]	Sulfur oxidation	Oxidizes thiosulfate to tetrathionate - energy-generating sulfur oxidation	14 ± 13	6228 ± 4599	21667 ± 12783	HH
K17226	sulfur-oxidizing protein SoxY	Sulfur oxidation	Carrier protein in the Sox multi-enzyme system for thiosulfate oxidation	550 ± 236	2473 ± 225	6280 ± 4907	TH
K17227	sulfur-oxidizing protein SoxZ	Sulfur oxidation	Carrier protein in the Sox multi-enzyme system for thiosulfate oxidation	279 ± 173	2352 ± 349	4726 ± 3776	TH
