# Supplementary material for: Lactobacillus salivarius GZPH2 reshapes hepatopancreatic microbiome structure and enhances immunometabolism in Litopenaeus vannamei under farm conditions
Source: Front Microbiol. 2026 Apr 16;17:1762396. doi: 10.3389/fmicb.2026.1762396 (PMC13128629; doi:10.3389/fmicb.2026.1762396)
Supplement: Supplementary file 3 [file Data_Sheet_3.pdf]

Supplementary note 1. Growth performance calculations

1.1 Body weight gain (g):

Individual weight gain (g) was calculated as:

$$\text{Body weight gain(g)} = \text{Final body weight (g)} - \text{Initial body weight(g)}$$

1.2 Total biomass gain (Kg):

$$\text{Total biomass gain (Kg)} = \frac{(\text{FMBW} - \text{IMBW}) \times \text{Number of survuving shrimp}}{1000}$$

Here,
Division by 1000 converts grams to kilograms
FNBW= Final mean body weight
IMBW= Initial mean body weight

1.3 Feed Conversion ratio (FCR):

$$\text{FCR} = \frac{\text{Total feed input (Kg)}}{\text{Total biomass gain (Kg)}}$$

Supplementary note 2. Probiotic sources and preparation

2.1 Commercial effective microorganisms (EM) probiotic

The commercial probiotic used was EM Probiotic Concentrate Powder (Shandong Jida Biotechnology Co., Ltd.; Production license: Lu Si Tian (2021) H14543), a consortium of *Lactobacillus*, *Bacillus*, nitrifying bacteria, *Actinobacteria*, and photosynthetic bacteria with a guaranteed potency of $\geq 1.0 \times 10^{11}$ CFU/g (Supplementary Figure 2). Prior to application, the product was activated by anaerobically fermenting a mixture of 200 g powder, 500 g brown sugar, and 20 L dechlorinated water at 28-30°C for 5 days. The resulting Activated Probiotic Solution (APS), which had a pH of approximately 4.0, was used within three weeks and was added to feed at a ratio of 1 kg APS per 50 kg of feed.

2.2 Experimental probiotic *Lactobacillus salivarius* strain GZPH2

La. salivarius strain GZPH2, the subject of Chinese Patent ZL201410752144.2, was used in this study. The strain was originally isolated from a commercially sourced pickle in Guangzhou, China. It was identified via 16S rDNA sequencing (sequence data available at: https://wenju.baidu.com/view/2d5d8eff6e175f0e7cd184254b35eefdc9d315ef) and deposited in the China Type Culture Collection under accession number CCTCC M 2014598. As a critical safety assessment, the strain was confirmed to be non-hemolytic on goat blood agar plates (data not shown). A frozen stock vial GZPH2 of was reactivated and cultured according to a modified protocol from Chinese Patent

ZL202310736625.3 (Supplementary Figure 3). Briefly, GZPH2 was cultivated in de Man, Rogosa, and Sharpe (MRS) broth and sub-cultured three times on MRS agar to obtain a pure culture. The medium contained (per 1 L): Glucose (40.0 g), Peptone (20.0 g), Beef Extract Powder (20.0 g), Yeast Extract (20.0 g), Sodium Acetate (20.0 g), K_2HPO_4 (8.0 g), Diammonium Hydrogen Citrate (8.0 g), NaCl (15 g), $MgSO_4 \cdot 7H_2O$ (0.4 g), $MnSO_4 \cdot 7H_2O$ (1 g), and Tween-80 (1 ml). The pH was adjusted to 6.5 ± 0.1 before autoclaving at 121°C and 0.1 MPa for 20 minutes. For solid cultures, MRS broth was supplemented with 15.0 g/L bacteriological agar. A seed stock was prepared by inoculating 1 mL of this activated culture into 50 mL of MRS broth and incubating statically at 37°C for 24 hours. Bacterial concentration was determined via serial dilution and spread plating on MRS agar; plates with 30-300 colonies were counted and expressed as colony-forming units per mL (CFU/mL). This seed stock reached a final concentration of approximately 8×10^8 CFU/mL and was stored at 4°C for future re-culturing. To prepare fresh probiotic for feed supplementation, 20 mL of the seed stock was inoculated into 1000 mL of MRS broth and incubated statically at 37°C for 24 hours. The resulting bacterial culture was mixed with commercial shrimp feed at a 1:3 (v/w) ratio. The coated feed was spread thinly and air-dried at 37°C for 1-3 hours. The probiotic-supplemented feed was prepared twice weekly and stored in sealed plastic bags at 4°C until use.
